# Solid-phase synthesis of biaryl bicyclic peptides containing a 3-aryltyrosine or a 4-arylphenylalanine moiety

**DOI:** 10.3762/bjoc.15.72

**Published:** 2019-03-22

**Authors:** Iteng Ng-Choi, Àngel Oliveras, Lidia Feliu, Marta Planas

**Affiliations:** 1 LIPPSO, Departament de Química, University of Girona, Maria Aurèlia Capmany 69, Girona 17003, Spain

**Keywords:** borylation, cross-coupling, cyclization, macrocycles, solid-phase synthesis

## Abstract

A methodology for the solid-phase synthesis of biaryl bicyclic peptides containing a Phe-Phe, a Phe-Tyr or a Tyr-Tyr motif has been devised. This approach comprises two key steps. The first one involves the cyclization of a linear peptidyl resin containing the corresponding halo- and boronoamino acids via a microwave-assisted Suzuki–Miyaura cross coupling. This step is followed by the macrolactamization of the resulting biaryl monocyclic peptidyl resin leading to the formation of the expected biaryl bicyclic peptide. This study provides the first solid-phase synthesis of this type of bicyclic compounds being amenable to prepare a diversity of synthetic or natural biaryl bicyclic peptides.

## Introduction

Monocyclic and bicyclic peptides are acquiring a relevant interest in current drug discovery. They display improved biological properties over their acyclic counterparts and, at the same time, they are suitable to modulate protein–protein interactions [1–9]. These advantageous attributes rise from the rigidity of their conformational structure and from the low susceptibility to protease degradation of the cyclic backbone.

There is a wide range of methods for the macrocyclization of linear peptides [1–2,10–14]. The most frequently used are the formation of an amide bond between the N- and the C-terminus and the cyclization involving the side chain of two amino acids. The latter is considered more convenient, because it does not interfere on hydrogen bonding between the N- and C-terminal groups of the peptide and their putative target. This method has been used for the macrocyclization of peptides through, for example, copper-catalyzed azide–alkyne cycloadditions [14], ring-closing olefin metathesis [13] or the formation of an aryl–aryl bond between the side chain of two aromatic amino acids [11].

Cyclic peptides containing biaryl linkages constitute attractive targets. On the one hand, a wide range of biaryl natural products have been reported to display interesting biological properties, such as biphenomycins, arylomycins and glycopeptide antibiotics [4]. On the other hand, the cyclization of linear peptides through aryl–aryl bond formation confers a relative conformational constraint on the peptide scaffold. Moreover, the resulting biaryl motif is able to participate in π–π interactions with aromatic and hydrophobic residues, and also in π-cation interactions with positively charged groups. Due to these structural properties, biaryl cyclic peptides have a great potential to meet the ever-increasing expectations of new drugs. However, their synthesis is viewed as very challenging.

The formation of biaryl bonds in peptides has been performed through a Suzuki–Miyaura cross-coupling reaction [15–19] or via a Pd-catalyzed C–H activation reaction [20–21]. We have used the former reaction for the solid-phase preparation of biaryl cyclic peptides bearing a Phe-Phe, a Phe-Tyr or a Tyr-Tyr linkage [22–23]. Our approach involved the synthesis of the linear peptidyl resin precursor containing the required boronate and halogenated amino acid derivatives followed by its cyclization through the formation of an aryl–aryl bond between these two amino acids via a Suzuky–Miyaura reaction. It is worthwhile to mention that both the borylation and the cross-coupling steps were performed on the solid support.

In this context, herein our aim was to extend our expertise in the formation of biaryl linkages to the solid-phase synthesis of biaryl bicyclic peptides. To the best of our knowledge, there is only one example on the preparation of this type of compounds on solid support, even though the final cyclization was performed in solution [24–25]. In contrast, in the present study we envisaged a synthetic strategy for the preparation of biaryl bicyclic peptides in which all the steps would be carried out on solid phase. It would benefit from the advantages intrinsic to the solid-supported chemistry, such as the avoidance of tedious work-up procedures, and the facile elimination by filtration of reagents and byproducts generated during the reactions. Therefore, this work would constitute the first synthetic approach on solid support of biaryl bicyclic peptides. Moreover, it would allow the access to a wide variety of sequences in a flexible manner.

**Figure 1:**
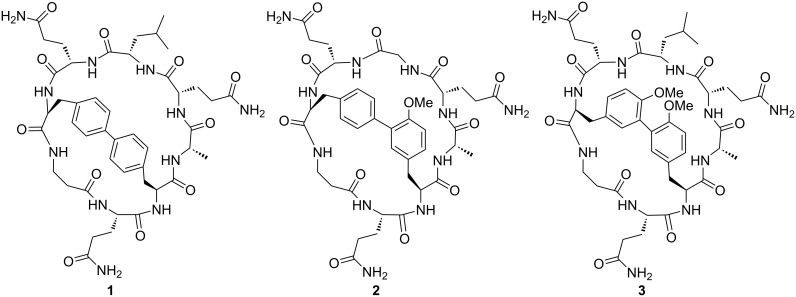
Structure of biaryl bicyclic peptides **1–3**.

To set up our strategy the biaryl bicyclic octapeptides **1–3** were chosen as model substrates (Figure 1). They contain commercially available L-amino acids and a different biaryl bond, in particular, a Phe-Phe, a Phe-Tyr or a Tyr-Tyr linkage.

## Results and Discussion

### Synthesis of the biaryl bicyclic peptide **1**

**Scheme 1:**
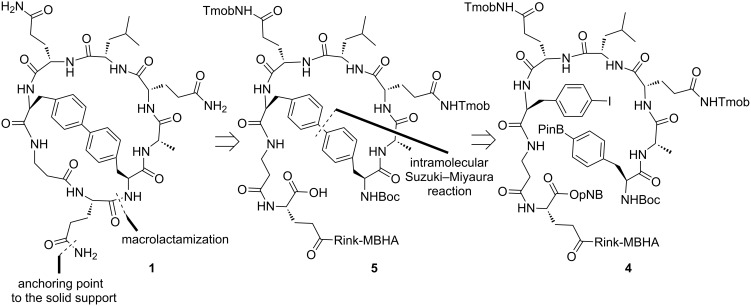
Retrosynthetic analysis for the biaryl bicyclic peptide **1**.

We first planned the synthesis of the biaryl bicyclic peptide **1** incorporating a Phe-Phe linkage based on the retrosynthetic analysis depicted in Scheme 1. According to this analysis, the key steps are the macrolactamization and the intramolecular Suzuki–Miyaura cross coupling. Another crucial issue is the selection of the anchoring point to the solid support. The glutamine residue placed at the southern hemisphere of **1** was chosen for this purpose. Thus, the synthesis of **1** would involve the preparation of the linear peptidyl resin **4** bearing a 4-iodo- and a 4-boronophenylalanine residue. The latter would be incorporated at the N-terminus of the peptide sequence which would avoid the decomposition of the boronic ester during the coupling steps [26]. The intramolecular Suzuki–Miyaura cross coupling of **4** followed by macrolactamization of the resulting biaryl monocyclic peptidyl resin **5** would afford **1**.

**Scheme 2:**
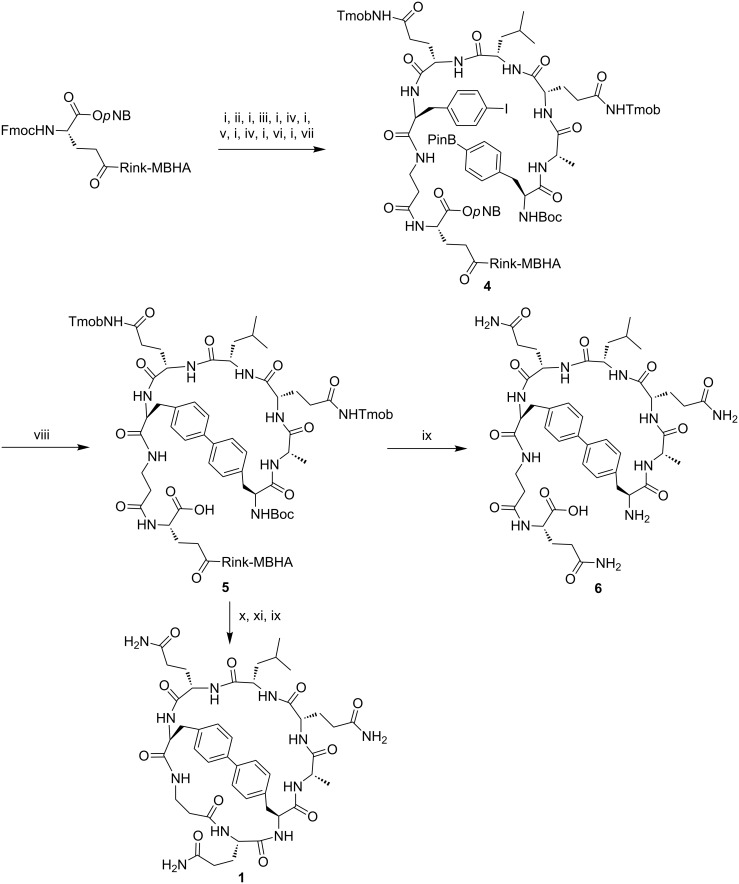
Synthesis of the biaryl bicyclic peptide **1** incorporating a Phe-Phe linkage. Reagents and conditions: (i) Piperidine/DMF (3:7). (ii) Fmoc-βAla-OH, DIPCDI, Oxyma, DMF. (iii) Fmoc-Phe(4-I)-OH, COMU, Oxyma, DIPEA, DMF, overnight. (iv) Fmoc-Gln(Tmob)-OH, DIPCDI, Oxyma, DMF. (v) Fmoc-Leu-OH, DIPCDI, Oxyma, DMF. (vi) Fmoc-Ala-OH, DIPCDI, Oxyma, DMF. (vii) Boc-Phe(4-BPin)-OH, DIPCDI, Oxyma, DMF, 3 h. (viii) Pd_2_(dba)_3_, P(*o*-tolyl)_3_, KF, DME/EtOH/H_2_O (9:9:2), MW, 120 °C, 30 min. (ix) TFA/H_2_O/TIS (95:2.5:2.5), 2 h. (x) TMSOTf, 2,6-lutidine, CH_2_Cl_2_. (xi) PyOxim, Oxyma, DIPEA, NMP, 24 h.

Based on the above, the protected linear peptidyl resin Boc-Phe(4-BPin)-Ala-Gln(Tmob)-Leu-Gln(Tmob)-Phe(4-I)-βAla-Glu(Rink-MBHA)-O*p*NB (**4**, *p*NB is *p*-nitrobenzyl) was assembled starting from an Fmoc-Rink-MBHA resin using the standard 9-fluorenylmethoxycarbonyl (Fmoc)/*tert*-butyl (*t-*Bu) strategy (Scheme 2). The non-commercially available amino acids Boc-Phe(4-BPin)-OH, Fmoc-Phe(4-I)-OH and Fmoc-Glu-O*p*NB were prepared in solution. Boc-Phe(4-BPin)-OH was obtained from Boc-Phe(4-I)-OH [27] through esterification, Miyaura borylation and hydrolysis of the ester. Fmoc-Phe(4-I)-OH was prepared by treating H-Phe(4-I)-OH [27] with Fmoc-OSu in dioxane. Fmoc-Glu-O*p*NB was synthesized from Fmoc-Glu(O*t-*Bu)-OH through *p*NB ester formation and subsequent removal of the *t-*Bu group [28–29]. The peptide chain was elongated through sequential Fmoc group removal and coupling steps. The Fmoc group was removed using piperidine/DMF (3:7). The coupling of amino acids was mediated by *N,N*’-diisopropylcarbodiimide (DIPCDI) and ethyl 2-cyano-2-(hydroxyimino)acetate (Oxyma) in DMF, except for Fmoc-Glu-O*p*NB and Fmoc-Phe(4-I)-OH, which were coupled using 1-[(1-cyano-2-ethoxy-2-oxoethylidineaminooxy)dimethylaminomorpholino]uronium hexafluorophosphate (COMU), Oxyma and *N,N’*-diisopropylethylamine (DIPEA) in DMF overnight. After peptide elongation, an aliquot of the resulting resin **4** was treated with trifluoroacetic acid (TFA)/triisopropylsilane (TIS)/H_2_O (95:2.5:2.5) for 2 h affording the linear boronopeptide H-Phe(4-B(OH)_2_)-Ala-Gln-Leu-Gln-Phe(4-I)-βAla-Gln-O*p*NB in >99% purity, which was characterized by mass spectrometry. The boronic acid function resulted from hydrolysis of the boronic ester during HPLC analysis as shown by mass spectrometry.

With the linear peptidyl resin **4** in hand, we investigated its cyclization by means of an intramolecular Suzuki–Miyaura reaction. Thus, based on our previous results on the synthesis of biaryl cyclic peptides [22], **4** was treated with Pd_2_(dba)_3_ (0.2 equiv), P(*o*-tolyl)_3_ (0.4 equiv) and KF (4 equiv) in degassed 1,2-dimethoxyethane (DME)/EtOH/H_2_O (9:9:2) under microwave irradiation at 120 °C for 30 min (Scheme 2). An aliquot of the resulting resin **5** was cleaved, and HPLC and mass spectrometry analysis of the crude reaction mixture revealed the formation of the biaryl cyclic peptide **6** in 18% purity. Mass spectrometry analysis showed that the *p*NB group was removed under the Suzuki–Miyaura reaction conditions.

To obtain the biaryl bicyclic peptide **1**, the Boc group of cyclic peptidyl resin **5** was then removed under mild conditions using trimethylsilyl triflate (TMSOTf) in presence of 2,6-lutidine in CH_2_Cl_2_ (Scheme 2) [30]. Subsequent macrolactamization was performed using *O*-(((1-cyano-2-ethoxy-2-oxoethylidene)amino)oxy)trispyrrilidin-1-yl)phosphonium hexafluorophosphate (PyOxim), Oxyma and DIPEA in *N*-methyl-2-pyrrolidone (NMP) for 24 h. The resulting resin was acidolytically cleaved and the crude reaction mixture was analyzed by HPLC and characterized by mass spectrometry. The latter revealed the formation of the expected biaryl bicyclic peptide **1** together with a less intense signal at [M − 18 + H]^+^, which was attributed to peptide fragmentation during the analysis, as confirmed by tandem mass spectrometry.

### Synthesis of the biaryl bicyclic peptide **2**

**Scheme 3:**
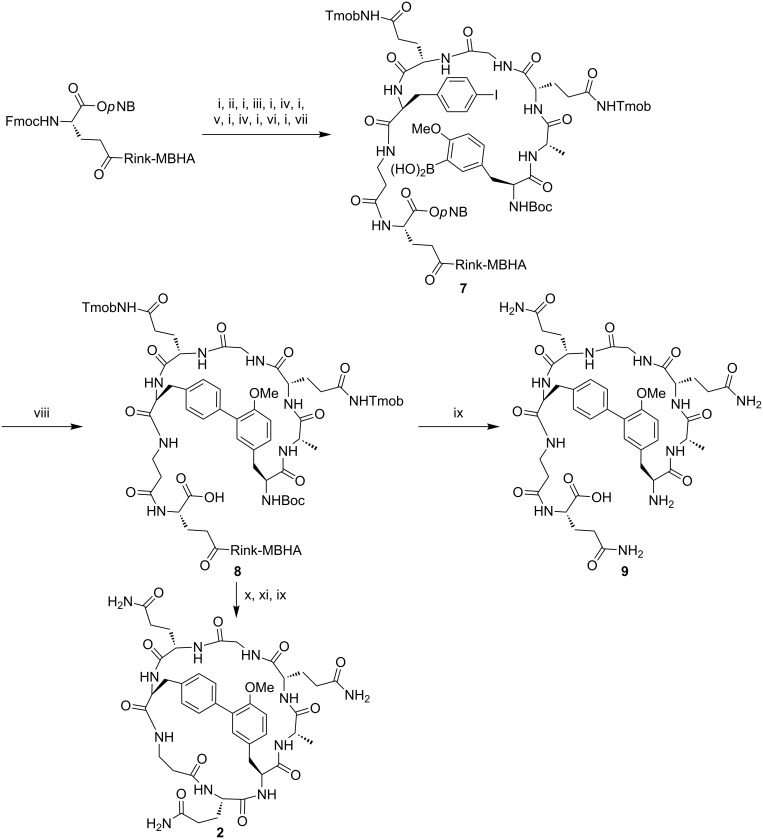
Synthesis of the biaryl bicyclic peptide **2** incorporating a Phe-Tyr linkage. Reagents and conditions: (i) Piperidine/DMF (3:7). (ii) Fmoc-βAla-OH, DIPCDI, Oxyma, DMF. (iii) Fmoc-Phe(4-I)-OH, COMU, Oxyma, DIPEA, DMF, overnight. (iv) Fmoc-Gln(Tmob)-OH, DIPCDI, Oxyma, DMF. (v) Fmoc-Gly-OH, DIPCDI, Oxyma, DMF. (vi) Fmoc-Ala-OH, DIPCDI, Oxyma, DMF. (vii) Boc-Tyr(3-B(OH)_2_,Me)-OH, DIPCDI, Oxyma, DMF, 3 h. (viii) Pd_2_(dba)_3_, SPhos, KF, DME/EtOH/H_2_O (9:9:2), MW, 120 °C, 30 min. (ix) TFA/H_2_O/TIS (95:2.5:2.5), 2 h. (x) TMSOTf, 2,6-lutidine, CH_2_Cl_2_. (xi) PyOxim, Oxyma, DIPEA, NMP, 24 h.

The synthesis of the biaryl bicyclic peptide **2**, which incorporates a Phe-Tyr linkage, was then investigated (Scheme 3). Similarly to **1**, the synthesis of **2** involved the preparation of the linear peptidyl resin Boc-Tyr(3-B(OH)_2_,Me)-Ala-Gln(Tmob)-Gly-Gln(Tmob)-Phe(4-I)-βAla-Glu(Rink-MBHA)-O*p*NB (**7**), followed by microwave-assisted intramolecular Suzuki–Miyaura reaction, Boc group removal, and final macrolactamization. Boc-Tyr(3-B(OH)_2_,Me)-OH was prepared in solution through Miyaura borylation of Boc-Tyr(3-I,Me)-OMe [31], followed by hydrolysis of the pinacolate and saponification of the methyl ester. Boc-Tyr(3-B(OH)_2_,Me)-OH was coupled using DIPCDI and Oxyma in DMF for 3 h. An aliquot of the linear resin **7** was cleaved to provide H-Tyr(3-B(OH)_2_,Me)-Ala-Gln-Gly-Gln-Phe(4-I)-βAla-Gln-O*p*NB in 57% purity, which was characterized by mass spectrometry. Resin **7** was then subjected to an intramolecular Suzuki–Miyaura reaction using Pd_2_(dba)_3_ (0.2 equiv), SPhos (0.4 equiv) and KF (4 equiv) in degassed DME/EtOH/H_2_O (9:9:2) under microwave irradiation at 120 ºC for 30 min. Mass spectrometry analysis of the crude reaction mixture from the cleavage of an aliquot of the resulting resin **8** revealed the formation of the expected biaryl monocyclic peptide **9** (36% purity) together with the oxidized and dehalogenated byproduct H-Tyr(3-OH,Me)-Ala-Gln-Gly-Gln-Phe-βAla-Gln-OH, which is usually formed in Suzuki–Miyaura reactions [32]. Finally, resin **8** was subjected to Boc removal, macrolactamization and acidolytic cleavage. Mass spectrometry analysis of the crude reaction mixture showed a signal at *m/z* corresponding to [M – 18 + H]^+^, which resulted from the fragmentation of the biaryl bicyclic peptide **2** during mass spectrometry analysis.

### Synthesis of the biaryl bicyclic peptide **3**

**Scheme 4:**
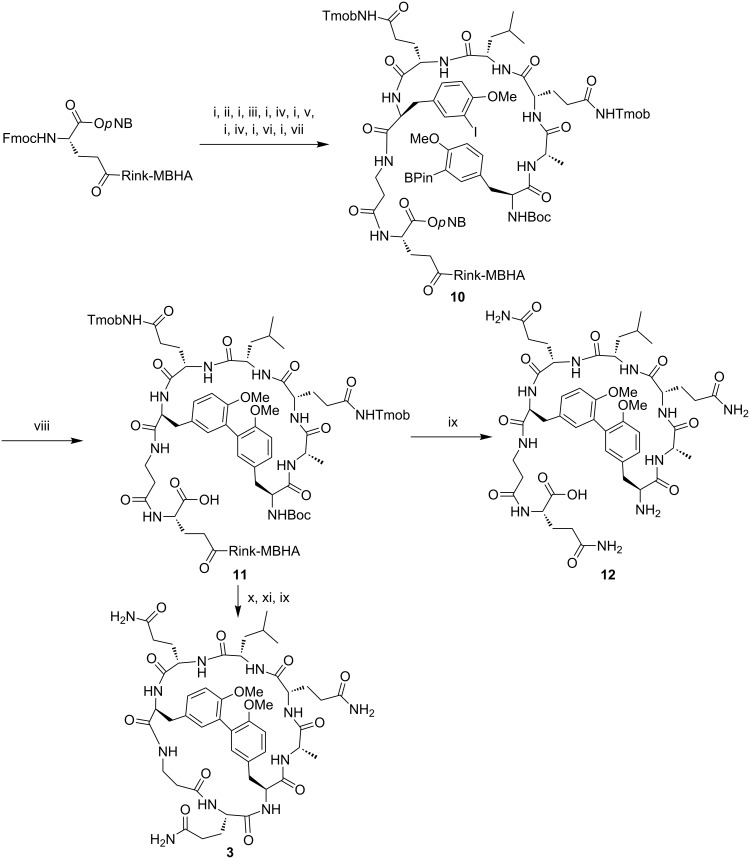
Synthesis of the biaryl bicyclic peptide **3** incorporating a Tyr-Tyr linkage. Reagents and conditions: (i) Piperidine/DMF (3:7). (ii) Fmoc-βAla-OH, DIPCDI, Oxyma, DMF. (iii) Fmoc-Tyr(3-I,Me)-OH, COMU, Oxyma, DIPEA, DMF, overnight. (iv) Fmoc-Gln(Tmob)-OH, DIPCDI, Oxyma, DMF. (v) Fmoc-Leu-OH, DIPCDI, Oxyma, DMF. (vi) Fmoc-Ala-OH, DIPCDI, Oxyma, DMF. (vii) Boc-Tyr(3-B(OH)_2_,Me)-OH, DIPCDI, Oxyma, DMF, 3 h. (viii) Pd_2_(dba)_3_, SPhos, KF, DME/EtOH/H_2_O (9:9:2), MW, 120 °C, 30 min. (ix) TFA/H_2_O/TIS (95:2.5:2.5), 2 h. (x) TMSOTf, 2,6-lutidine, CH_2_Cl_2_. (xi) PyOxim, Oxyma, DIPEA, NMP, 24 h.

Similarly to **1** and **2**, the biaryl bicyclic peptide **3** bearing a Tyr-Tyr linkage was obtained from the linear peptidyl resin Boc-Tyr(3-B(OH)_2_,Me)-Ala-Gln(Tmob)-Leu-Gln(Tmob)-Tyr(3-I,Me)-βAla-Glu(Rink-MBHA)-O*p*NB (**10**) (Scheme 4). Fmoc-Tyr(3-I,Me)-OH was prepared in solution from Boc-Tyr(3-I,Me)-OMe [31] through Boc group removal followed by methyl ester hydrolysis and Fmoc protection of the *N*^α^-amino group. Fmoc-Tyr(3-I,Me)-OH was coupled using COMU, Oxyma and DIPEA in DMF overnight. An aliquot of resin **10** was cleaved providing the expected linear peptide in 98% purity. Microwave-assisted intramolecular Suzuki–Miyaura reaction of **10** was carried out using the same conditions for the macrocyclization of resin **7**. Mass spectrometry analysis of the crude reaction mixture from cleavage of an aliquot of the resulting **11** revealed the formation of the expected biaryl cyclic peptide **12** in 21% HPLC purity. Selective Boc group removal, macrolactamization and final cleavage yielded the biaryl bicyclic peptide **3**. Mass spectra showed a signal at [M + H]^+^ together with a major one at [M − 18 + H]^+^ attributed to the fragmentation of **3** during the analysis, as confirmed by tandem mass spectrometry.

## Conclusion

A methodology for the solid-phase synthesis of biaryl bicyclic peptides bearing a Phe-Phe, a Phe-Tyr or a Tyr-Tyr linkage is described. The synthesis includes the preparation of a biaryl monocyclic peptidyl resin through an intramolecular microwave-assisted Suzuki–Miyaura cross coupling which is followed by a final macrolactamization step. This work constitutes the first solid-phase synthetic approach to biaryl bicyclic peptides. The method described is general and allows access to a diversity of novel Phe- and Tyr-containing biaryl bicyclic peptides.

## Supporting Information

File 1 Experimental, synthesis, and characterization of all the compounds.
