# Supplementary material for: Solid-phase synthesis of biaryl bicyclic peptides containing a 3-aryltyrosine or a 4-arylphenylalanine moiety
Source: Beilstein J Org Chem. 2019 Mar 22;15:761–8. doi: 10.3762/bjoc.15.72 (PMC6444451; doi:10.3762/bjoc.15.72)

BEILSTEIN JOURNAL OF ORGANIC CHEMISTRY

Supporting Information

for

Solid-phase synthesis of biaryl bicyclic peptides containing a 3-aryltyrosine or a 4-arylphenylalanine moiety

Iteng Ng-Choi, Àngel Oliveras, Lidia Feliu and Marta Planas

Beilstein J. Org. Chem. **2019,** *15,* 761–768. doi:10.3762/bjoc.15.72

Experimental, synthesis, and characterization of all the compounds

License and Terms: This is a supporting information file under the terms of the Creative Commons Attribution License (http://creativecommons.org/licenses/by/4.0). Please note that the reuse, redistribution and reproduction in particular requires that the authors and source are credited.
The license is subject to the *Beilstein Journal of Organic Chemistry* terms and conditions: (https://www.beilstein-journals.org/bjoc)

General methods

Manual peptide synthesis was performed in polypropylene syringes (5 mL) fitted with a polyethylene porous disk. Solvents and soluble reagents were removed by suction. Most chemicals were purchased from commercial suppliers Sigma–Aldrich (Madrid, Spain), Iris Biotech GmbH (Marktredwitz, Germany), Scharlab (Sentmenat, Spain) or Panreac (Castellar del Vallès, Spain), and used without further purification.

Peptides were analyzed under standard analytical HPLC conditions with a Dionex liquid chromatography instrument composed of an UV–vis Dionex UVD170U detector, a P680 Dionex bomb, an ASI-100 Dionex automatic injector, and CHROMELEON 6.60 software. Detection was performed at 220 nm. Solvent A was 0.1% aq. TFA and solvent B was 0.1% TFA in CH_3CN. Analysis was carried out with a Kromasil 100 C_{18} (4.6 mm × 40 mm, 3.5 μm) column with 2–100% B over 7 min at a flow rate of 1 mL/min.

ESI-MS analyses were performed at the Serveis Tècnics de Recerca of the University of Girona with an Esquire 6000 ESI ion Trap LC/MS (Bruker Daltonics) instrument equipped with an electrospray ion source. The instrument was operated in the positive ESI(+) ion mode. Samples (5 μL) were introduced into the mass spectrometer ion source directly through an HPLC autosampler. The mobile phase (80:20 CH_3CN/H_2O at a flow rate of 100 μL/min) was delivered by a 1200 Series HPLC pump (Agilent). Nitrogen was employed as both the drying and nebulising gas.

HRMS were recorded on a Bruker MicroTof-QIITM instrument using an electrospray ionization source at the Serveis Tècnics de Recerca of the University of Girona. Samples were introduced into the mass spectrometer ion source by direct infusion using a syringe pump and were externally calibrated using sodium formate. The instrument was operated in the positive ion mode.

^1H and ^{13}C NMR spectra were measured with a Bruker 400 MHz NMR spectrometer at the Serveis Tècnics de Recerca of the University of Girona. Chemical shifts were reported as δ values (ppm) directly referenced to the solvent signal.

Microwave-assisted reactions were performed with a single mode Discover S-Class labstation microwave (CEM) (0–300 W). The time, temperature, and power were controlled with the Synergy software. The temperature was monitored through an infrared sensor in the floor of the cavity.

Synthesis of Boc-Phe(4-BPin)-OH

Boc-Phe(4-I)-OMe

Boc-Phe(4-I)-OH [1] (2.96 g, 7.57 mmol, 1 equiv) was added to a solution of Cs_2CO_3 (3.74 g, 11.35 mmol, 1.5 equiv) in anhydrous DMF (38 mL). The reaction mixture was stirred at room temperature for 15 min. After this time, iodomethane (1.14 mL, 18.16 mmol, 2.4 equiv) was added and the resulting mixture was stirred at room temperature overnight. The reaction mixture was then poured into cold water (150 mL). The resulting solution was adjusted to pH 4–5 by addition of aqueous 10% HCl, and the product was extracted with EtOAc/toluene (1:1, 4 × 50 mL). The organic layers were combined, washed with brine (60 mL), and dried over anhydrous magnesium sulfate. Removal of the solvent afforded a pale yellow oil, which was purified by column chromatography. Elution with hexane/EtOAc (15:1) gave Boc-Phe(4-I)-OMe [2] as a white solid (2.31 g, 75% yield). t_R = 9.40 min (94% purity). ^1H NMR (400 MHz, $CDCl_3$): δ = 7.61 [d, *J* = 8.2 Hz, 2 H, CH-3_{arom}, CH-5_{arom}], 6.87 [d, *J* = 8.2 Hz, 2 H, CH-2_{arom}, CH-6_{arom}], 4.97 [d, *J* = 7.2 Hz, 1 H, NH], 4.58-4.54 [m, 1 H, CH-α], 3.71 [s, 3 H, OCH_3], 3.07 [dd, *J* = 5.6 and 13.8 Hz, 1 H, CH_2-β], 2.97 [dd, *J* = 5.6 and 13.8 Hz, 1 H, CH_2-β], 1.41 [s, 9 H, $C(CH_3)_3$] ppm. ^{13}C NMR (75 MHz, $CDCl_3$): δ = 172.15 [$\mathit{C}O_2CH_3$], 155.10 [CONH], 137.71 [C-1_{arom}], 135.84 [CH-3_{arom}, CH-5_{arom}],

131.43 [CH-2_{arom}, CH-6_{arom}], 92.63 [C-4_{arom}], 80.20 [*C*(CH_3)$_3$], 54.29 [CH-α], 52.44 [OCH_3], 38.02 [CH_2-β], 28.39 [(*C*H_3)$_3$C] ppm. MS (ESI): *m/z* = 305.9 $[M – Boc + H]^+$.

Boc-Phe(4-BPin)-OMe

A solution of Boc-Phe(4-I)-OMe (800 mg, 1.97 mmol, 1 equiv) in degassed anhydrous DMSO (2.6 mL) was added to a solution of bis(pinacolato)diboron (B_2Pin_2, 1 g, 3.95 mmol, 2 equiv), $PdCl_2$(dppf) (97 mg, 0.12 mmol, 0.06 equiv), and KOAc (783 mg, 7.90 mmol, 4 equiv) in degassed anhydrous DMSO (10 mL). The mixture was stirred under nitrogen at 80 ºC for 24 h. After this time, H_2O (50 mL) was added and the resulting solution was extracted with EtOAc (3 × 30 mL). The combined organic extracts were washed with brine (3 × 25 mL), and dried over anhydrous magnesium sulfate. Removal of the solvent gave a dark brown oil, which was purified by column chromatography. Elution with hexane/EtOAc (18:1) afforded Boc-Phe(4-BPin)-OMe [3] as a colorless oil (693 mg, 87% yield). t_R = 8.20 min (94% purity). ^{1}H NMR (400 MHz, $CDCl_3$): δ = 7.73 [d, *J* = 8.0 Hz, 2 H, CH-3_{arom}, CH-5_{arom}], 7.12 [d, *J* = 8.0 Hz, 2 H, CH-2_{arom}, CH-6_{arom}], 4.94 [d, *J* = 8.0 Hz, 1 H, NH], 4.56-4.61 [m, 1 H, CH-α], 3.70 [s, 3 H, OCH_3], 3.05-3.15 [m, 2 H, CH_2-β], 1.41 [s, 9 H, C(CH_3)$_3$], 1.34 [s, 12 H, (CH_3)$_{BPin}$] ppm. ^{13}C NMR (75 MHz, $CDCl_3$): δ = 172.37 [*C*O_2CH_3], 155.21 [CONH], 139.35 [C-1_{arom}], 135.15 [CH-3_{arom}, CH-5_{arom}], 128.85 [CH-2_{arom}, CH-6_{arom}], 83.94 [C_{BPin}], 80.09 [*C*(CH_3)$_3$], 54.48 [CH-α], 52.37 [OCH_3], 38.56 [CH_2-β], 28.44 [(*C*H_3)$_3$C], 25.01 [(CH_3)$_{BPin}$] ppm. MS (ESI): *m/z* = 224.0 $[M – Boc + H]^+$.

Boc-Phe(4-BPin)-OH

An aqueous solution of LiOH (216 mg, 5.04 mmol, 3 equiv) in H_2O (3.30 mL) was added to a solution of Boc-Phe(4-BPin)-OMe (680 mg, 1.68 mmol, 1 equiv) in MeOH/THF (1:1, 6.60 mL). The reaction mixture was stirred at room temperature for 5 h. After this time, the organic solvents were evaporated under reduced pressure and H_2O (100 mL) was added to the resulting residue. The resulting solution was adjusted to pH 5 by addition of glacial AcOH followed by extraction with

EtOAc (4 × 30 mL). The organic layers were combined, washed with brine (50 mL), and dried over anhydrous magnesium sulfate. Removal of the solvent afforded Boc-Phe(4-BPin)-OH [4] as a white solid (602 mg, 92% yield). t_R = 6.43 min (98% purity). ^{1}H NMR (400 MHz, $CDCl_3$): δ = 7.75 [d, *J* = 7.8 Hz, 2 H, CH-3$_{arom}$, CH-5$_{arom}$], 7.19 [d, *J* = 7.8 Hz, 2 H, CH-2$_{arom}$, CH-6$_{arom}$], 4.94 [d, *J* = 8.0 Hz, 1 H, NH], 4.62-4.58 [m, 1 H, CH-α], 3.21 [dd, *J* = 5.2 and 13.6 Hz, 1 H, CH_2-β], 3.11 [dd, *J* = 6.0 and 13.6 Hz, 1 H, CH_2-β], 1.42 [s, 9 H, $C(CH_3)_3$], 1.34 [s, 12 H, $(CH_3)_{BPin}$] ppm. ^{13}C NMR (75 MHz, $CDCl_3$): δ = 175.79 [CO_2H], 155.54 [CONH], 139.25 [C-1$_{arom}$], 135.23 [CH-3$_{arom}$, CH-5$_{arom}$], 128.96 [CH-2$_{arom}$, CH-6$_{arom}$], 84.02 [C_{BPin}], 80.46 [$C(CH_3)_3$], 54.31 [CH-α], 37.90 [CH_2-β], 28.41 [$(CH_3)_3C$], 24.97 [$(CH_3)_{BPin}$] ppm. MS (ESI): *m/z* = 210.0 $[M – Boc + H]^+$.

Fmoc-Phe(4-I)-OH

A solution of H-Phe(4-I)-OH [1] (3 g, 10.31 mmol, 1 equiv) in dioxane (38 mL) was neutralized to pH 7–8 by addition of aqueous 10% Na_2CO_3. The reaction mixture was stirred at room temperature for 30 min and Fmoc-OSu (3.65 g, 10.82 mmol, 1.05 equiv) was then added. After stirring for 24 h at room temperature, the reaction mixture was concentrated in vacuo. EtOAc (50 mL) was added, and the solution was washed with 1 N HCl (30 mL) and H_2O (3 × 30 mL). The aqueous layers were combined and adjusted to pH 1 and the solution was washed with EtOAc (3 × 40 mL). All the organic layers were combined, washed with brine (30 mL) and dried over anhydrous magnesium sulfate. Removal of the solvent followed by digestion of the resulting precipitate in pentane/diethyl ether (1:1, 60 mL) for 2 h afforded Fmoc-Phe(4-I)-OH [5] as a white solid (3.21 g, 61% yield). t_R = 8.96 min (96% purity). ^{1}H NMR (400 MHz, [D_6]DMSO): δ = 12.78 [bb, 1 H, CO_2H], 7.88 [d, *J* = 7.2 Hz, 2 H, 2 CH$_{arom}$-Fmoc], 7.72 [d, *J* = 8.4 Hz, 1 H, NH], 7.61-7.65 [m, 4 H, 2 CH$_{arom}$-Fmoc, CH-3$_{arom}$, CH-4$_{arom}$], 7.39-7.43 [m, 2 H, 2 CH$_{arom}$-Fmoc], 7.27-7.33 [m, 2 H, 2 CH$_{arom}$-Fmoc], 7.08 [d, *J* = 8.0 Hz, 2 H, CH-2$_{arom}$, CH-6$_{arom}$], 4.12-4.22 [m, 4 H, CH-α, CH_2-Fmoc, CH-Fmoc], 3.04 [dd, *J* = 4.4 and 13.8 Hz, 1 H, CH_2-β], 2.82 [dd, *J* = 10.8 and 13.8 Hz, 1 H,

CH_2-β] ppm. ^{13}C NMR (75 MHz, [D_6]DMSO): δ = 173.13 [CO_2H], 155.93 [CONH], 143.77, 140.69, 137.84 [4 C_{arom}-Fmoc, C-1_{arom}], 136.91 [CH-3_{arom}, CH-5_{arom}], 131.61 [CH-2_{arom}, CH-6_{arom}], 127.63, 127.07, 125.23, 120.12 [8 CH_{arom}-Fmoc], 92.33 [C-4_{arom}], 65.59 [CH_2-Fmoc], 55.21 [CH-α], 46.57 [CH-Fmoc], 35.89 [CH_2-β] ppm. MS (ESI): *m/z* = 513.9 $[M + H]^+$, 536.0 $[M + Na]^+$.

Synthesis of Fmoc-Glu-O*p*NB

Fmoc-Glu(Ot-Bu)-OpNB

DIPCDI (1.18 mL, 7.64 mmol, 1 equiv) was added to a solution of Fmoc-Glu(O*t*-Bu)-OH (3.25 g, 8.40 mmol, 1.1 equiv) and *p*-nitrobenzyl alcohol (1.17 g, 7.64 mmol, 1 equiv) in CH_2Cl_2 (7.5 mL). The reaction mixture was stirred at room temperature and DMAP (0.09 g, 0.76 mmol, 0.1 equiv) was subsequently added. After stirring for 4 h at room temperature, EtOAc (40 mL) was added followed by washes with 10% aqueous Na_2CO_3 (3 × 25 mL) and brine (1 × 25 mL). The organic layer was dried over anhydrous $MgSO_4$. Removal of the solvent gave a yellow oil, which was purified by column chromatography. Elution with hexane/EtOAc (4:1) afforded Fmoc-Glu(O*t*-Bu)-OpNB [6] as a colorless oil (3 g, 70 % yield). t_R = 10.12 min (>99% purity). ^1H NMR (400 MHz, $CDCl_3$): δ = 8.19 [d, *J* = 8.4 Hz, 2 H, 2 CH_{arom}-*p*NB], 7.77 [d, *J* = 7.6 Hz, 2 H, 2 CH_{arom}-Fmoc], 7.58 [d, *J* = 7.2 Hz, 2 H, 2 CH_{arom}-Fmoc], 7.51 [d, *J* = 8.4 Hz, 2 H, 2 CH_{arom}-*p*NB], 7.42-7.38 [m, 2 H, 2 CH_{arom}-Fmoc], 7.32-7.26 [m, 2 H, 2 CH_{arom}-Fmoc], 5.51 [d, *J* = 8.0 Hz, 1 H, NH], 5.27 [s, 2 H, CH_2-*p*NB], 4.48-4.35 [m, 3 H, CH_2-Fmoc, CH-Fmoc], 4.22-4.18 [m, 1 H, CH-α], 2.40-2.28 [m, 2 H, CH_2-γ], 2.23-2.17 [m, 1 H, CH_2-β], 2.03-1.98 [m, 1 H, CH_2-β], 1.45 [s, 9 H, $C(CH_3)_3$] ppm.

Fmoc-Glu-OpNB

Fmoc-Glu(O*t*-Bu)-O*p*NB (1.50 g, 2.68 mmol, 1 equiv) was dissolved in TFA/CH_2Cl_2 (1:1, 25 mL) and stirred at room temperature for 2 h. After this time, the solvent mixture was removed under vacuum. Diethyl ether was then added and evaporated under vacuum. This process was repeated three times in order to completely remove the TFA. The resulting product was dried in vacuo overnight to afford Fmoc-Glu-O*p*NB [6] as a white solid (1.30 g, 96% yield). t_R = 8.75 min (95% purity). ^{1}H NMR (400 MHz, $CDCl_3$): δ = 8.19 [d, *J* = 8.4 Hz, 2 H, 2 CH_{arom}-*p*NB], 7.76 [d, *J* = 7.2 Hz, 2 H, 2 CH_{arom}-Fmoc], 7.57 [d, *J* = 7.2 Hz, 2 H, 2 CH_{arom}-Fmoc], 7.49 [d, *J* = 8.4 Hz, 2 H, 2 CH_{arom}-*p*NB], 7.41-7.39 [m, 2 H, 2 CH_{arom}-Fmoc], 7.32-7.28 [m, 2 H, 2 CH_{arom}-Fmoc], 5.42 [d, *J* = 8.4 Hz, 1 H, NH], 5.25 [s, 2 H, CH_2-*p*NB], 4.53-4.40 [m, 3 H, CH_2-Fmoc, CH-Fmoc], 4.21-4.17 [m, 1 H, CH-α], 2.50-2.39 [m, 2 H, CH_2-γ], 2.30-2.23 [m, 1 H, CH_2-β], 2.04-1.95 [m, 1 H, CH_2-β] ppm.

Synthesis of Boc-Tyr(3-B(OH)$_2$,Me)-OH

Boc-Tyr(3-B(OH)$_2$,Me)-OMe

A solution of Boc-Tyr(3-I,Me)-OMe [7] (920 mg, 2.11 mmol, 1 equiv) in degassed anhydrous DMSO (9 mL) was added to a solution of bis(pinacolato)diboron (B_2Pin_2) (1.08 g, 4.23 mmol, 2 equiv), $PdCl_2$(dppf) (100 mg, 0.12 mmol, 0.06 equiv), and KOAc (840 mg, 8.45 mmol, 4 equiv) in degassed anhydrous DMSO (4.5 mL). The mixture was stirred under nitrogen at 80 ºC for 7 h. After this time, brine (50 mL) was added and the resulting solution was extracted with EtOAc (3 × 50 mL). The combined organic extracts were washed with brine (3 × 50 mL), and dried over anhydrous magnesium sulfate. Removal of the solvent gave a dark brown oil, which was purified by column chromatography. Elution with hexane/EtOAc (4:1) afforded Boc-Tyr(3-B(OH)$_2$,Me)-OMe together with Boc-Tyr(3-BPin,Me)-OMe [8]. A solution of CH_3CN/H_2O (1:1) was then added and

stirred at 75 °C for 4 h. The resulting solution was lyophilized to afford a white solid, which was purified by column chromatography. Elution with hexane/EtOAc (1:1) yielded Boc-Tyr(3-$B(OH)_2$,Me)-OMe as a white solid (450 mg, 59% yield). t_R = 7.51 min (>99% purity). ^{1}H NMR (400 MHz, $CDCl_3$): δ = 7.57 [d, *J* = 2.8 Hz, 1 H, CH-2_{arom}], 7.20 [dd, *J* = 2.8 and 8.4 Hz, 1 H, CH-6_{arom}], 6.84 [d, *J* = 8.4 Hz, 1 H, CH-5_{arom}], 5.79 [bs, 2 H, $B(OH)_2$], 4.98 [bs, 1 H, CONH], 4.56-4.54 [m, 1 H, CH-α], 3.89 [s, 3 H, OCH_3], 3.73 [s, 3 H, CO_2CH_3], 3.09 [dd, *J* = 5.6 and 13.8 Hz, 1 H, CH_2-β], 3.01 [dd, *J* = 6.0 and 13.8 Hz, 1 H, CH_2-β], 1.41 [s, 9 H, $C(CH_3)_3$] ppm. ^{13}C NMR (75 MHz, $CDCl_3$): δ = 172.53 [$\mathit{C}O_2CH_3$] 163.85 [C-4_{arom}], 155.37 [CONH], 137.87 [CH-2_{arom}], 133.75, 128.64 [CH-6_{arom}, C-1_{arom}], 110.84, 110.34 [CH-5_{arom}, C-3_{arom}], 80.30 [$\mathit{C}(CH_3)_3$], 55.95 [OCH_3], 54.73 [CH-α], 52.41 [$CO_2\mathit{C}H_3$], 37.60 [CH_2-β], 28.41 [$(\mathit{C}H_3)_3C$] ppm.

Boc-Tyr(3-B(OH)$_2$,Me)-OH

An aqueous solution of LiOH (3 mL, 4.17 mmol, 3 equiv) was added to a solution of Boc-Tyr(3-$B(OH)_2$,Me)-OMe (450 mg, 1.27 mmol, 1 equiv) in MeOH/THF (1:1, 6 mL). The reaction mixture was stirred at room temperature for 1.5 h. After this time, the organic solvents were evaporated under reduced pressure and water (25 mL) was added to the resulting residue. The solution was adjusted to pH 5–6 by addition of 1 N HCl followed by extraction with EtOAc (3 × 25 mL). The organic layers were combined, washed with brine (25 mL), and dried over anhydrous magnesium sulfate. Removal of the solvent afforded Boc-Tyr(3-$B(OH)_2$,Me)-OH [8] as a white solid (410 mg, 95% yield). t_R = 6.84 min (>99% purity). ^{1}H NMR (400 MHz, $CDCl_3$): δ = 7.60 [s, 1 H, CH-2_{arom}], 7.25-7.22 [m, 1 H, CH-6_{arom}], 6.82 [d, *J* = 8.8 Hz, 1 H, CH-5_{arom}], 5.06-5.04 [m, 1 H, CONH], 4.51-4.49 [m, 1 H, CH-α], 3.87 [s, 2 H, OCH_3], 3.13 [dd, *J* = 4.8 and 13.6 Hz, 1 H, CH_2-β], 3.03 [dd, *J* = 5.2 and 13.6 Hz, 1 H, CH_2-β], 1.40 [s, 9 H, $C(CH_3)_3$] ppm. ^{13}C NMR (75 MHz, $CDCl_3$): δ = 175.45 [$\mathit{C}O_2H$] 163.74 [C-4_{arom}], 155.49 [CONH], 137.79 [CH-2_{arom}], 134.02, 128.57 [CH-6_{arom}, C-1_{arom}], 110.82, 110.30 [CH-5_{arom}, C-3_{arom}], 80.33 [$\mathit{C}(CH_3)_3$], 55.70 [OCH_3], 54.70 [CH-α], 37.28 [CH_2-β], 28.43 [$(\mathit{C}H_3)_3C$] ppm.

Synthesis of Fmoc-Tyr(3-I,Me)-OH

H-Tyr(3-I,Me)-OMe

Boc-Tyr(3-I,Me)-OMe [7] (4.50 g, 10.34 mmol, 1 equiv) was dissolved in TFA/CH_2Cl_2 (1:1, 30 mL) and stirred at room temperature for 2 h. After this time, the solvent mixture was removed under vacuum. Diethyl ether was then added and evaporated, this process was repeated three times to completely remove the TFA. The resulting product was dried in vacuo overnight to afford H-Tyr(3-I,Me)-OMe [8] as a white solid (3.25 g, 94% yield). t_R = 6.65 min (93% purity). ^{1}H NMR (400 MHz, $CDCl_3$): δ = 7.59 [d, *J* = 2.2 Hz, 1 H, CH-2_{arom}], 7.18 [dd, *J* = 2.2 and 8.4 Hz, 1 H, CH-6_{arom}], 6.76 [d, *J* = 8.4 Hz, 1 H, CH-5_{arom}], 4.18 [t, *J* = 6.6 Hz, 1 H, CH-α], 3.84 [s, 3 H, CO_2CH_3], 3.74 [s, 3 H, OCH_3], 3.16 [d, *J* = 6.6 Hz, 2 H, CH_2-β] ppm. ^{13}C NMR (75 MHz, [D_6]DMSO): δ = 169.47 [CO_2CH_3], 157.14 [C-4_{arom}], 139.79 [CH-2_{arom}], 130.89 [C-1_{arom}], 128.60 [CH-6_{arom}], 111.58 [CH-5_{arom}], 86.37 [C-3_{arom}], 56.44 [OCH_3], 53.20 [CH-α], 52.77 [CO_2CH_3], 34.47 [CH_2-β] ppm.

H-Tyr(3-I,Me)-OH

A solution of LiOH (1.24 g, 28.91 mmol, 3 equiv) in water (17 mL) was added to a solution of H-Tyr(3-I,Me)-OMe (3.23 g, 9.64 mmol, 1 equiv) in MeOH/THF (1:1, 34 mL). The reaction mixture was stirred at room temperature for 2 h. After this time, the organic solvents were evaporated under reduced pressure and water (60 mL) was added to the resulting residue. The solution was adjusted to pH 5 by addition of glacial AcOH and the resulting precipitate was filtered, washed with cold diethyl ether, and dried in vacuo overnight, yielding H-Tyr(3-I,Me)-OH as a white solid (3 g, 96% yield). t_R = 6.21 min (>99% purity). ^{1}H NMR (300 MHz, [D_6]DMSO): δ = 7.64 [d, *J* = 2.1 Hz, 1 H, CH-2_{arom}], 7.24 [dd, *J* = 2.1 and 8.4 Hz, 1 H, CH-6_{arom}], 6.91 [d, *J* = 8.4 Hz, 1 H, CH-5_{arom}], 3.78 [s, 3 H, OCH_3], 3.31 [dd, *J* = 4.5 and 7.7 Hz, 1 H, CH-α], 3.00 [dd, *J* = 4.5 and 14.1 Hz, 1 H, CH_2-β], 2.75 [dd, *J* = 7.7 and 14.1 Hz, 1 H, CH_2-β] ppm. ^{13}C NMR (75 MHz,

$[D_6]$DMSO): δ = 156.37 [C-4_{arom}], 139.60 [CH-2_{arom}], 132.22 [C-1_{arom}], 130.76 [CH-6_{arom}], 111.36 [CH-5_{arom}], 85.98 [C-3_{arom}], 56.33 [OCH_3], 55.74 [CH-α], 35.87 [CH_2-β] ppm.

Fmoc-Tyr(3-I,Me)-OH

A solution of H-Tyr(3-I,Me)-OH (3 g, 9.34 mmol, 1 equiv) in dioxane (32 mL) was adjusted to pH 7–8 by addition of aqueous 10% Na_2CO_3. The reaction mixture was stirred at room temperature for 30 min and Fmoc-OSu (3.31 g, 9.81 mmol, 1.05 equiv) was then added. The mixture was stirred for 24 h at room temperature and then concentrated in vacuo. EtOAc (40 mL) was added and the organic solution was washed with 1 N HCl (30 mL) and H_2O (3 × 30 mL). The aqueous layers were combined, adjusted to pH 1 and washed with EtOAc (3 × 40 mL). All the organic layers were combined, washed with brine (30 mL) and dried with anhydrous magnesium sulfate. Removal of the solvent followed by digestion of the resulting precipitate in pentane/diethyl ether (1:1, 50 mL) for 2 h afforded a white solid, which was purified by column chromatography. Elution with CH_2Cl_2/MeOH (95:5) gave Fmoc-Tyr(3-I,Me)-OH as a white solid (2.85 g, 57% yield). t_R = 9.00 min (>99% purity). ^{1}H NMR (400 MHz, $[D_6]$DMSO): δ = 12.78 [bb, 1 H, CO_2H], 7.88 [d, J = 7.6 Hz, 2 H, 2 CH_{arom}-Fmoc], 7.71 [d, J = 2.0 Hz, 1 H, CH-2_{arom}], 7.66-7.62 [m, 2 H, 2 CH_{arom}-Fmoc], 7.43-7.39 [m, 2 H, 2 CH_{arom}-Fmoc], 7.34-7.25 [m, 3 H, 2 CH_{arom}-Fmoc, CH-6_{arom}], 6.90 [d, J = 8.4 Hz, 1 H, CH-5_{arom}], 4.22-4.10 [m, 4 H, CH-α, CH_2-Fmoc, CH-Fmoc], 3.77 [s, 3 H, OCH_3], 3.01 [dd, J = 4.4 and 13.8 Hz, 1 H, CH_2-β], 2.77 [dd, J = 10.4 and 13.8 Hz, 1 H, CH_2-β] ppm. ^{13}C NMR (75 MHz, $[D_6]$DMSO): δ = 173.20 [CO_2H], 156.37 [C-4_{arom}], 155.97 [CONH], 143.74, 140.67 [4 C_{arom}-Fmoc], 139.46 [CH-2_{arom}], 132.20 [C-1_{arom}], 130.40 [CH-6_{arom}], 127.63, 127.10, 125.29, 120.11 [8 CH_{arom}-Fmoc], 111.28 [CH-5_{arom}], 85.73 [C-3_{arom}], 65.69 [CH_2-Fmoc], 56.27 [OCH_3], 55.61 [CH-α], 46.57 [CH-Fmoc], 34.95 [CH_2-β] ppm.

General procedure for the synthesis of octapeptidyl resins 4, 7 and 10

The octapeptidyl resins **4**, **7** and **10** were synthesized manually by the solid-phase method by using standard Fmoc chemistry. MBHA resin (0.4 mmol/g) was placed in a polypropylene syringe fitted with a polyethylene filter disk, and then was swollen with CH_2Cl_2 (1 × 20 min) and DMF (1 × 20 min), treated with piperidine/DMF (3:7, 1 × 5 min) and washed with DMF (6 × 1 min). The Fmoc-Rink amide linker (4 equiv) was coupled using DIPCDI (4 equiv) and Oxyma (4 equiv) in DMF under stirring at room temperature overnight. After this time, the resin was washed with DMF (6 × 1 min) and CH_2Cl_2 (3 × 1 min). Elongation of the peptide sequence was performed through sequential Fmoc removal and coupling steps. The Fmoc group was removed with a mixture of piperidine/DMF (3:7, 1 × 2 min and 1 × 10 min). Couplings of Fmoc-Glu-O*p*NB, Fmoc-Phe(4-I)-OH, or Fmoc-Tyr(3-I,Me)-OH (2 equiv) were perfomed using (1-cyano-2-ethoxy-2-oxoethylidenaminooxy)dimethylamino-morpholino-carbenium hexafluorophosphate (COMU) (2 equiv), ethyl (hydroxyimino)cyanoacetate (Oxyma, 2 equiv) and *N*,*N*'-diisopropylethylamine (DIPEA, 4 equiv) in DMF at room temperature overnight. The commercially available Fmoc-amino acids were incorporated using *N*,*N*'-diisopropylcarbodiimide (DIPCDI, 4 equiv) and Oxyma (4 equiv) in DMF at room temperature for 1 h. Coupling of Boc-Phe(4-BPin)-OH (3 equiv) or Boc-Tyr(3-$B(OH)_2$,Me)-OH (3 equiv) was mediated by DIPCDI (3 equiv) and Oxyma (3 equiv) in DMF at room temperature for 3 h. The completion of the reactions was monitored by the Kaiser test [9]. After each coupling and Fmoc group removal step, the resulting resin was washed with DMF (6 × 1 min). Once the peptidyl sequence was complete, the resin was washed with DMF (6 × 1 min), CH_2Cl_2 (3 × 1 min), and diethyl ether (3 × 1 min). An aliquot of the resulting resin was cleaved with TFA/H_2O/TIS (95:2.5:2.5) whilst being stirred for 2 h at room temperature. Following TFA evaporation and diethyl ether extraction, the crude peptide was dissolved in H_2O/CH_3CN, lyophilized, analysed by HPLC, and characterized by mass spectrometry.

Boc-Phe(4-BPin)-Ala-Gln(Tmob)-Leu-Gln(Tmob)-Phe(4-I)-βAla-Glu(Rink-MBHA)-OpNB (4)

This peptidyl resin was prepared using the procedure described above employing Fmoc-Glu-O*p*NB, Fmoc-Phe(4-I)-OH, and Boc-Phe(4-BPin)-OH. Acidolytic cleavage of an aliquot of the resulting resin Boc-Phe(4-BPin)-Ala-Gln(Tmob)-Leu-Gln(Tmob)-Phe(4-I)-βAla-Glu(Rink-MBHA)-O*p*NB (**4**) afforded H-Phe(4-B(OH)$_2$)-Ala-Gln-Leu-Gln-Phe(4-I)-βAla-Gln-O*p*NB in >99% purity, resulting from the hydrolysis of the pinacol boronic ester during HPLC analysis, as confirmed by mass spectrometry. t_R = 6.91 min. MS (ESI): *m/z* = 1257.4 $[M + H]^+$, 1279.3 $[M + Na]^+$.

Boc-Tyr(3-B(OH)$_2$,Me)-Ala-Gln(Tmob)-Gly-Gln(Tmob)-Phe(4-I)-βAla-Glu(Rink-MBHA)-OpNB (7)

This peptidyl resin was prepared using the procedure described above employing Fmoc-Glu-O*p*NB, Fmoc-Phe(4-I)-OH, and Boc-Tyr(3-B(OH)$_2$,Me)-OH. Acidolytic cleavage of an aliquot of the resulting resin Boc-Tyr(3-B(OH)$_2$,Me)-Ala-Gln(Tmob)-Gly-Gln(Tmob)-Phe(4-I)-βAla-Glu(Rink-MBHA)-O*p*NB (**7**) afforded H-Tyr(3-B(OH)$_2$,Me)-Ala-Gln-Gly-Gln-Phe(4-I)-βAla-Gln-O*p*NB in 57% purity, as confirmed by mass spectrometry. t_R = 6.55 min. MS (ESI): *m/z* = 1231.4 $[M + H]^+$, 1253.4 $[M + Na]^+$.

Boc-Tyr(3-B(OH)$_2$,Me)-Ala-Gln(Tmob)-Leu-Gln(Tmob)-Tyr(3-I,Me)-βAla-Glu(Rink-MBHA)-OpNB (10)

This peptidyl resin was prepared using the procedure described above employing Fmoc-Glu-O*p*NB), Fmoc-Tyr(3-I,Me)-OH, and Boc-Tyr(3-B(OH)$_2$,Me)-OH. Acidolytic cleavage of an aliquot of the resulting resin Boc-Tyr(3-B(OH)$_2$,Me)-Ala-Gln(Tmob)-Leu-Gln(Tmob)-Tyr(3-I,Me)-βAla-Glu(Rink-MBHA)-O*p*NB (**10**) afforded H-Tyr(3-B(OH)$_2$,Me)-Ala-Gln-Leu-Gln-Tyr(3-I,Me)-βAla-Gln-O*p*NB in 98% purity, as confirmed by mass spectrometry. t_R = 7.02 min. MS (ESI): *m/z* = 1317.5 $[M + H]^+$, 1339.5 $[M + Na]^+$.

General method for the synthesis of the biaryl cyclic peptidyl resins 5, 8 and 11 using a microwave-assisted solid-phase intramolecular Suzuki–Miyaura arylation

A 15 mL reaction vessel containing a magnetic stirring bar was charged with the corresponding linear octapeptidyl resin **4**, **7** or **10** (53–130 mg), $Pd_2(dba)_3$ (0.2 equiv), P(*o*-tolyl)$_3$ or SPhos (0.4 equiv), and KF (4 equiv). Thoroughly degassed DME/EtOH/H_2O (9:9:2, 0.30–0.73 mL) was then added under nitrogen. The reaction mixture was heated at 120 ºC under microwave irradiation for 30 min. After the reaction time, upon cooling, the solvent was removed and the resin was washed with DMF (6 × 1 min), EtOH (6 × 1 min), CH_2Cl_2 (6 × 1min), and diethyl ether (3 × 1 min). An aliquot of the resulting biaryl cyclic peptidyl resin was cleaved with TFA/H_2O/TIS (95:2.5:2.5) whilst being stirred for 2 h at room temperature. Following TFA evaporation and diethyl ether extraction, the crude peptide was dissolved in H_2O/CH_3CN (1:1), and lyophilized, analysed by HPLC, and characterized by mass spectrometry.

Biaryl cyclic peptidyl resin 5

This biaryl cyclic peptidyl resin was prepared from the linear peptidyl resin Boc-Phe(4-BPin)-Ala-Gln(Tmob)-Leu-Gln(Tmob)-Phe(4-I)-βAla-Glu(Rink-MBHA)-O*p*NB (**4**) (130 mg) following the procedure described above using P(*o*-tolyl)$_3$ as ligand. These conditions also led to the removal of the *p*NB group. Acidolytic cleavage of an aliquot of the resulting resin **5** afforded the biaryl cyclic peptide **6** in 18% purity. t_R = 5.66 min. MS (ESI): *m/z* = 950.4 $[M + H]^+$, 972.4 $[M + Na]^+$.

Biaryl cyclic peptidyl resin 8

This biaryl cyclic peptidyl resin was prepared from the linear peptidyl resin Boc-Tyr(3-B(OH)$_2$,Me)-Ala-Gln(Tmob)-Gly-Gln(Tmob)-Phe(4-I)-βAla-Glu(Rink-MBHA)-O*p*NB (**7**) (53 mg) following the procedure described above using SPhos as ligand. These conditions also led to the

removal of the *p*NB group. Acidolytic cleavage of an aliquot of the resulting resin **8** afforded the biaryl cyclic peptide **9** in 36% purity. t_R = 5.63 min. MS (ESI): *m/z* = 924.4 $[M + H]^+$.

Biaryl cyclic peptidyl resin 11

This biaryl cyclic peptidyl resin was prepared from the linear peptidyl resin Boc-Tyr(3-$B(OH)_2$,Me)-Ala-Gln(Tmob)-Leu-Gln(Tmob)-Tyr(3-I,Me)-βAla-Glu(Rink-MBHA)-O*p*NB (**10**) (53 mg) following the procedure described above using SPhos as ligand. These conditions also led to the removal of the *p*NB group. Acidolytic cleavage of an aliquot of the resulting resin **11** afforded the biaryl cyclic peptide **12** in 21% purity. t_R = 5.71 min. MS (ESI): *m/z* = 1010.5 $[M + H]^+$.

General method for the synthesis of the biaryl bicyclic peptides 1, 2 and 3

The N-terminal Boc group of the corresponding biaryl cyclic tripeptidyl resin **5**, **8** or **11** was removed by treatment with a solution of TMSOTf and 2,6-lutidine in CH_2Cl_2 (final concentrations: 2 M TMSOTf and 3 M 2,6-lutidine) (3 × 30 min) at room temperature, and the resulting resin was washed with CH_2Cl_2 (5 × 1 min), MeOH (3 × 5 min), and DMF (5 × 1 min) [10]. Removal of the Boc group was monitored by the colorimetric Kaiser test [9]. Then, peptide cyclization was achieved with (ethyl cyano(hydroxyimino)acetate-O^2)-tri-1-pyrrolidinylphosphonium hexafluorophosphate (PyOxim) (5 equiv), Oxyma (5 equiv) and DIPEA (10 equiv) in NMP at room temperature for 24 h. After this time, the resin was washed with NMP (6 × 1 min), CH_2Cl_2 (3 × 1min), and diethyl ether (3 × 1 min). The completion of the cyclization was monitored by the Kaiser test [9]. The resulting biaryl bicyclic peptidyl resin was cleaved with TFA/H_2O/TIS (95:2.5:2.5) whilst being stirred for 2 h at room temperature. Following TFA evaporation and diethyl ether extraction, the crude peptide was dissolved in H_2O/CH_3CN (1:1), lyophilized, and characterized by ESI-MS, tandem mass spectrometry and HRMS.

Biaryl bicyclic peptide 1

The biaryl bicyclic peptide **1** was prepared from biaryl cyclic peptidyl resin **5** following the general procedure described above. Acidolytic cleavage of the resulting resin gave the expected biaryl bicyclic peptide **1**, as confirmed by mass spectrometry. MS (ESI): *m/z* = 914.4 $[M - 18 + H]^+$, 932.4 $[M + H]^+$. HRMS (ESI): calcd. for $C_{45}H_{61}N_{11}O_{11}Na$ $[M + Na]^+$ 954.4444, found 954.4448.

Biaryl bicyclic peptide 2

The biaryl bicyclic peptide **2** was prepared from biaryl cyclic peptidyl resin **8** following the general procedure described above. Acidolytic cleavage of an aliquot of the resulting resin gave the expected biaryl bicyclic peptide **2**, as confirmed by mass spectrometry. MS (ESI): *m/z* = 888.4 $[M - 18 + H]^+$, 910.4 $[M - 18 + Na]^+$. HRMS (ESI): calcd. for $C_{42}H_{53}N_{11}O_{11}Na$ $[M - 18 + Na]^+$ 910.3818, found 910.3804.

Biaryl bicyclic peptide 3

The biaryl bicyclic peptide **3** was prepared from biaryl cyclic peptidyl resin **11** following the general procedure described above. Acidolytic cleavage of the resulting resin gave the expected biaryl bicyclic peptide **3**, as confirmed by mass spectrometry. MS (ESI): *m/z* = 974.5 $[M - 18 + H]^+$, 992.5 $[M + H]^+$. HRMS (ESI): calcd. for $C_{47}H_{63}N_{11}O_{12}Na$ $[M - 18 + Na]^+$ 996.4550, found 996.4558.

References

1. Lei, H.; Stoakes, M. S.; Herath, K. P. B.; Lee, J.; Schwabacher, A. W. *J. Org. Chem.* **1994,** *59*, 4206-4210.
2. Gu, W.; Liu, S.; Silverman, R. B. *Org. Lett.* **2002**, *4*, 4171-4174.
3. Feng, Z.; Min, Q.-Q.; Xiao, Y.-L.; Zhang, B.; Zhang, X. *Angew. Chem. Int. Ed.* **2014**, *53*, 1669-1673.
4. Bednarz, M.; De Paul, S.; Kanamar-Lapudi, R.; Perlberg, A.; Zhang, H. Patent WO2009042733A1
5. Byk, G.; Cohen-Ohana, M.; Raichman, D. *Pept. Sci.* **2006**, *84*, 274-282.
6. Romanovskis, P.; Spatola, A. F. *J. Peptide Res*. **1998**, *52*, 356-374.
7. Cerezo, V.; Amblard, M.; Martinez, J.; Verdié, P.; Planas, M.; Feliu, L. *Tetrahedron* **2008**, *64*, 10538-10545.
8. Liu, N.-N.; Zhao, S.-M.; Zhao, J.-F.; Zeng, G.-Z.; Tan, N.-H.; Liu, J.-P. *Tetrahedron* **2014**, *70*, 6630-6640.
9. Kaiser, E.; Colescott, R. L.; Bossinger, C. D.; Cook, P. *Anal. Biochem.* **1970**, *34*, 595–598.
10. Zhang, A. J.; Russell, D. H.; Zhu, J. P.; Burguess, K. *Tetrahedron Lett.* **1998**, *39*, 7439-7442

Copies of HPLC, MS and NMR spectra

Amino acids

Boc-Phe(4-I)-OMe

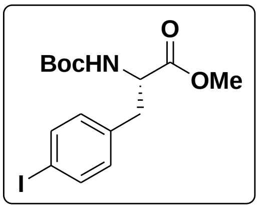

HPLC (λ = 220 nm)

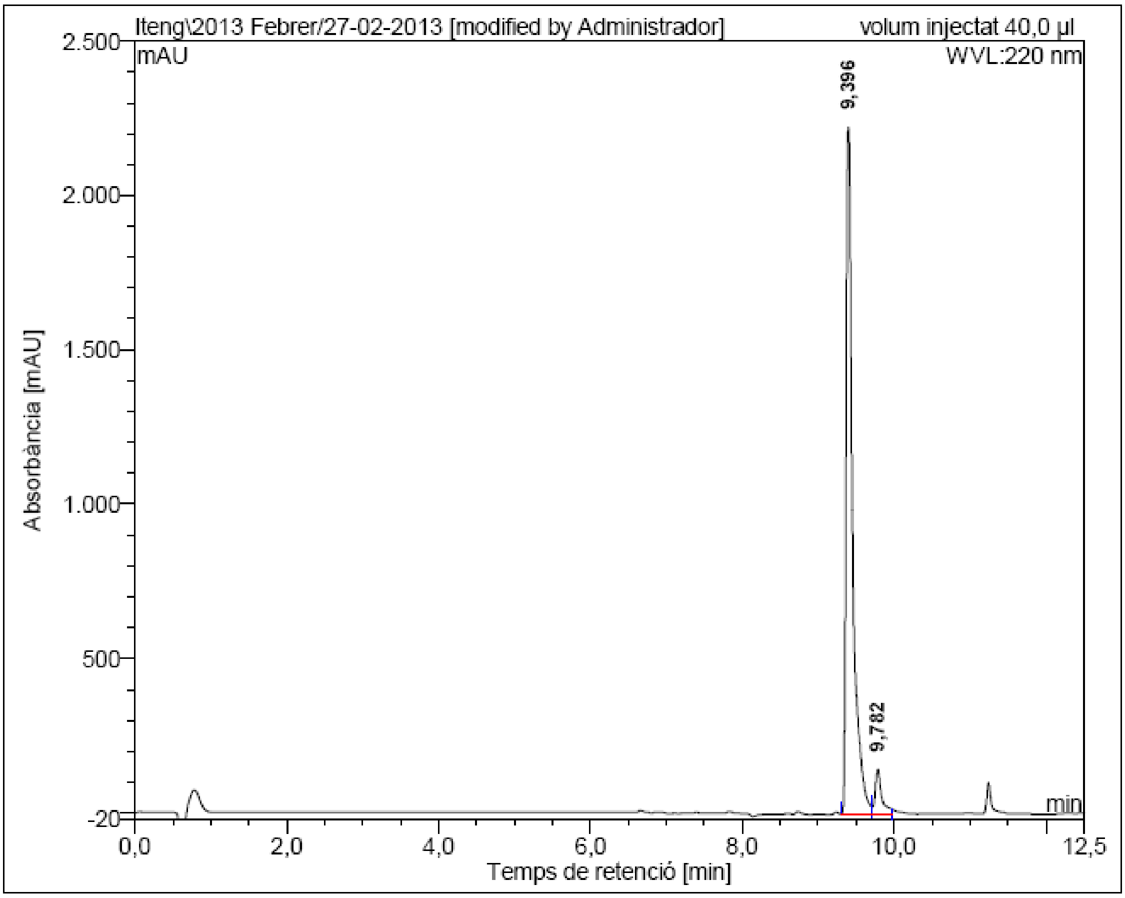

No.	Temps retenció min	alçada mAU	Area mAU*min	Area relativa %
1	9,40	2224,195	224,479	93,96
2	9,78	145,582	14,440	6,04
Total:		2369,777	238,918	100,00

ESI-MS *m/z* (%)

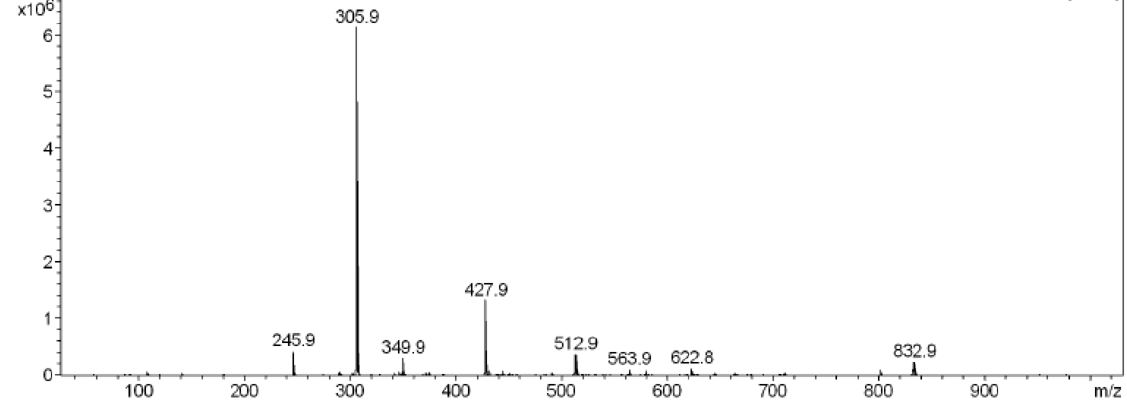

^{1}H NMR (400 MHz, $CDCl_3$) δ (ppm)

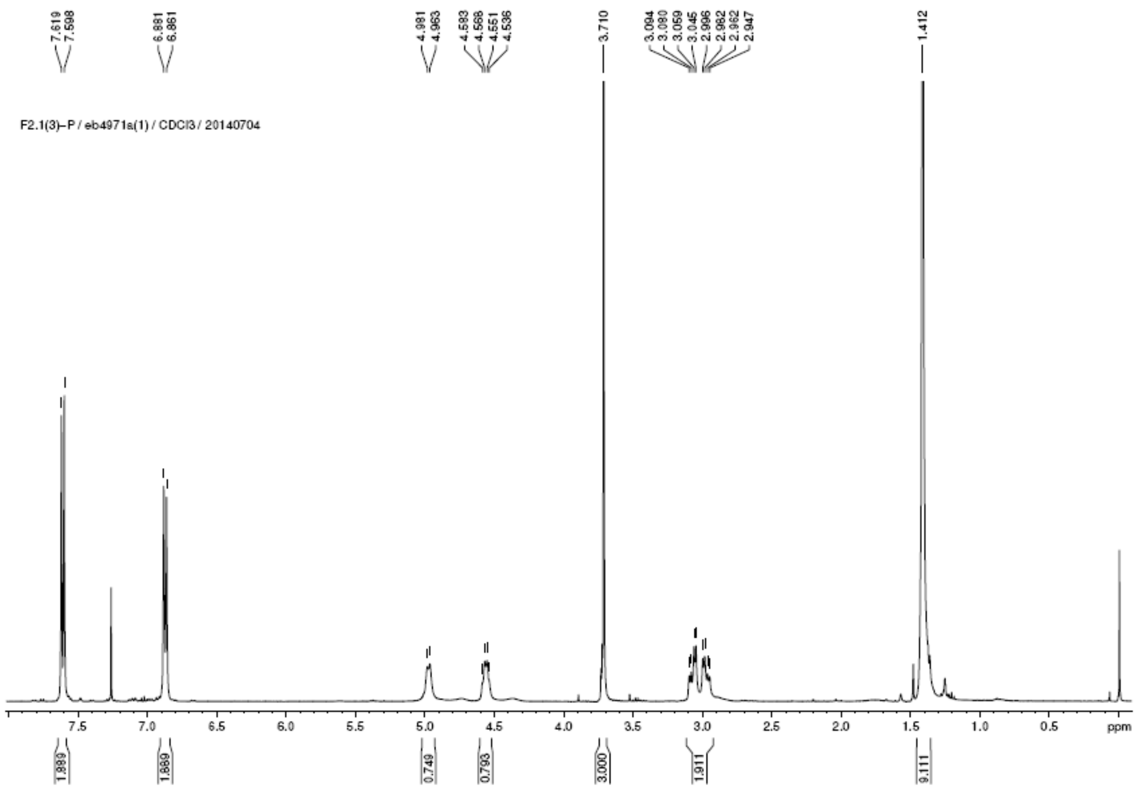

^{13}C NMR (75 MHz, $CDCl_3$) δ (ppm)

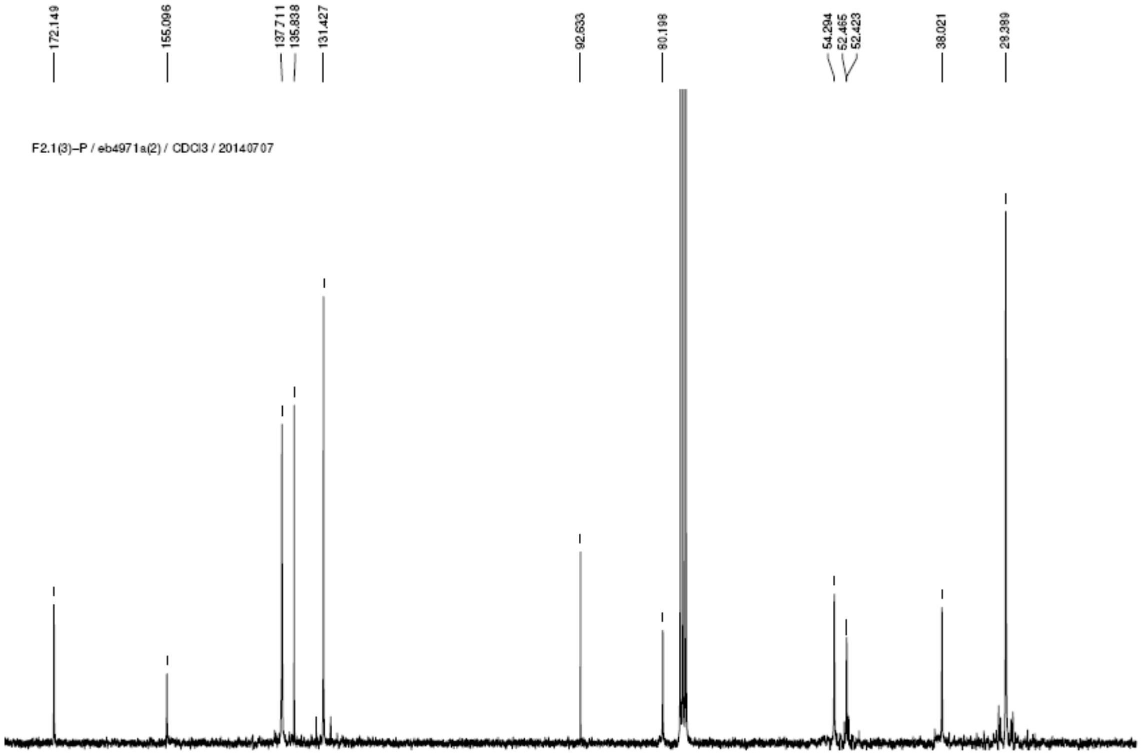

Boc-Phe(4-BPin)-OMe

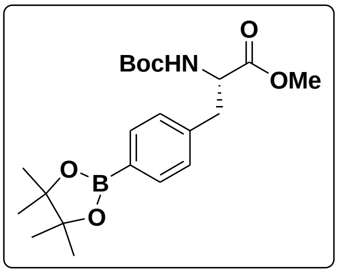

HPLC (λ = 220 nm)

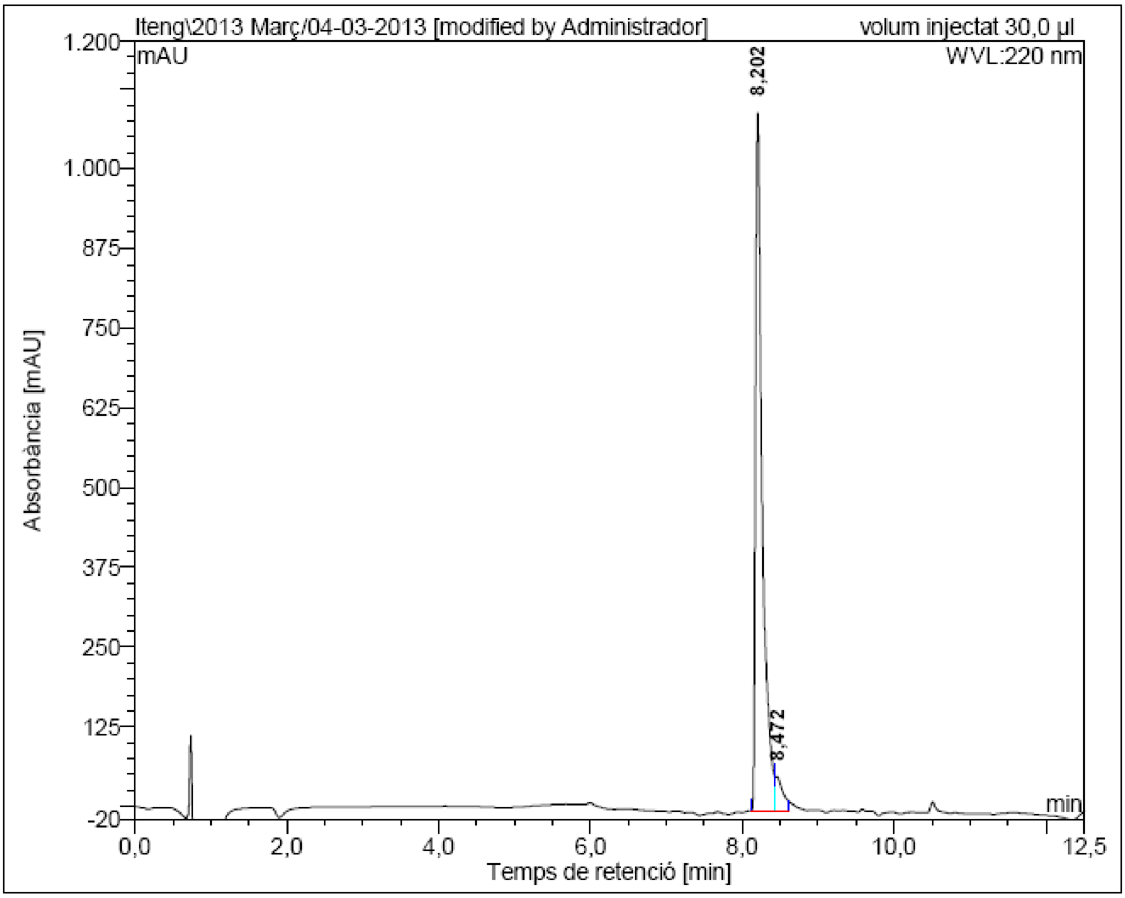

No.	Temps retenció min	alçada mAU	Area mAU*min	Area relativa %
1	8,20	1094,402	112,593	94,14
2	8,47	52,426	7,012	5,86
Total:		1146,827	119,605	100,00

ESI-MS *m/z* (%)

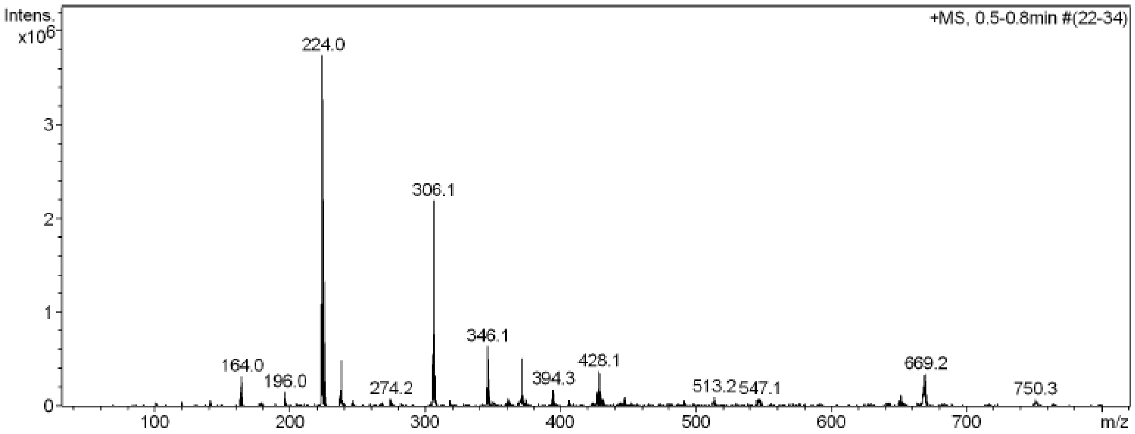

^{1}H NMR (400 MHz, $CDCl_3$) δ (ppm)

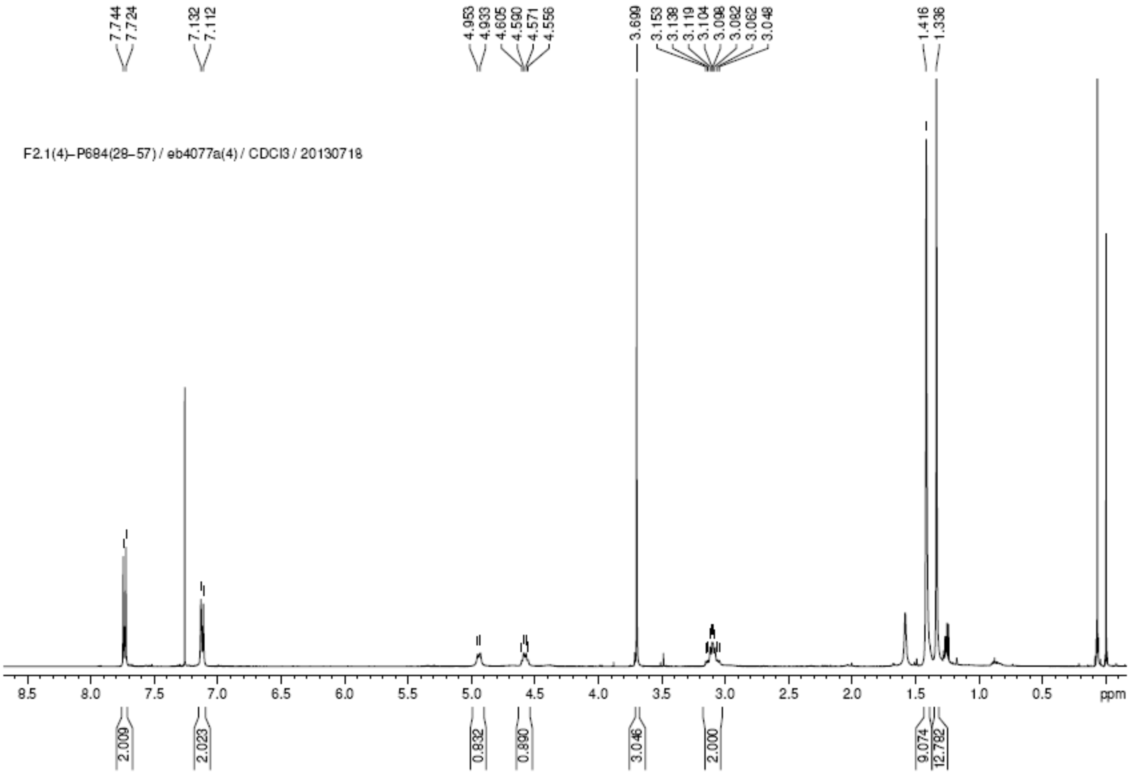

^{13}C NMR (75 MHz, $CDCl_3$) δ (ppm)

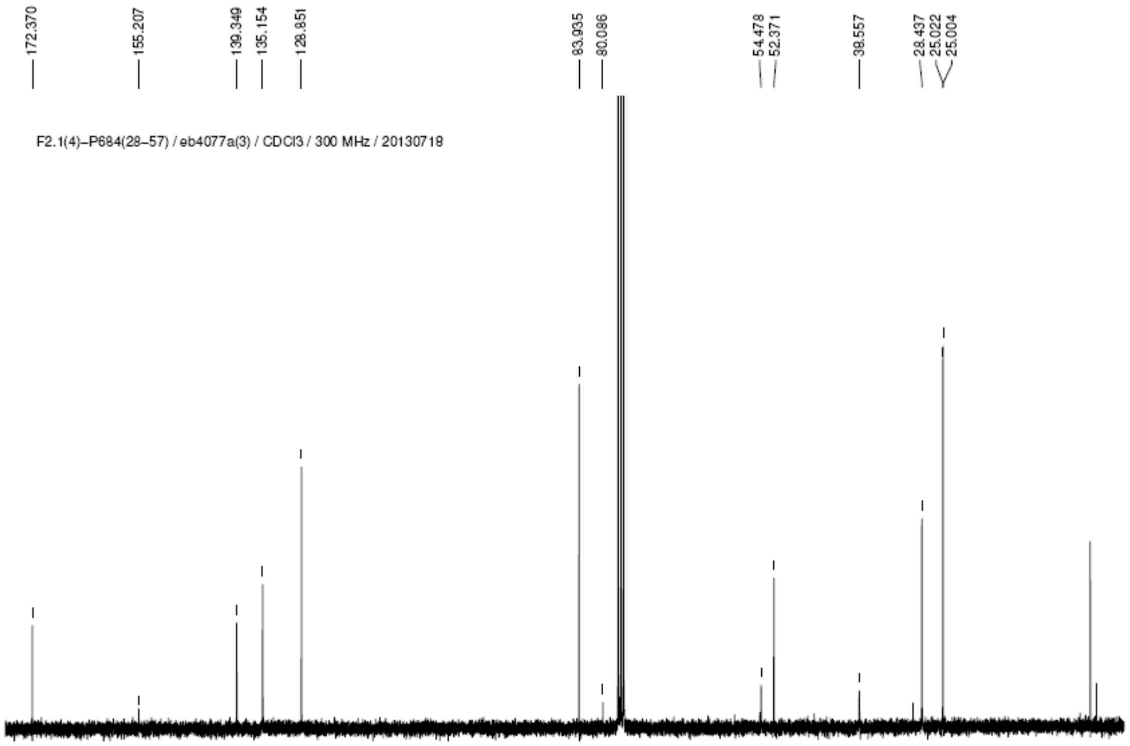

Boc-Phe(4-BPin)-OH

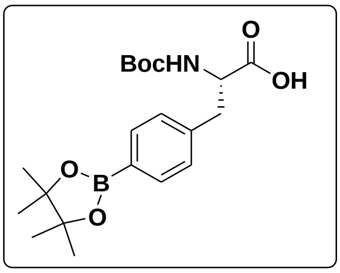

HPLC (λ = 220 nm)

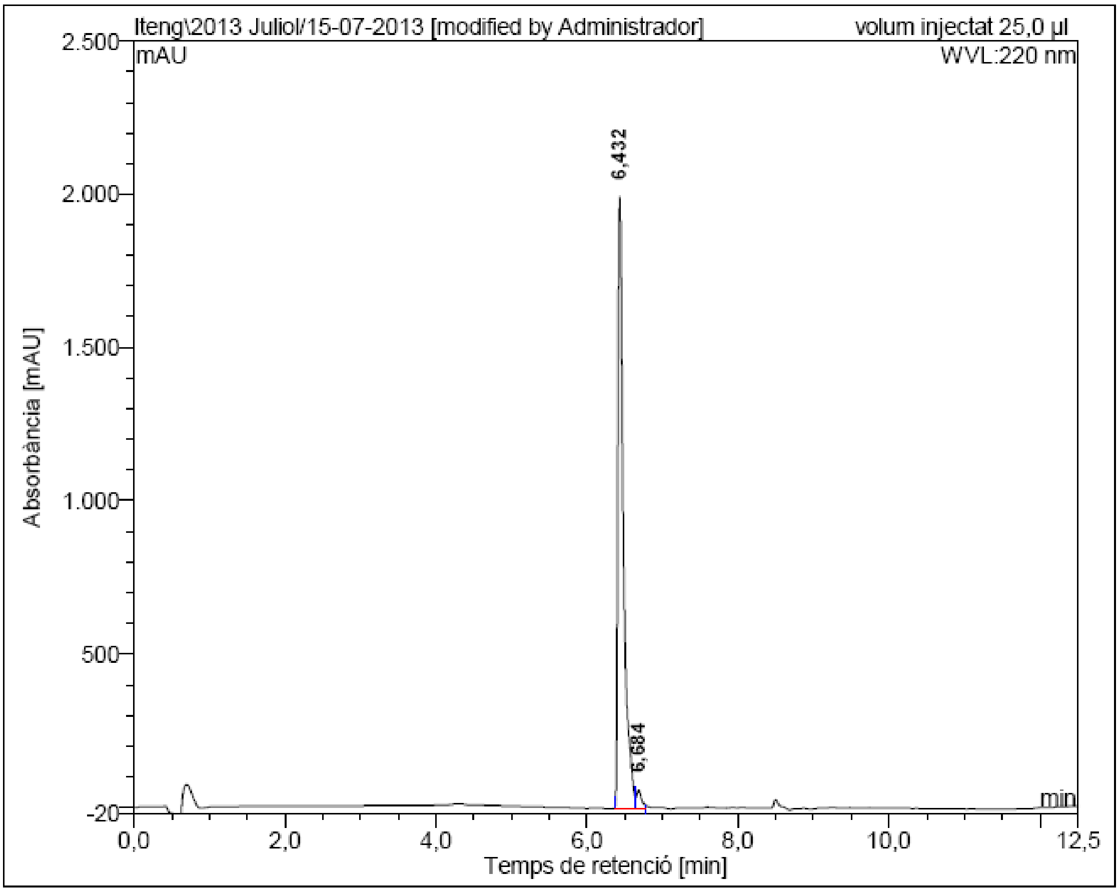

No.	Temps retenció min	alçada mAU	Area mAU*min	Area relativa %
1	6,43	1996,373	172,752	97,52
2	6,68	60,412	4,393	2,48
Total:		2056,784	177,145	100,00

ESI-MS *m/z* (%)

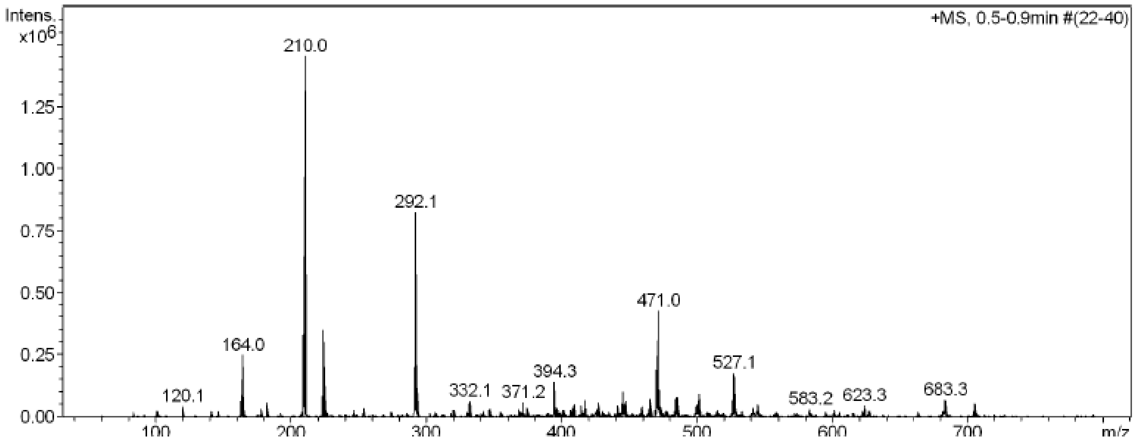

^{1}H NMR (400 MHz, $CDCl_3$) δ (ppm)

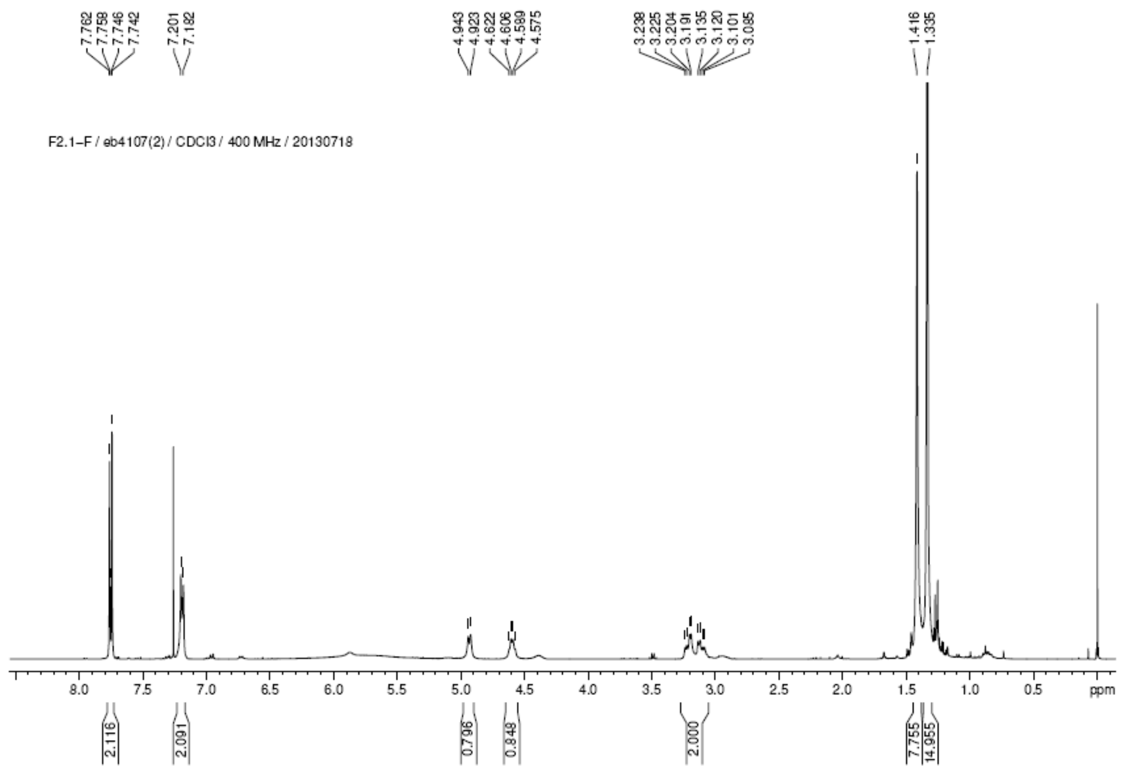

^{13}C NMR (75 MHz, $CDCl_3$) δ (ppm)

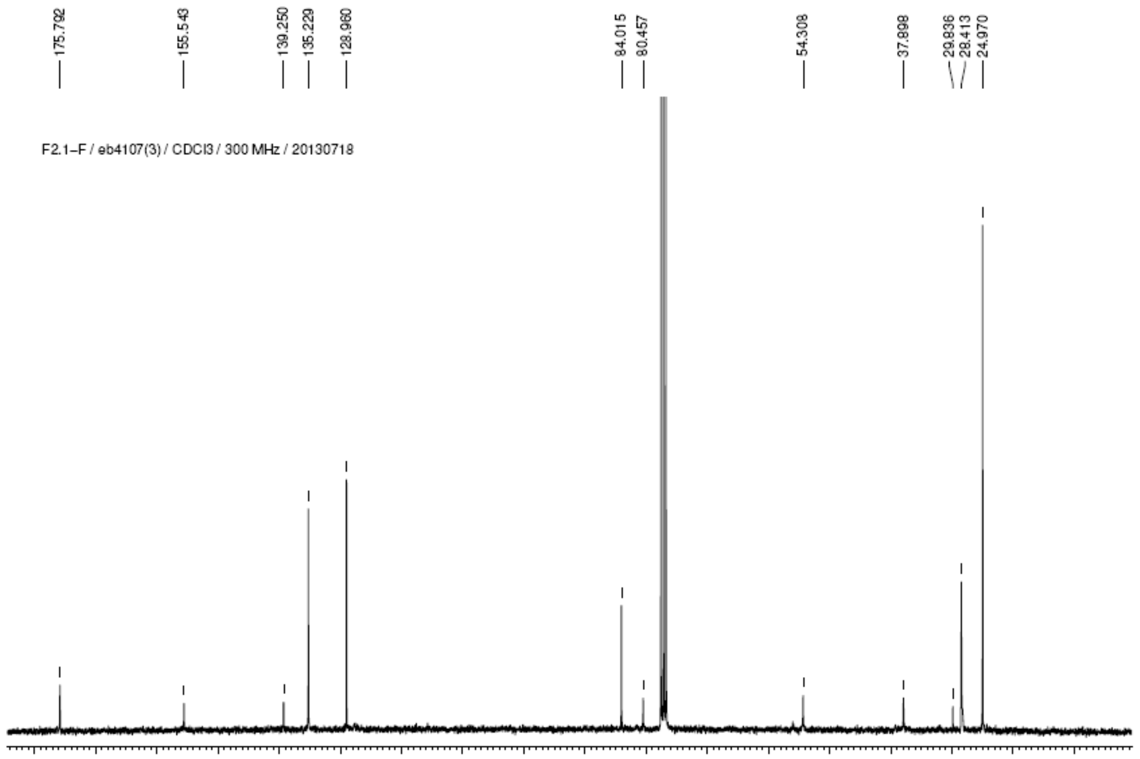

Fmoc-Phe(4-I)-OH

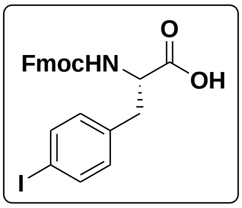

HPLC (λ = 220 nm)

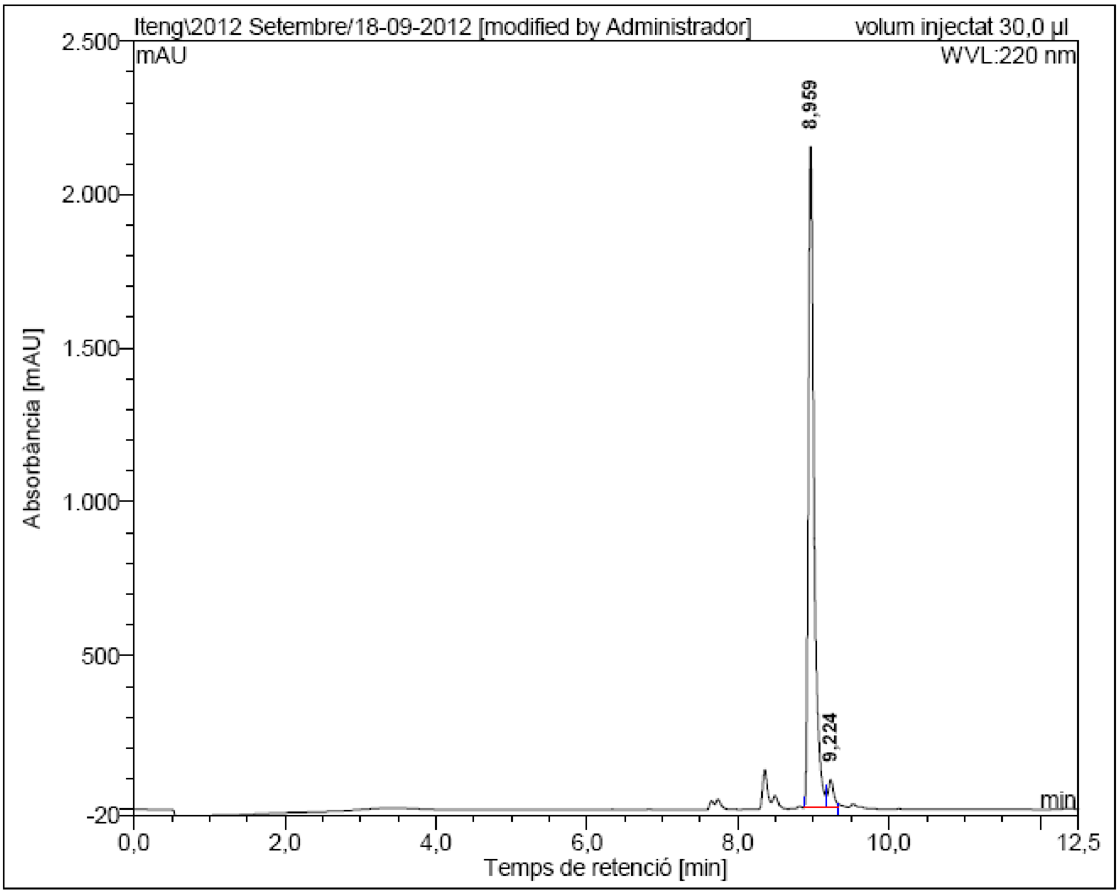

No.	Temps retenció min	alçada mAU	Area mAU*min	Area relativa %
1	8,96	2152,020	190,045	95,93
2	9,22	92,483	8,055	4,07
Total:		2244,503	198,099	100,00

ESI-MS *m/z* (%)

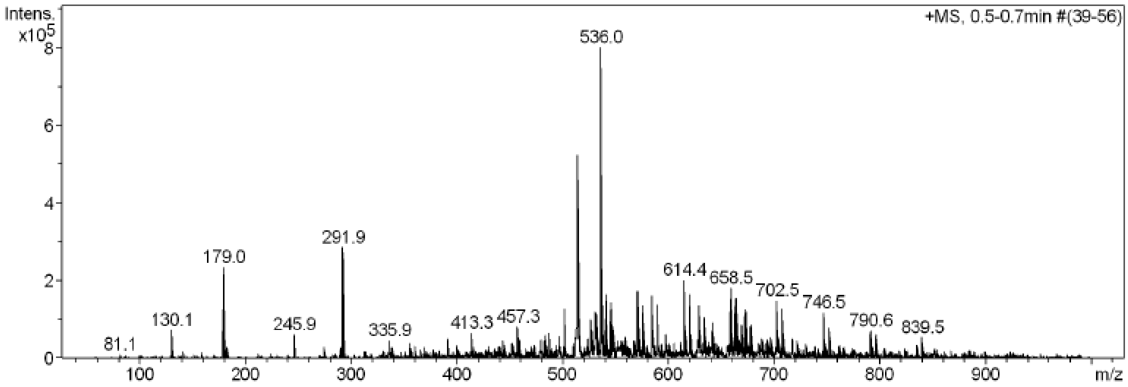

^{1}H NMR (400 MHz, DMSO-d_6) δ (ppm)

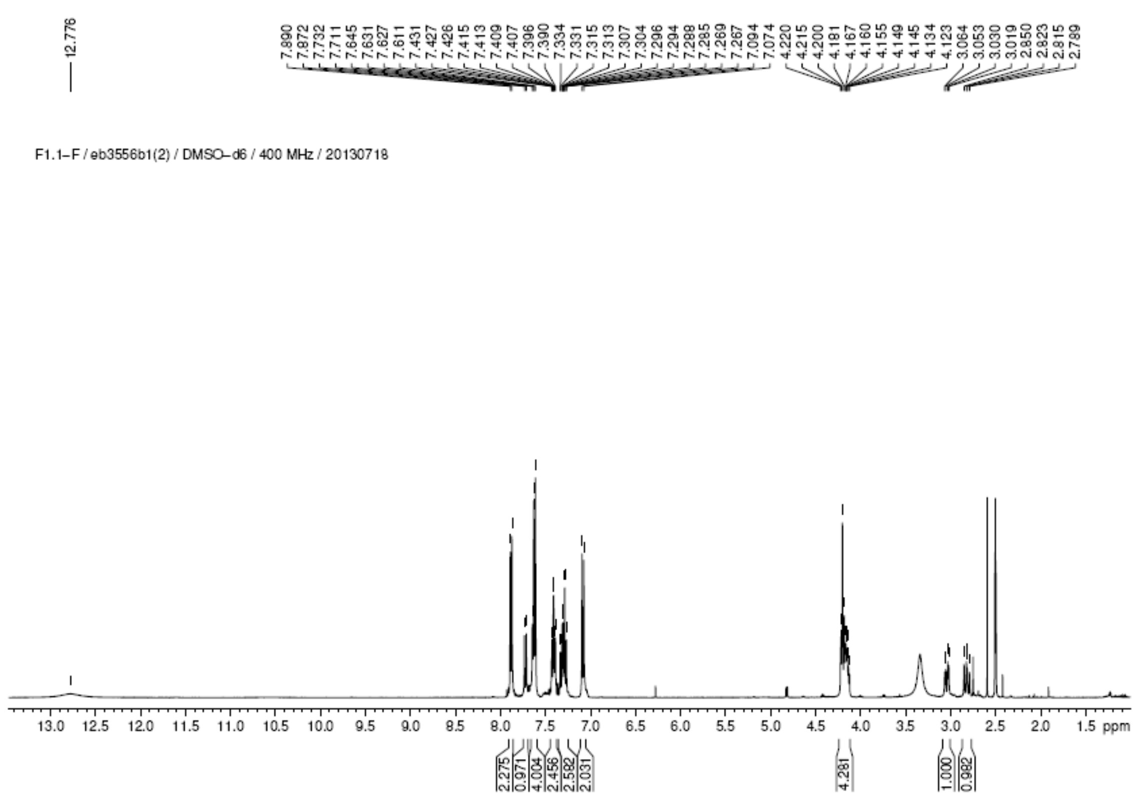

^{13}C NMR (75 MHz, DMSO-d_6) δ (ppm)

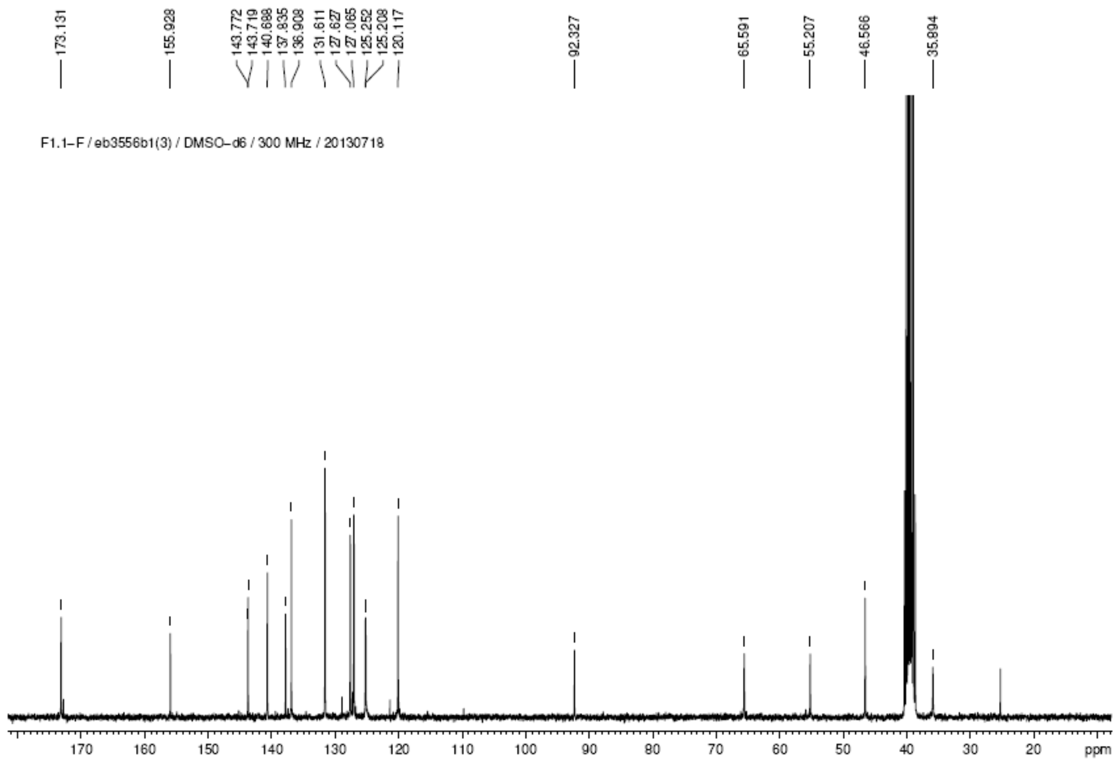

Fmoc-Glu(O*t*-Bu)-O*p*NB

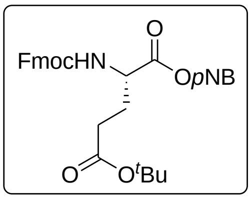

HPLC (λ = 220 nm)

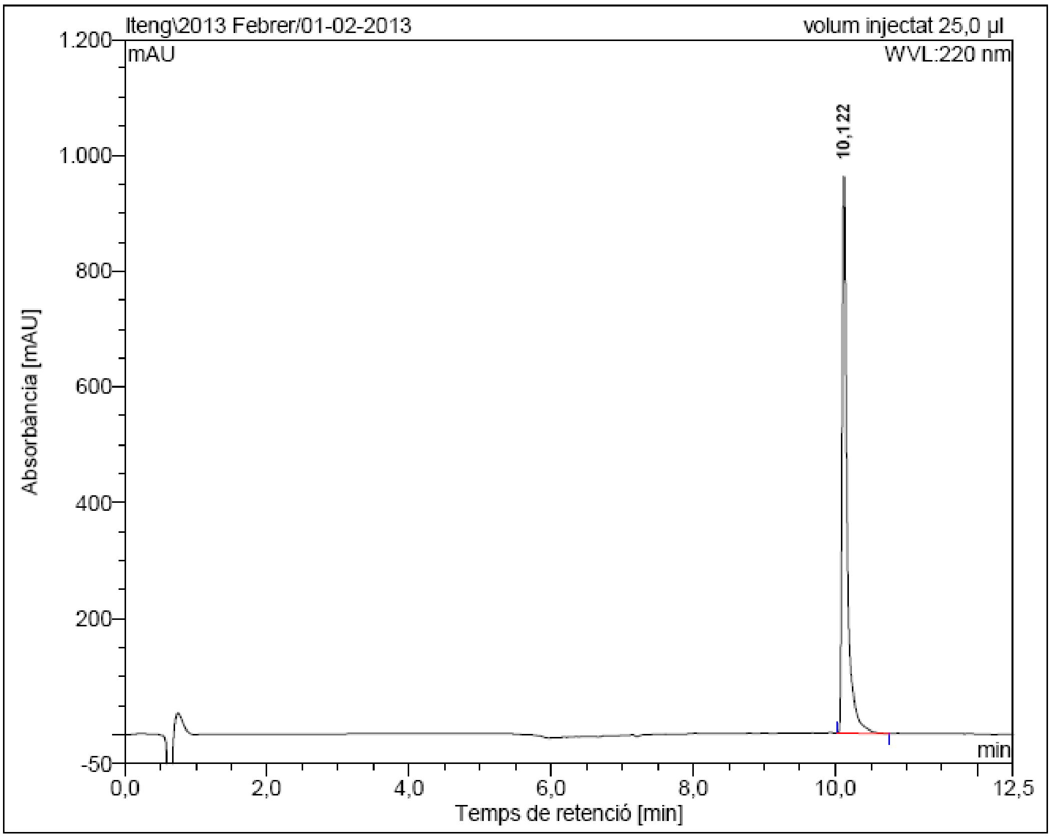

No.	Ret.Time (detected) min	Height mAU	Area mAU*min	Rel.Area %
1	10,12	960,063	76,164	100,00
Total:		960,063	76,164	100,00

^1H NMR (400 MHz, $CDCl_3$) δ (ppm)

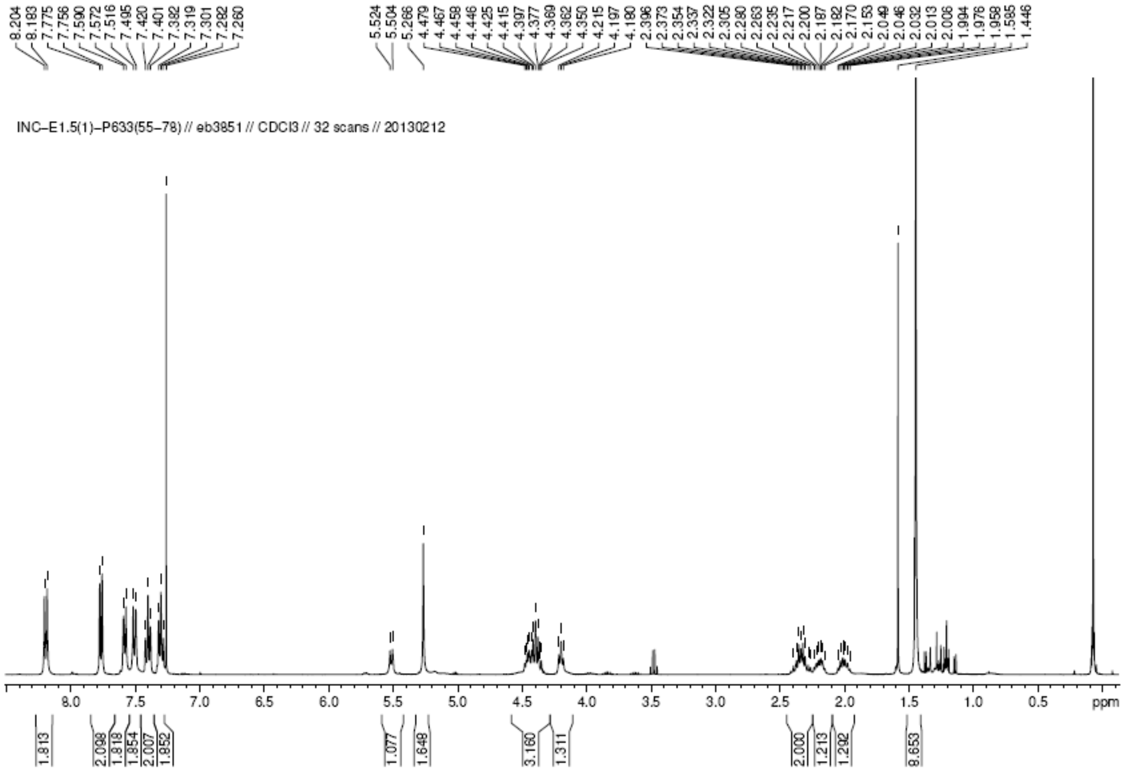

Fmoc-Glu-O*p*NB

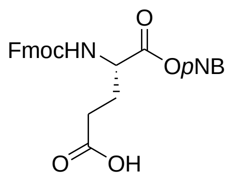

7

HPLC (λ = 220 nm)

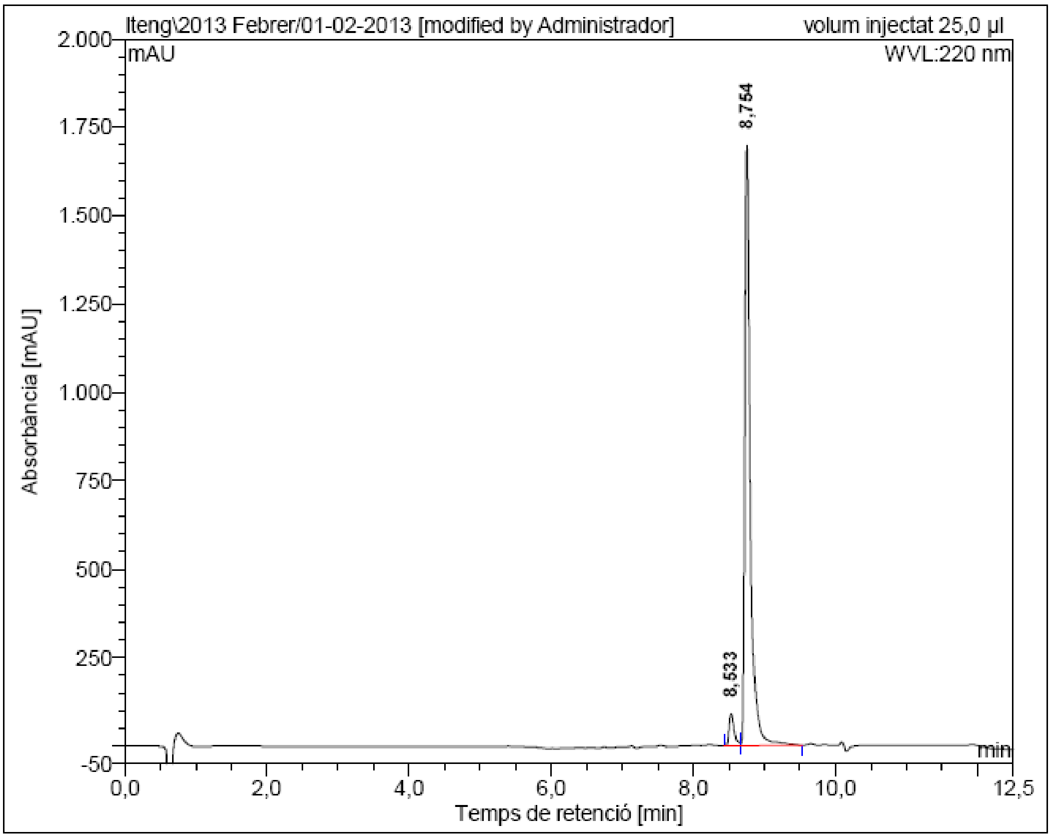

No.	Ret.Time (detected) min	Height mAU	Area mAU*min	Rel.Area %
1	8,53	90,891	7,731	4,81
2	8,75	1698,022	152,928	95,19
Total:		1788,913	160,659	100,00

^{1}H NMR (400 MHz, $CDCl_3$) δ (ppm)

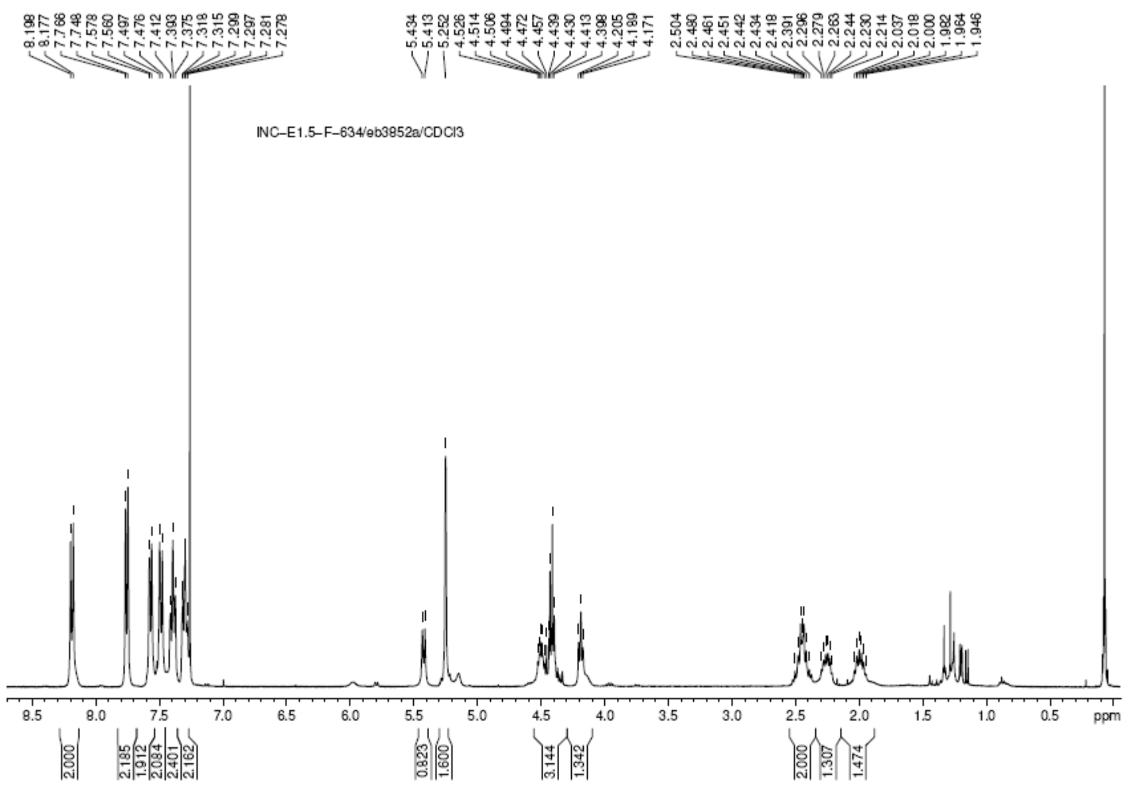

Boc-Tyr(3-B(OH)$_2$,Me)-OMe

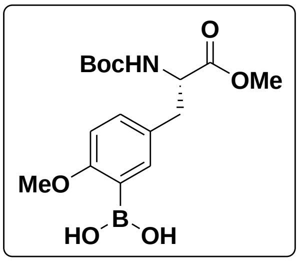

HPLC (λ = 220 nm)

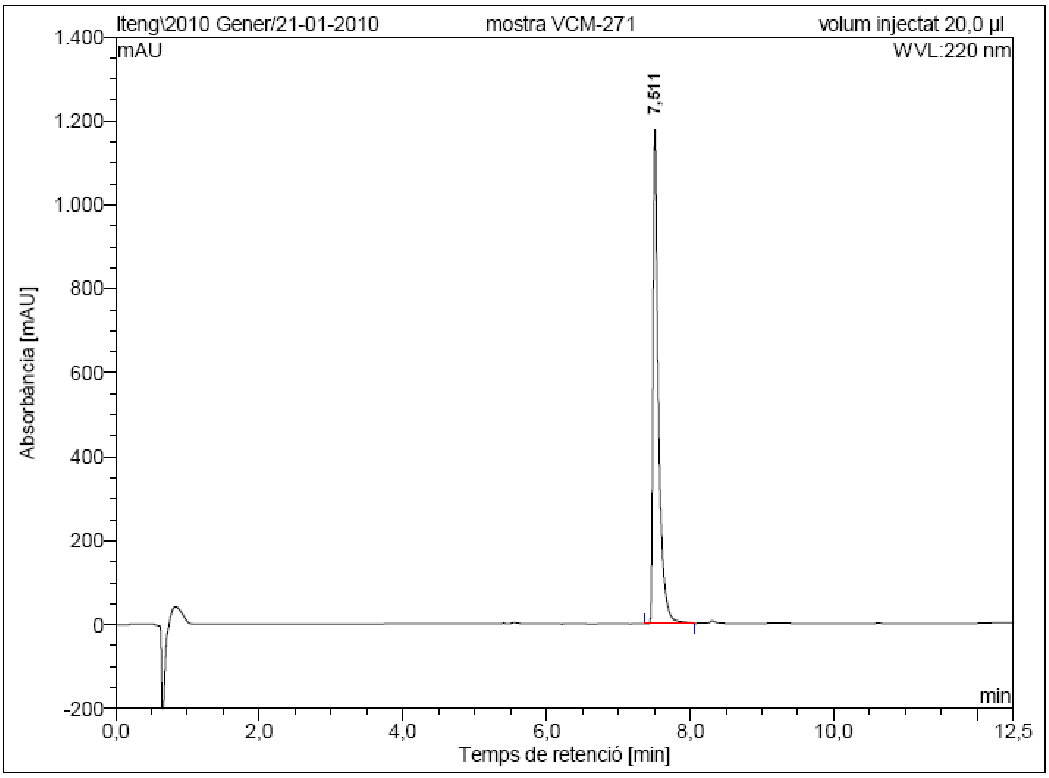

No.	Temps retenció min	alçada mAU	Area mAU*min	Area relativa %
1	7,51	1175,909	105,036	100,00
Total:		1175,909	105,036	100,00

^{1}H NMR (400 MHz, $CDCl_3$)

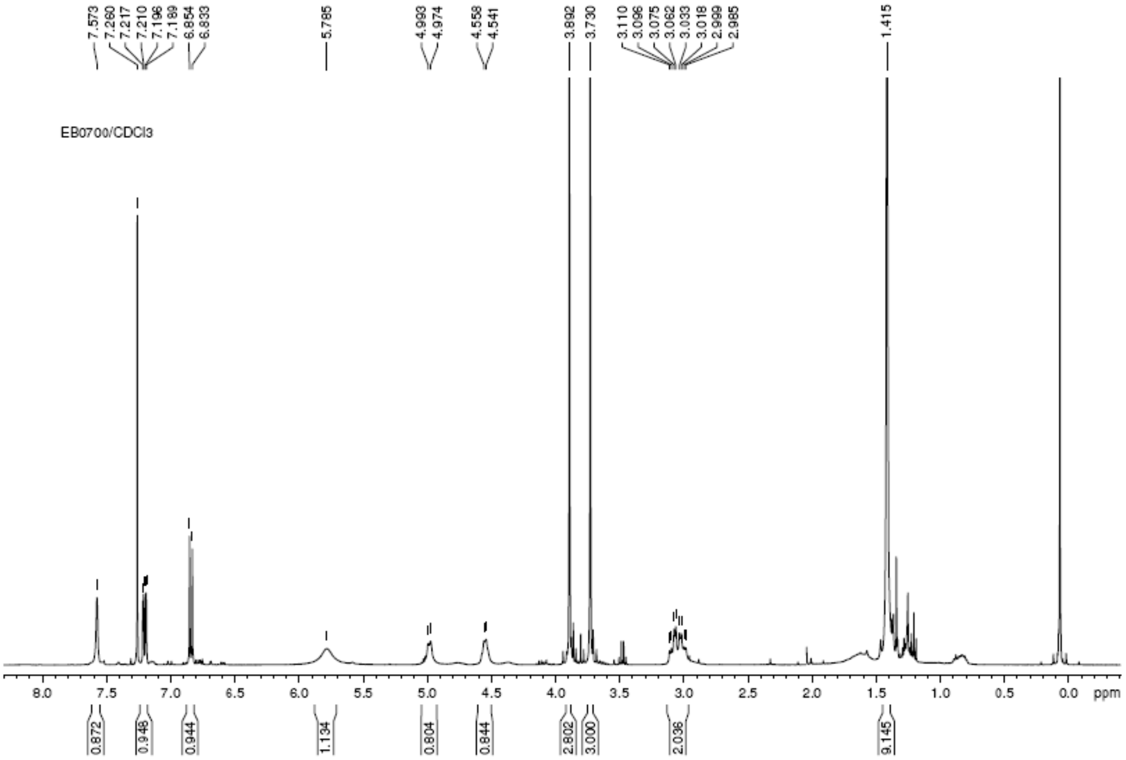

^{13}C NMR (75 MHz, $CDCl_3$)

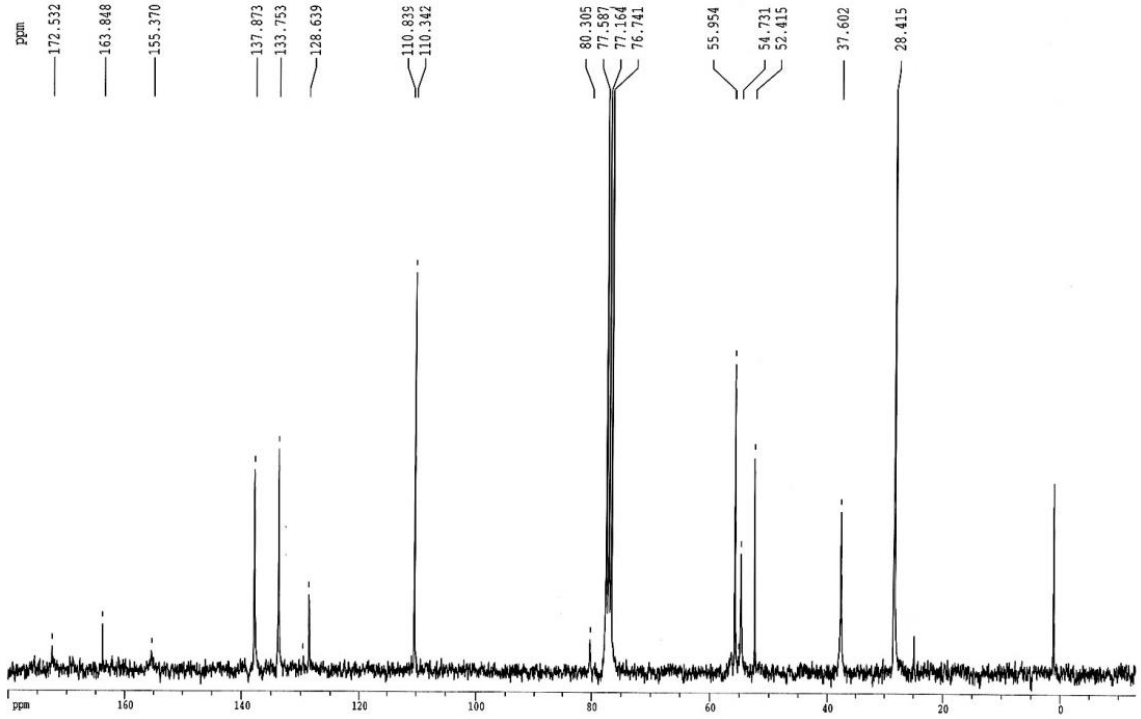

Boc-Tyr(3-B(OH)$_2$,Me)-OH

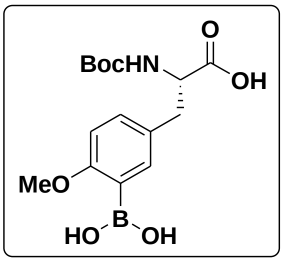

HPLC (λ = 220 nm)

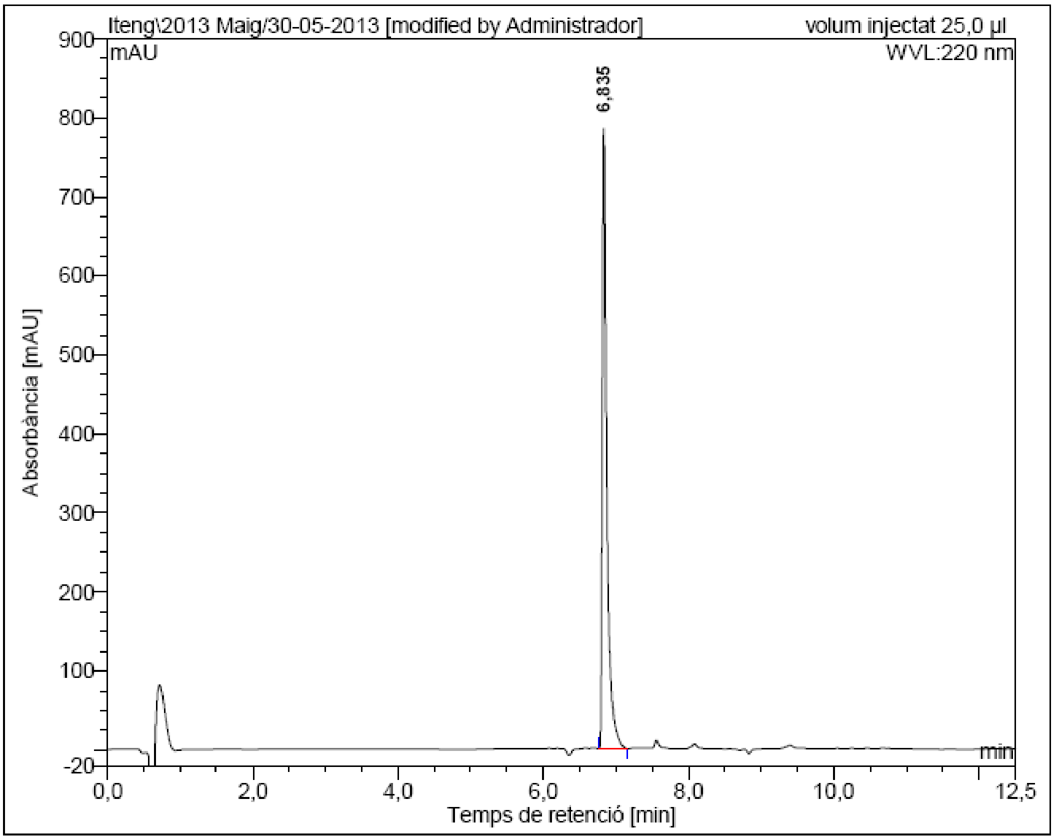

No.	Temps retenció min	alçada mAU	Area mAU*min	Area relativa %
1	6,84	785,715	55,818	100,00
Total:		785,715	55,818	100,00

^{1}H NMR (400 MHz, $CDCl_3$)

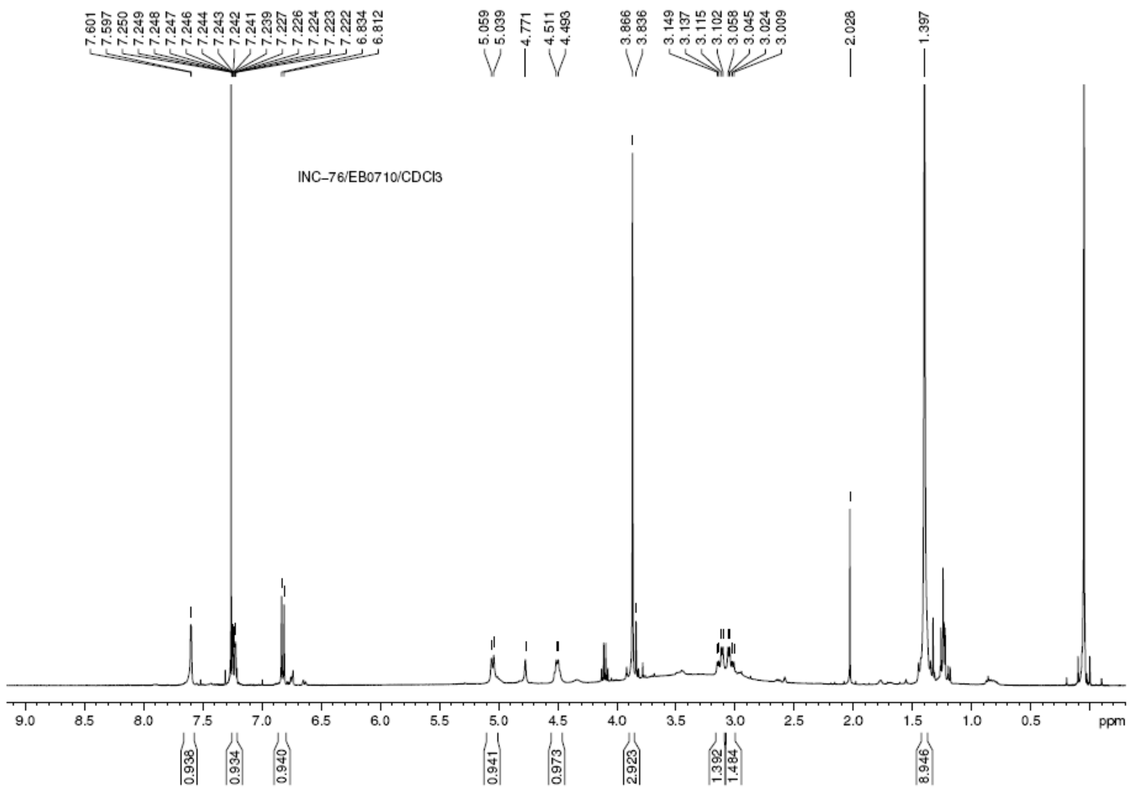

^{13}C NMR (75 MHz, $CDCl_3$)

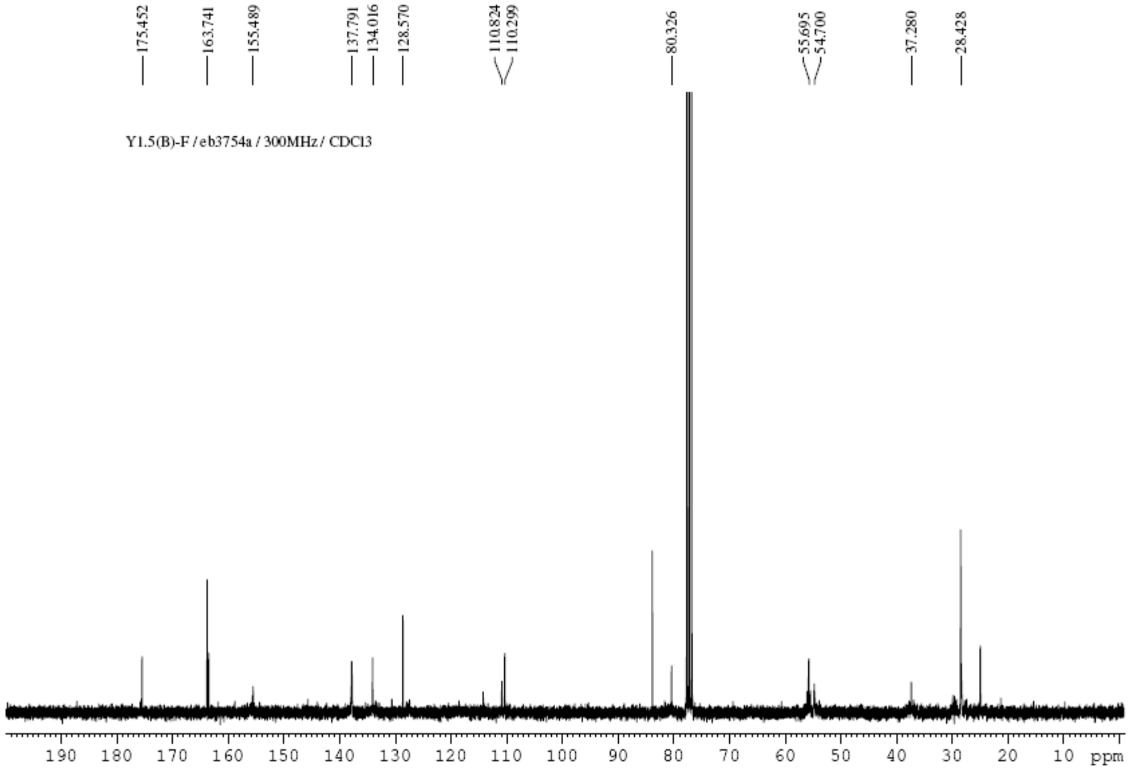

H-Tyr(3-I,Me)-OMe

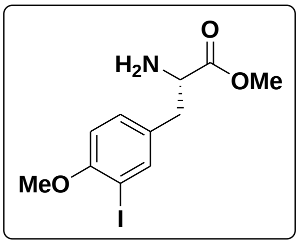

HPLC (λ = 220 nm)

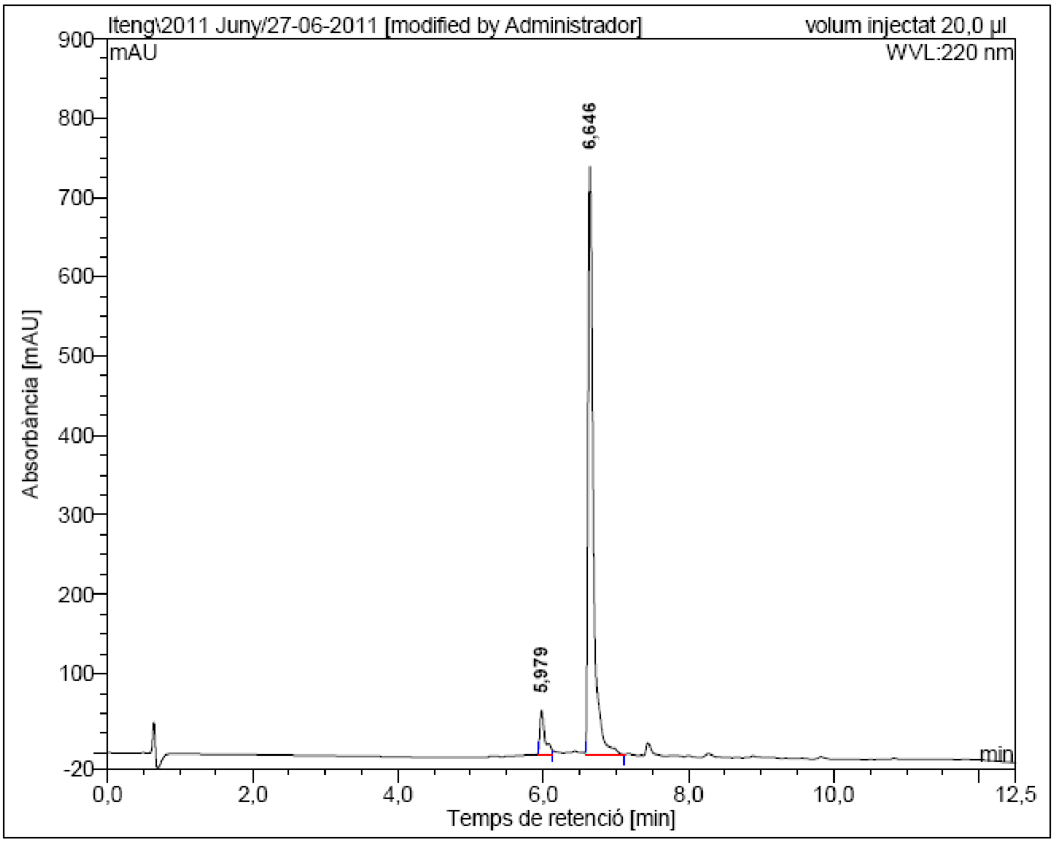

No.	Temps retenció min	alçada mAU	Area mAU*min	Area relativa %
1	5,98	55,426	4,665	7,15
2	6,65	740,599	60,541	92,85
Total:		796,025	65,206	100,00

^{1}H NMR (400 MHz, $CDCl_3$)

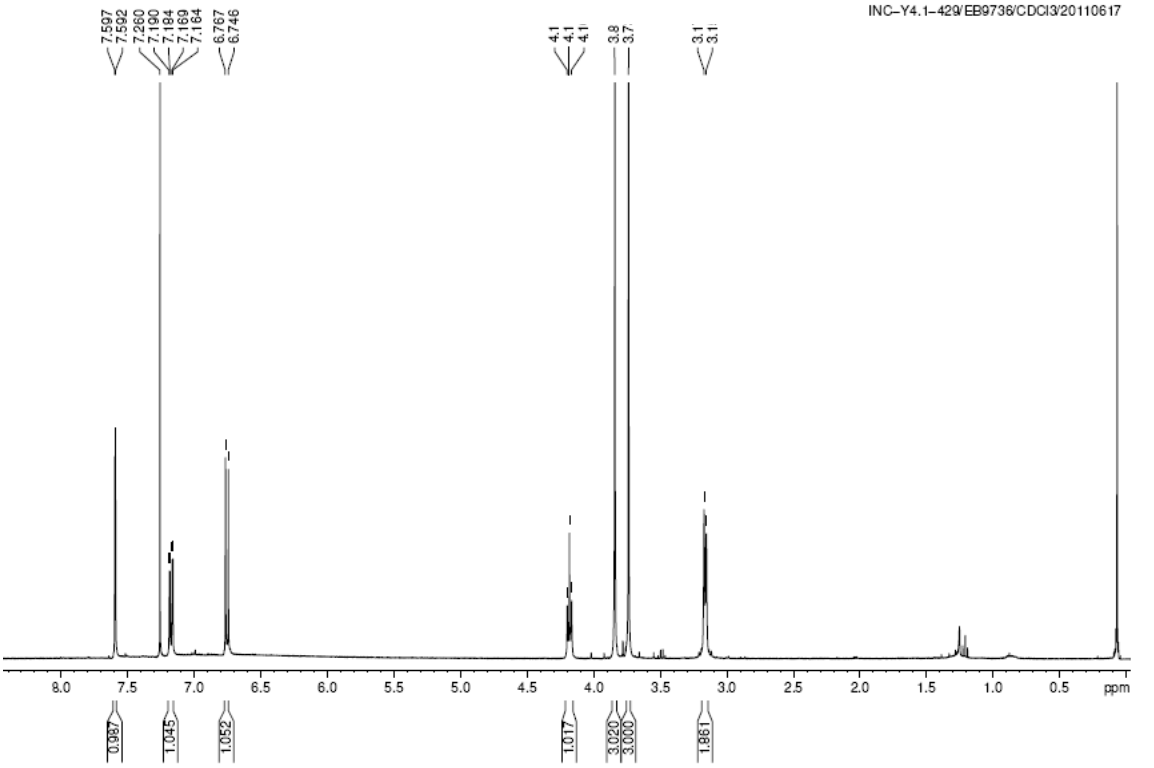

^{13}C NMR (75 MHz, DMSO-d_6)

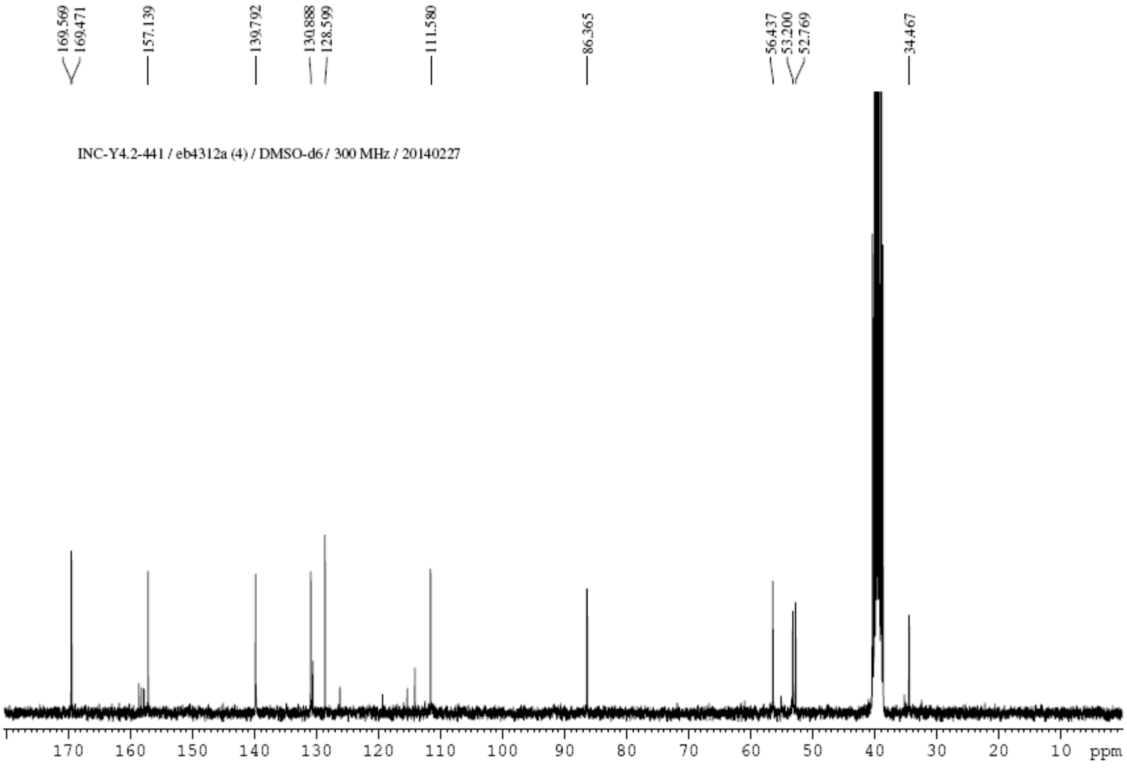

H-Tyr(3-I,Me)-OH

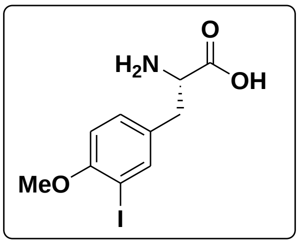

HPLC (λ = 220 nm)

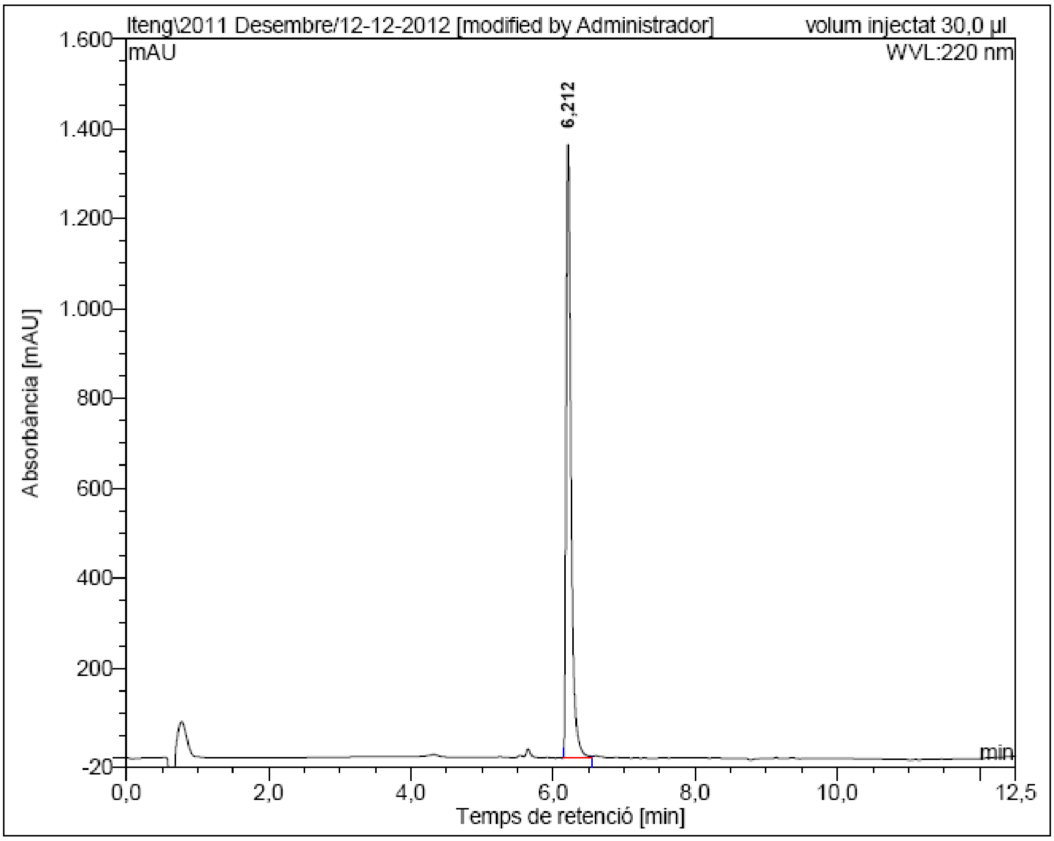

No.	Temps retenció min	alçada mAU	Area mAU*min	Area relativa %
1	6,21	1365,180	107,408	100,00
Total:		1365,180	107,408	100,00

^{1}H NMR (300 MHz, DMSO-d_6)

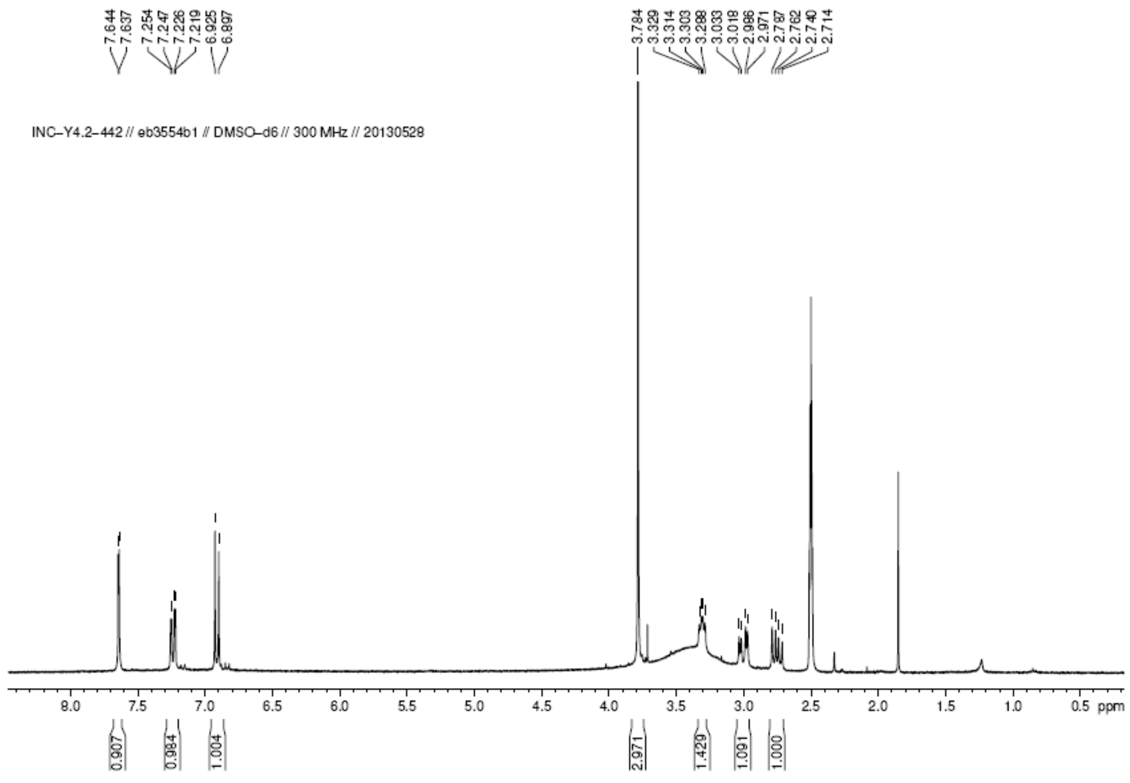

^{13}C NMR (75 MHz, DMSO-d_6)

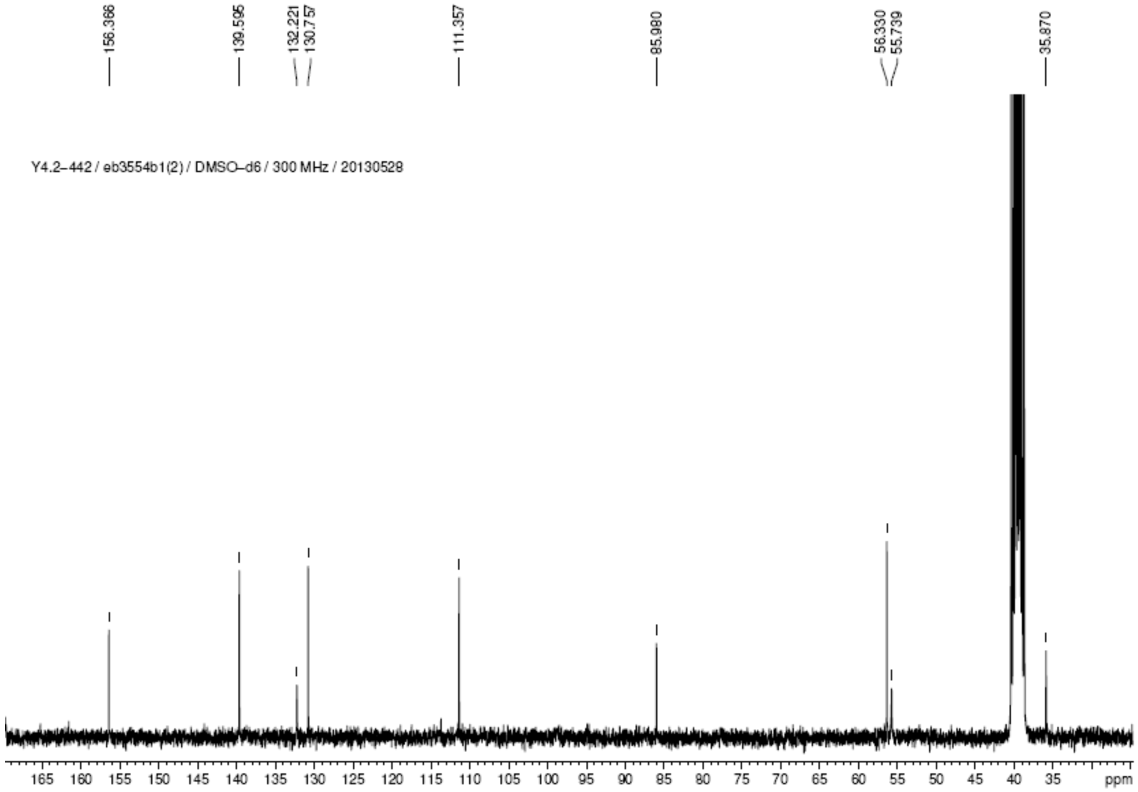

Fmoc-Tyr(3-I,Me)-OH

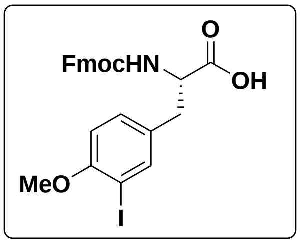

HPLC (λ = 220 nm)

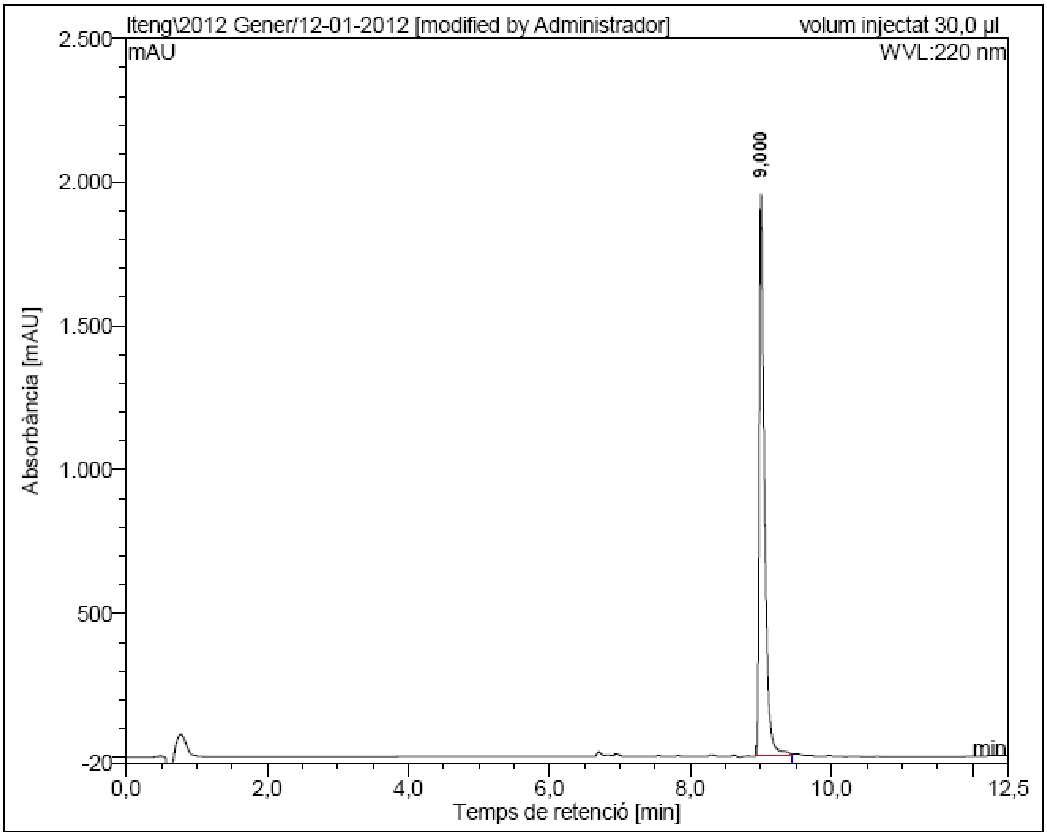

No.	Temps retenció min	alçada mAU	Area mAU*min	Area relativa %
1	9,00	1954,245	167,666	100,00
Total:		1954,245	167,666	100,00

^{1}H NMR (400 MHz, DMSO-d_6)

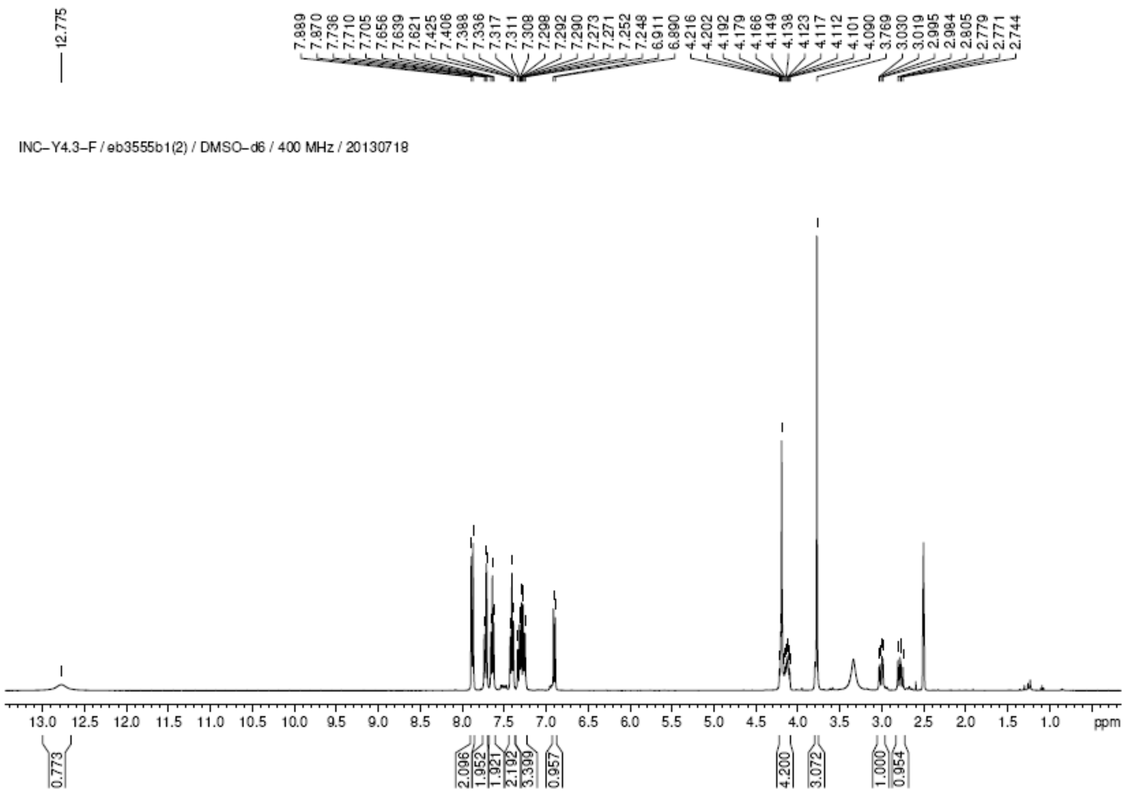

^{13}C NMR (75 MHz, DMSO-d_6)

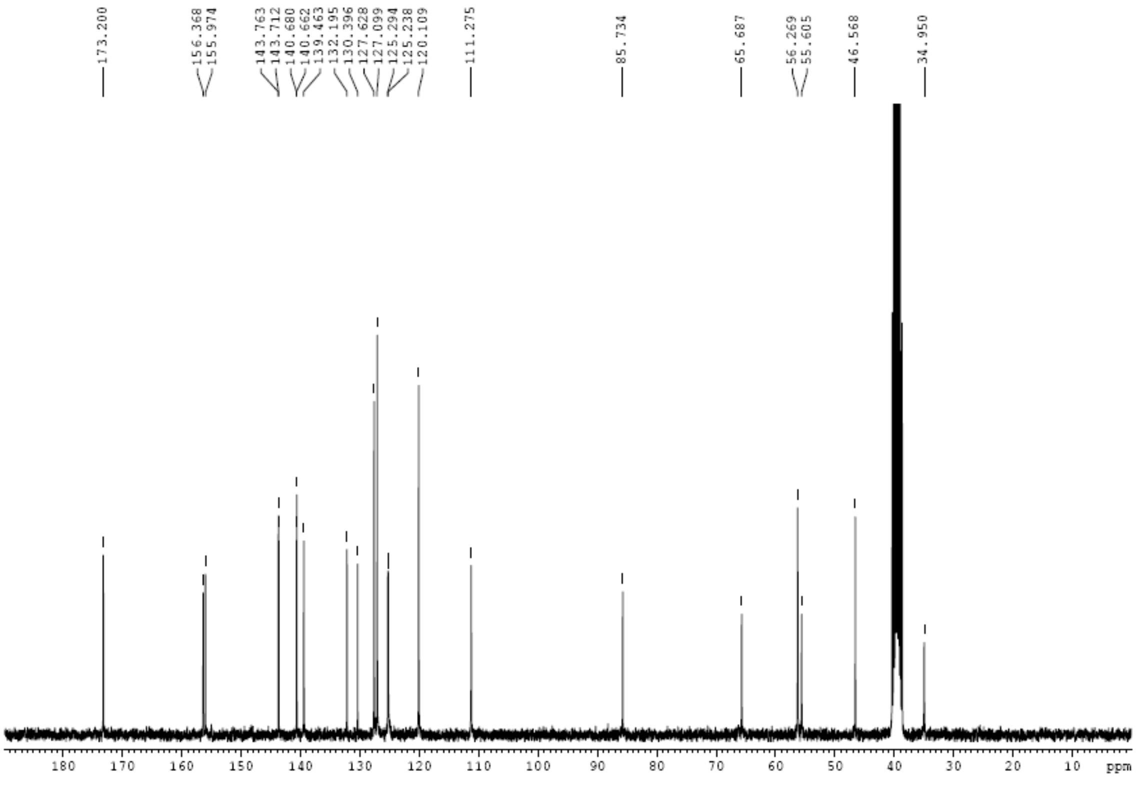

Linear peptides

H-Phe(4-B(OH)$_2$)-Ala-Gln-Leu-Gln-Phe(4-I)-βAla-Gln-O*p*NB

HPLC (λ = 220 nm)

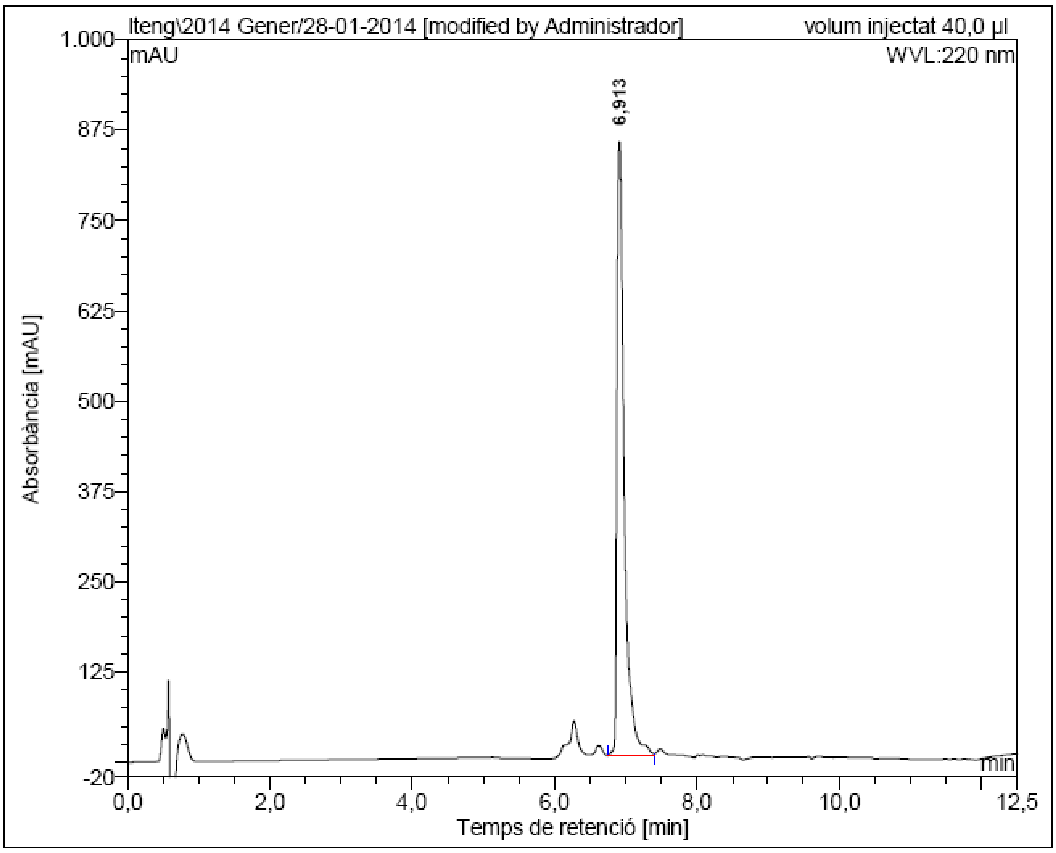

No.	mps retenc min	alçada mAU	Area mAU*min	Area relativa %
1	6,91	849,530	101,466	100,00
Total:		849,530	101,466	100,00

ESI-MS *m/z*

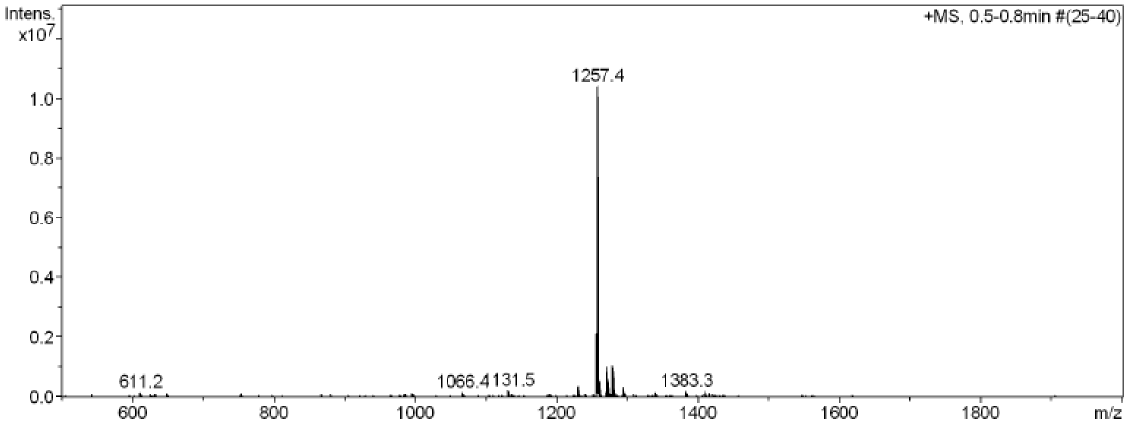

H-Tyr(3-B(OH)$_2$,Me)-Ala-Gln-Gly-Gln-Phe(4-I)-βAla-Gln-OpNB

HPLC (λ = 220 nm)

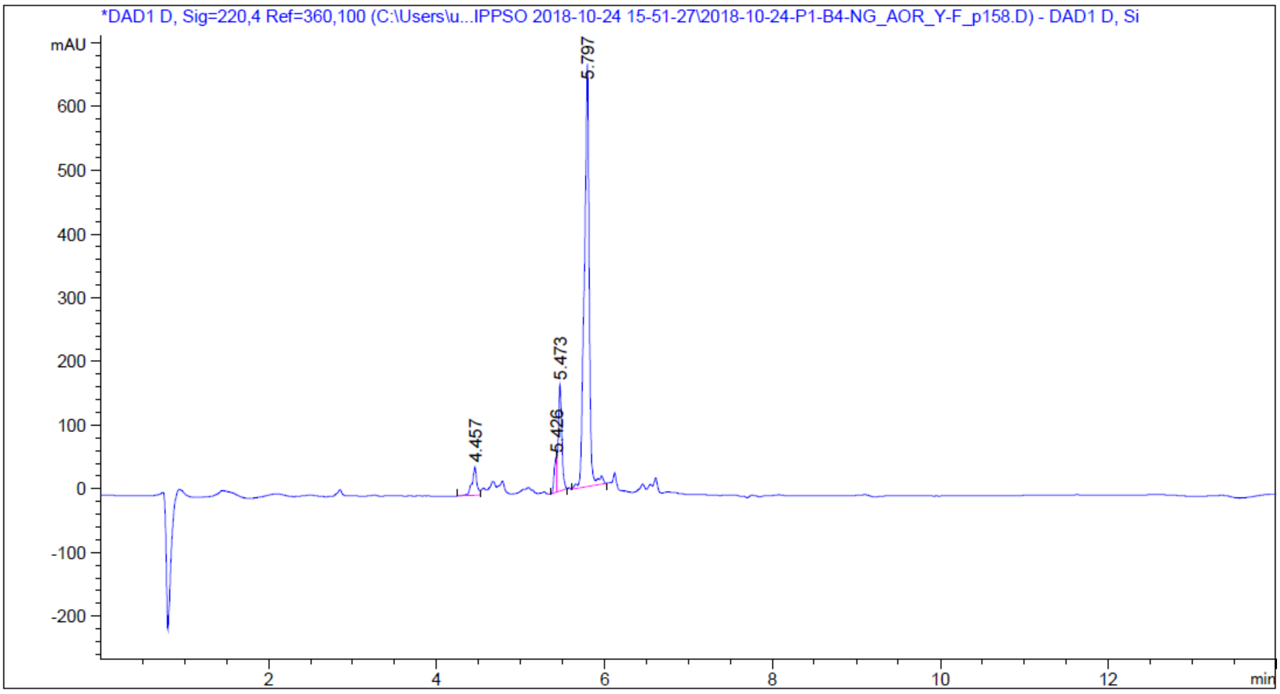

Peak #	RetTime [min]	Type	Width [min]	Area [mAU*s]	Height [mAU]	Area %
1	4.457	BV	0.0552	177.26559	43.80036	5.1372
2	5.426	BV	0.0305	106.40372	55.19851	3.0836
3	5.473	VB	0.0434	492.88232	166.92143	14.2839
4	5.797	VV R	0.0559	2674.06958	664.50629	77.4953

Totals : 3450.62122 930.42659

ESI-MS *m/z*

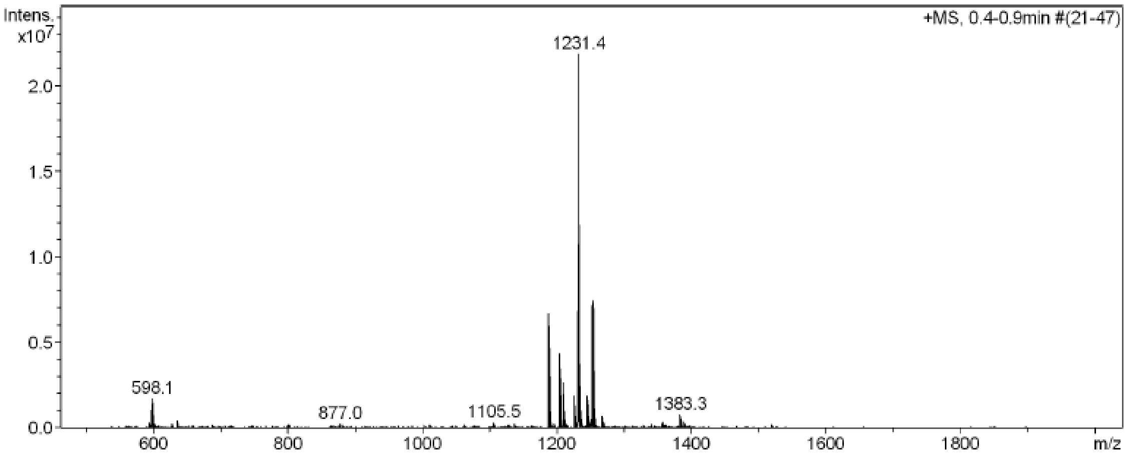

Intens.
x10^7
2.0
1.5
1.0
0.5
0.0
+MS, 0.4-0.9min #(21-47)
1231.4
598.1
877.0
1105.5
1383.3
600
800
1000
1200
1400
1600
1800
m/z

H-Tyr(3-B(OH)$_2$,Me)-Ala-Gln-Leu-Gln-Tyr(3-I,Me)-βAla-Gln-O*p*NB

HPLC (λ = 220 nm)

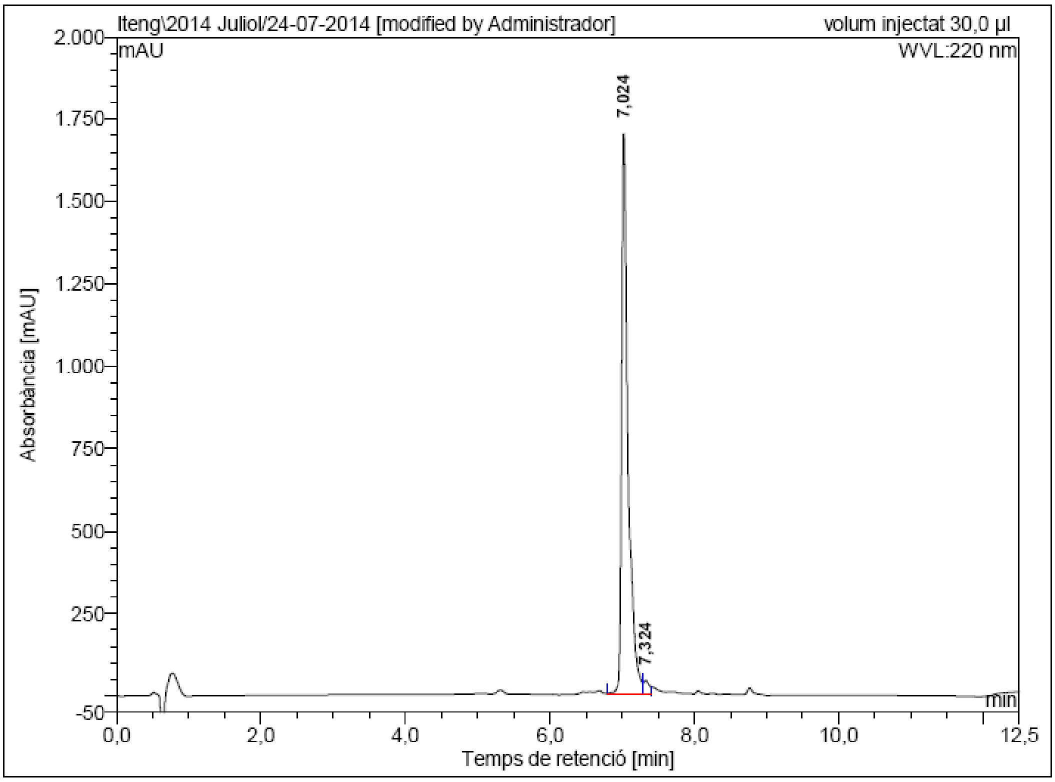

No.	Temps retenció min	alçada mAU	Area mAU*min	Area relativa %
1	7,02	1698,450	172,454	97,99
2	7,32	40,394	3,533	2,01
Total:		1738,844	175,987	100,00

ESI-MS *m/z*

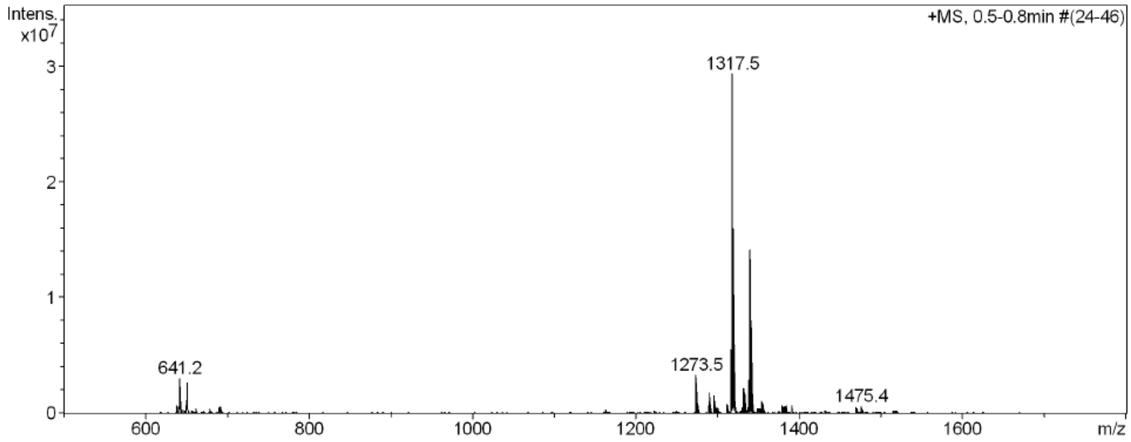

Biaryl cyclic peptide 6

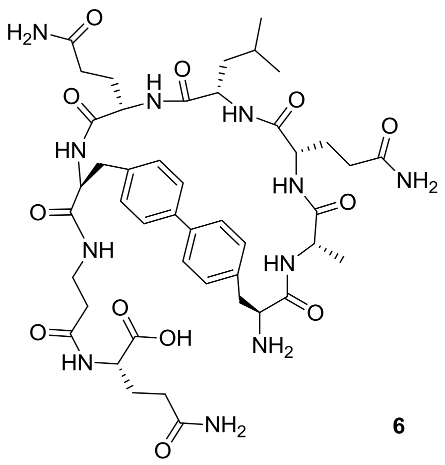

6

HPLC (λ = 220 nm)

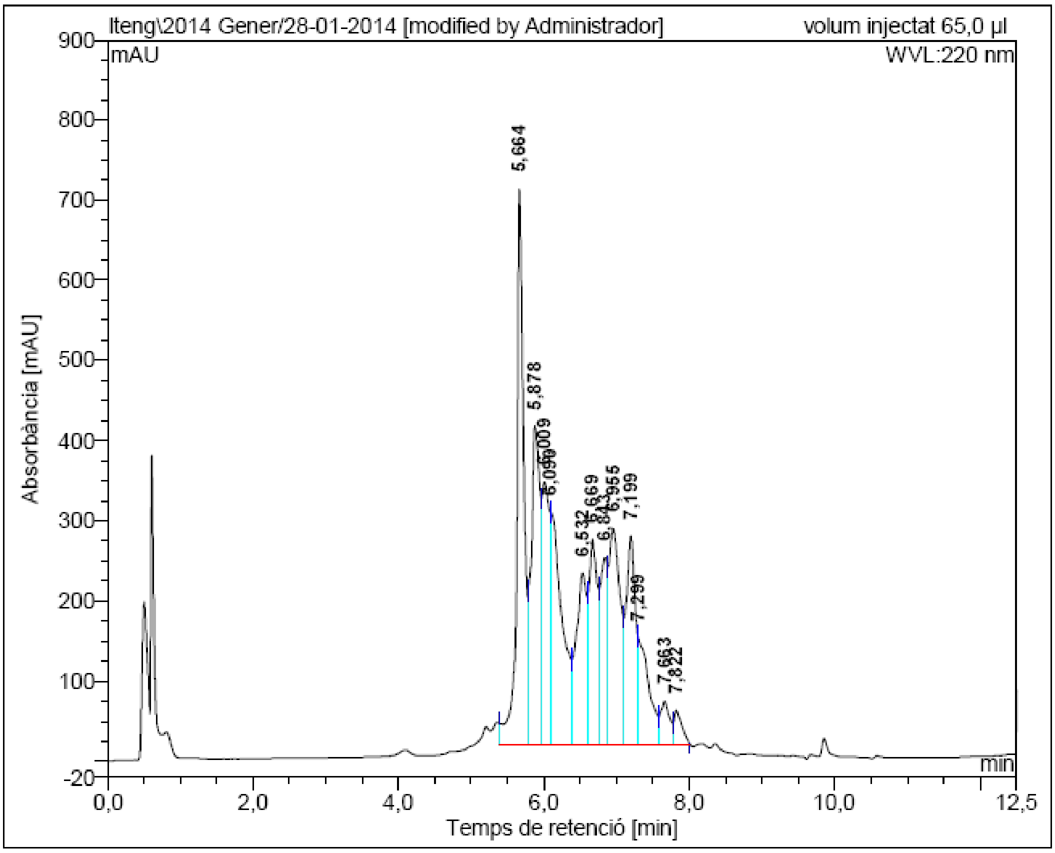

No.	mps retenc min	alçada mAU	Area mAU*min	Area relativa %
1	5,66	693,919	80,158	17,73
2	5,88	398,048	55,909	12,36
3	6,01	328,041	37,187	8,22
4	6,09	290,723	55,853	12,35
5	6,53	214,240	38,462	8,50
6	6,67	257,148	31,678	7,00
7	6,84	234,025	28,814	6,37
8	6,95	269,532	47,536	10,51
9	7,20	260,849	39,585	8,75
10	7,30	134,234	23,338	5,16
11	7,66	54,812	8,059	1,78
12	7,82	43,314	5,649	1,25
Total:		3178,885	452,228	100,00

ESI-MS *m/z*

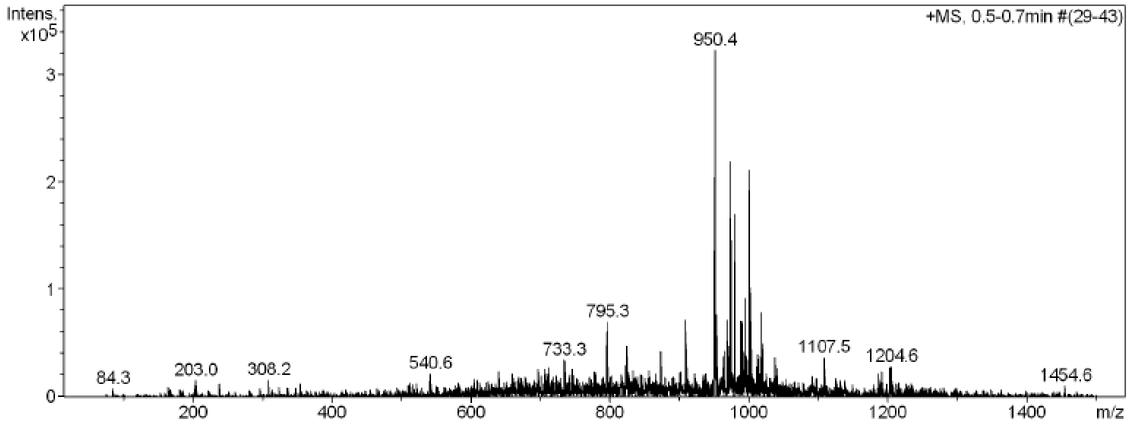
Intens.
x10^5
+MS, 0.5-0.7min #(29-43)
950.4
795.3
733.3
540.6
84.3
203.0
308.2
1107.5
1204.6
1454.6
m/z

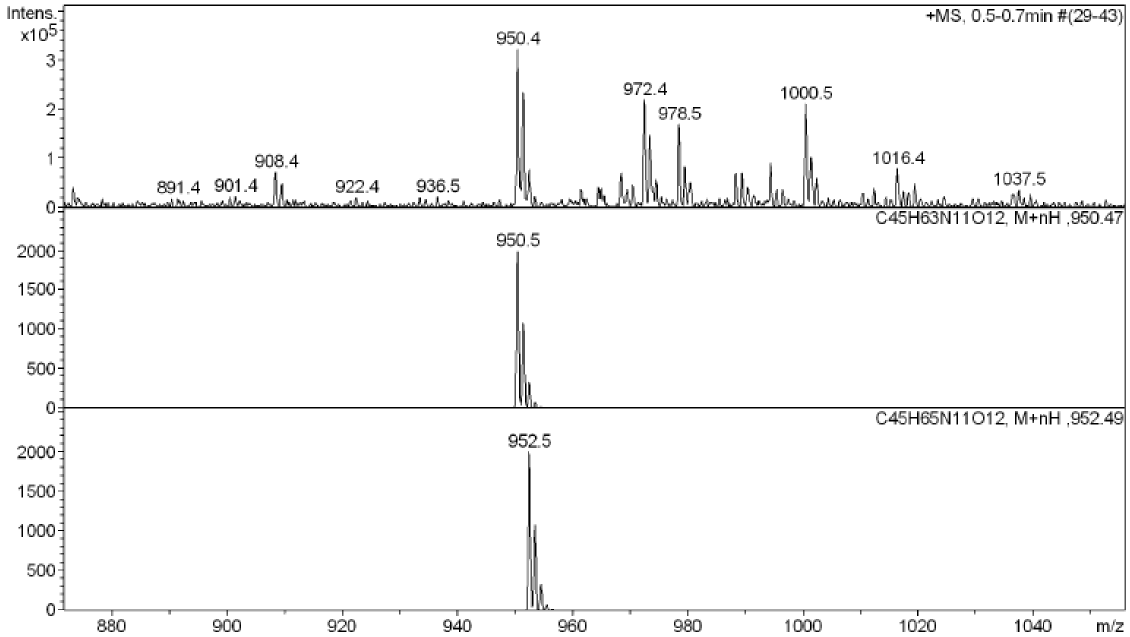
Intens.
x10^5
+MS, 0.5-0.7min #(29-43)
950.4
972.4
978.5
1000.5
908.4
891.4
901.4
922.4
936.5
1016.4
1037.5
C45H63N11O12, M+nH ,950.47
950.5
C45H65N11O12, M+nH ,952.49
952.5
m/z

Biaryl cyclic peptide 9

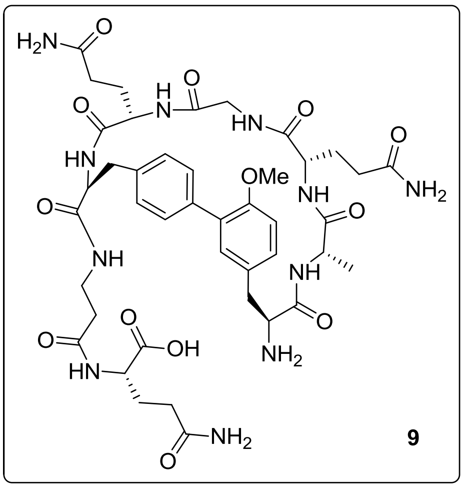

9

HPLC (λ = 220 nm)

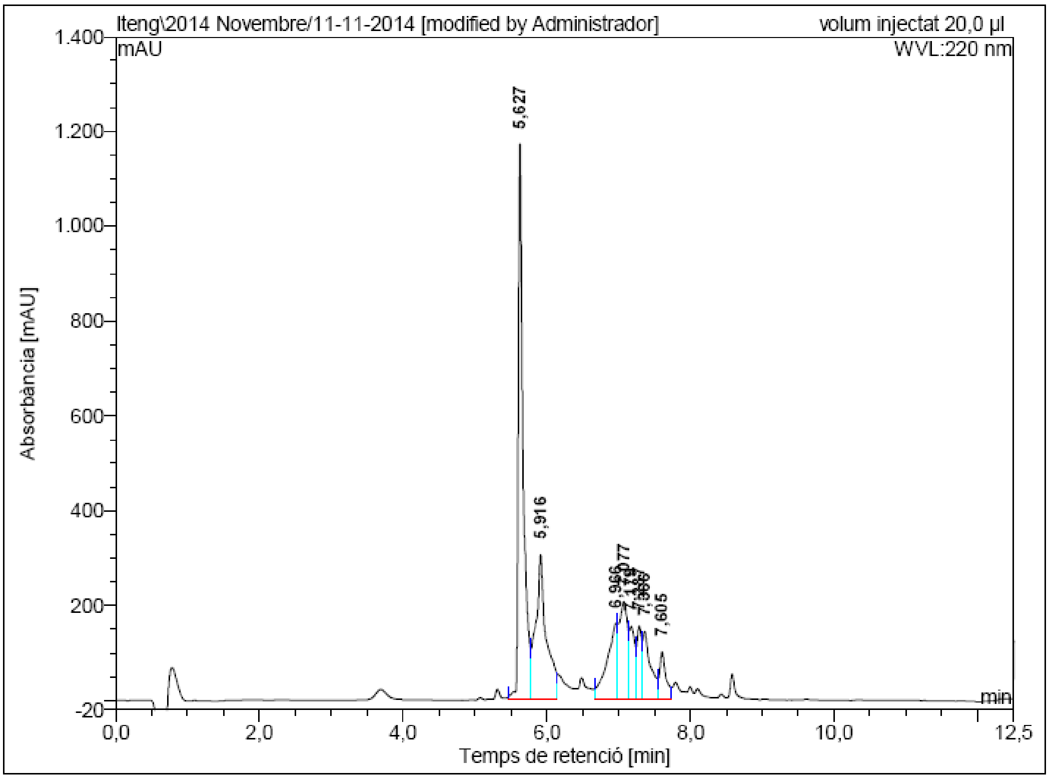

No.	Temps retenció min	alçada mAU	Area mAU*min	Area relativa %
1	5,63	1172,287	90,776	35,80
2	5,92	306,356	52,987	20,89
3	6,97	161,377	26,929	10,62
4	7,08	206,978	28,140	11,10
5	7,18	154,595	13,317	5,25
6	7,29	155,577	11,478	4,53
7	7,37	144,208	19,127	7,54
8	7,60	100,922	10,842	4,28
Total:		2402,301	253,598	100,00

ESI-MS *m/z*

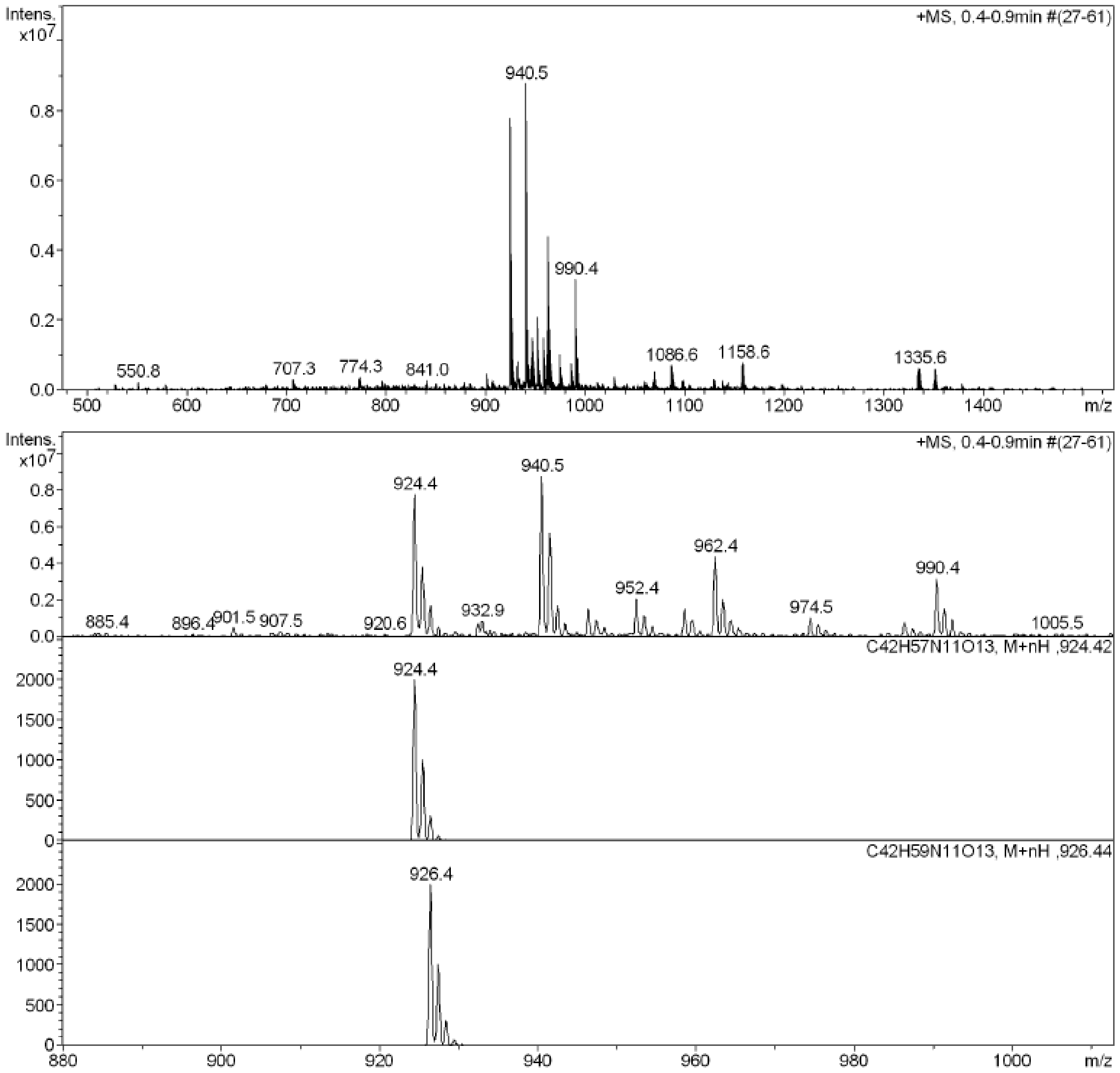
Intens. x10^7
+MS, 0.4-0.9min #(27-61)
940.5
990.4
550.8
707.3
774.3
841.0
1086.6
1158.6
1335.6
m/z
924.4
932.9
952.4
962.4
974.5
885.4
896.4
901.5
907.5
920.6
1005.5
C42H57N11O13, M+nH ,924.42
C42H59N11O13, M+nH ,926.44
926.4

Biaryl cyclic peptide 12

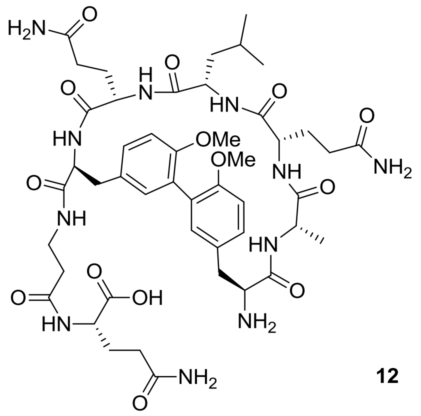

12

HPLC (λ = 220 nm)

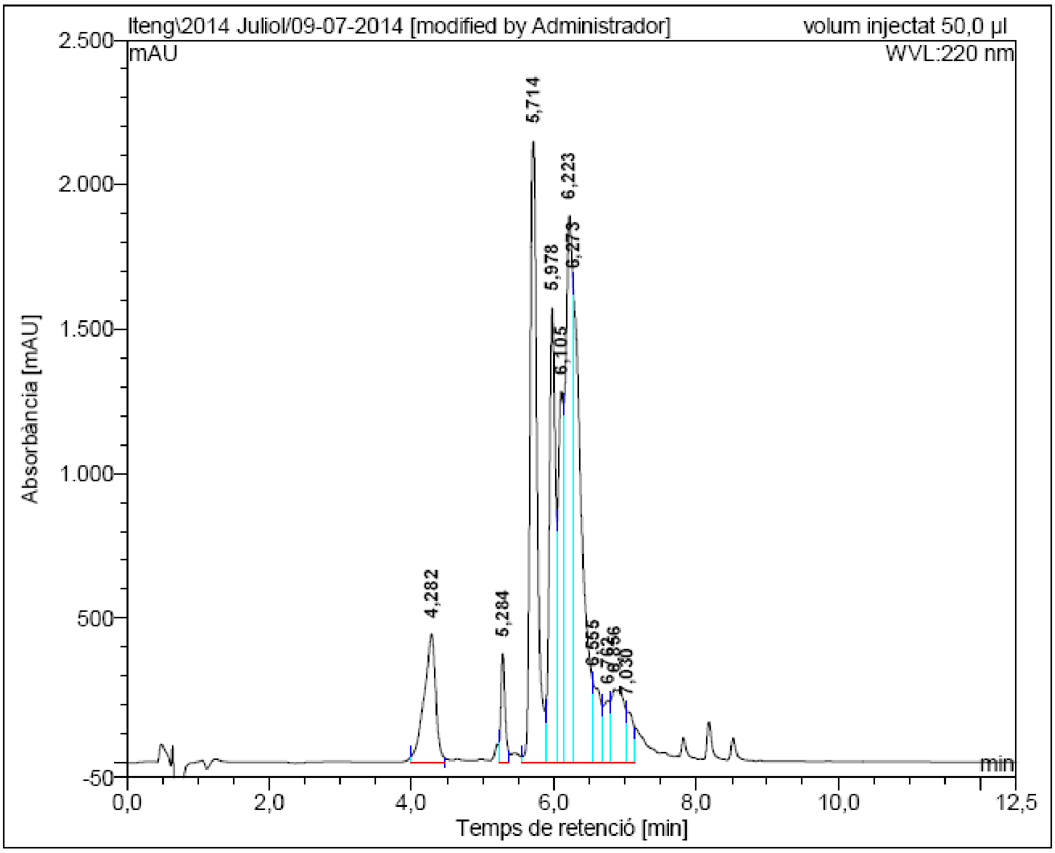

No.	mps retenc min	alçada mAU	Area mAU*min	Area relativa %
1	4,28	443,481	84,118	7,03
2	5,28	375,948	26,007	2,17
3	5,71	2147,748	246,370	20,58
4	5,98	1571,558	160,207	13,38
5	6,11	1282,747	97,631	8,16
6	6,22	1890,043	220,412	18,41
7	6,27	1646,613	235,950	19,71
8	6,56	274,338	30,443	2,54
9	6,76	213,917	23,673	1,98
10	6,86	253,748	54,173	4,53
11	7,03	175,102	17,986	1,50
Total:		10275,243	1196,971	100,00

ESI-MS *m/z*

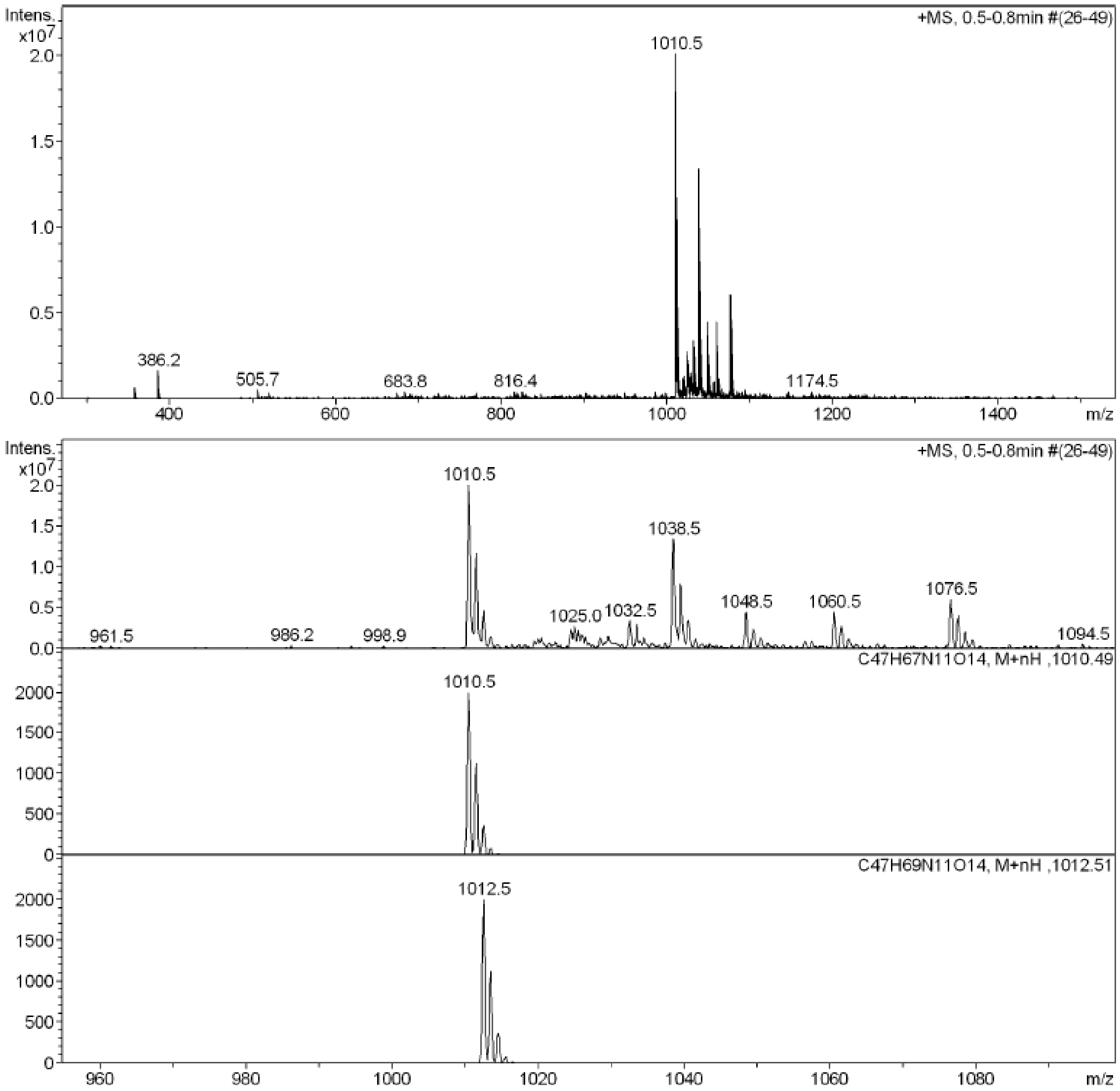

Intens. x10^7
+MS, 0.5-0.8min #(26-49)
1010.5
386.2
505.7
683.8
816.4
1174.5
m/z
1038.5
1025.0
1032.5
1048.5
1060.5
1076.5
961.5
986.2
998.9
1094.5
C47H67N11O14, M+nH ,1010.49
C47H69N11O14, M+nH ,1012.51
1012.5

Biaryl bicyclic peptide 1

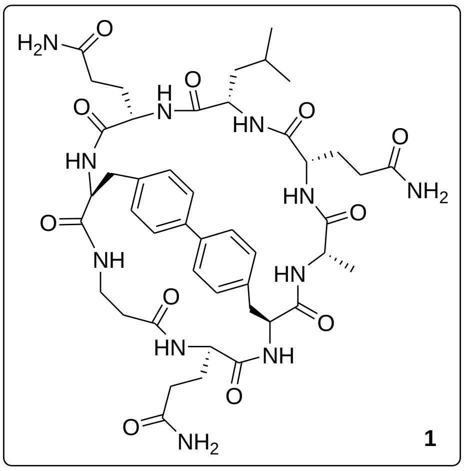

1

HPLC (λ = 220 nm)

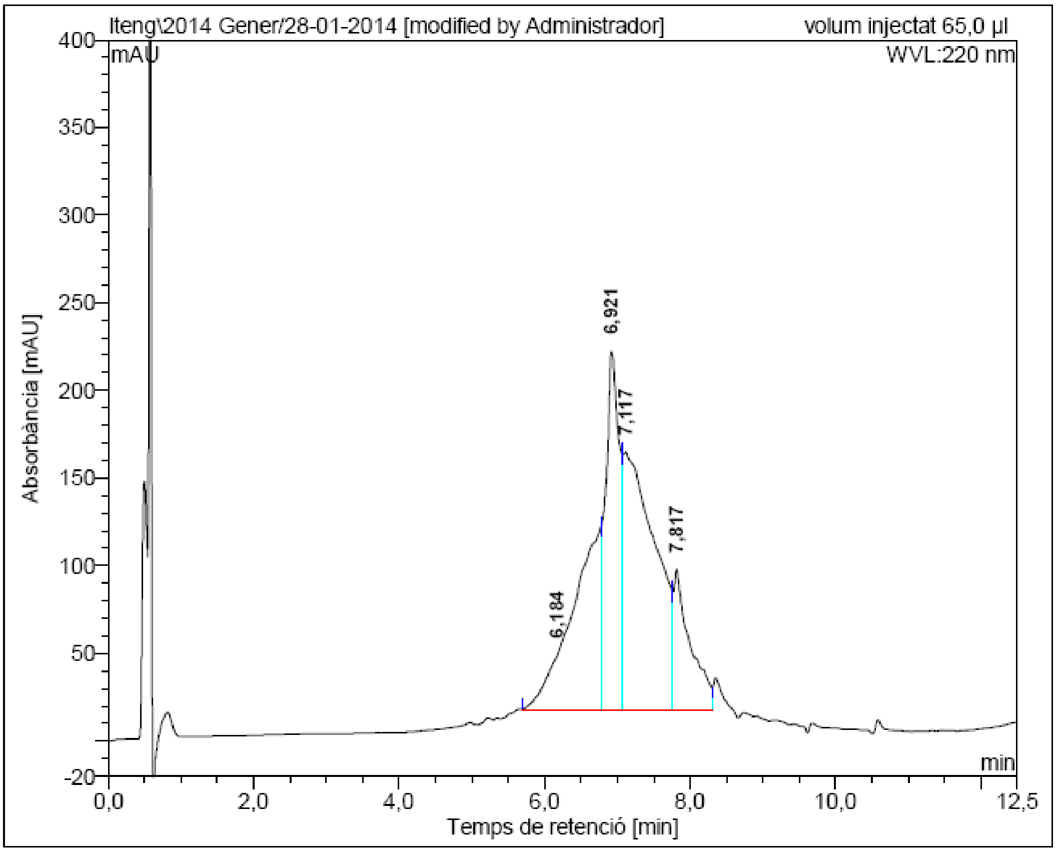

No.	mps retenc min	alçada mAU	Area mAU*min	Area relativa %
1	6,18	31,344	47,202	24,83
2	6,92	203,859	44,684	23,51
3	7,12	146,649	76,901	40,46
4	7,82	79,824	21,275	11,19
Total:		461,677	190,063	100,00

ESI-MS *m/z*

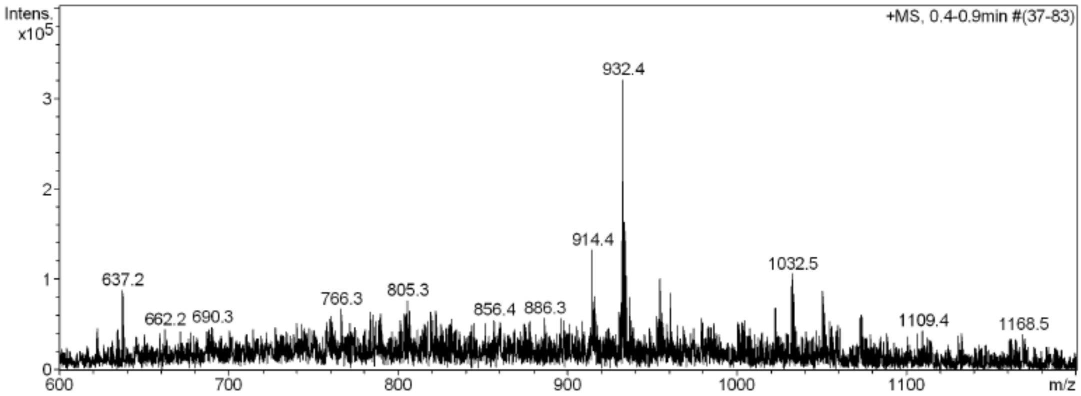

ESI-MS2 (932.4) *m/z*

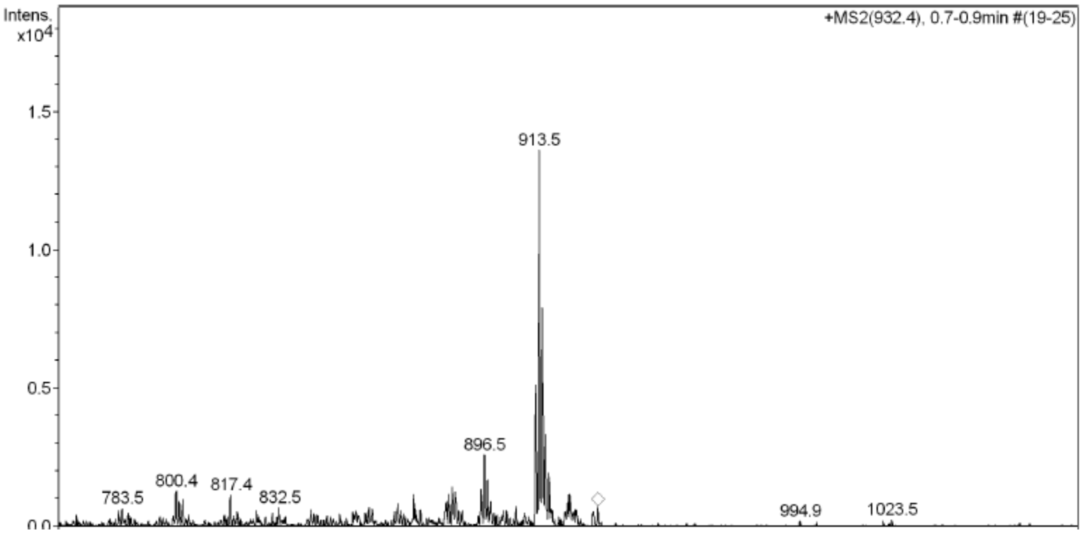

HPLC - MS (ESI) *m/z*

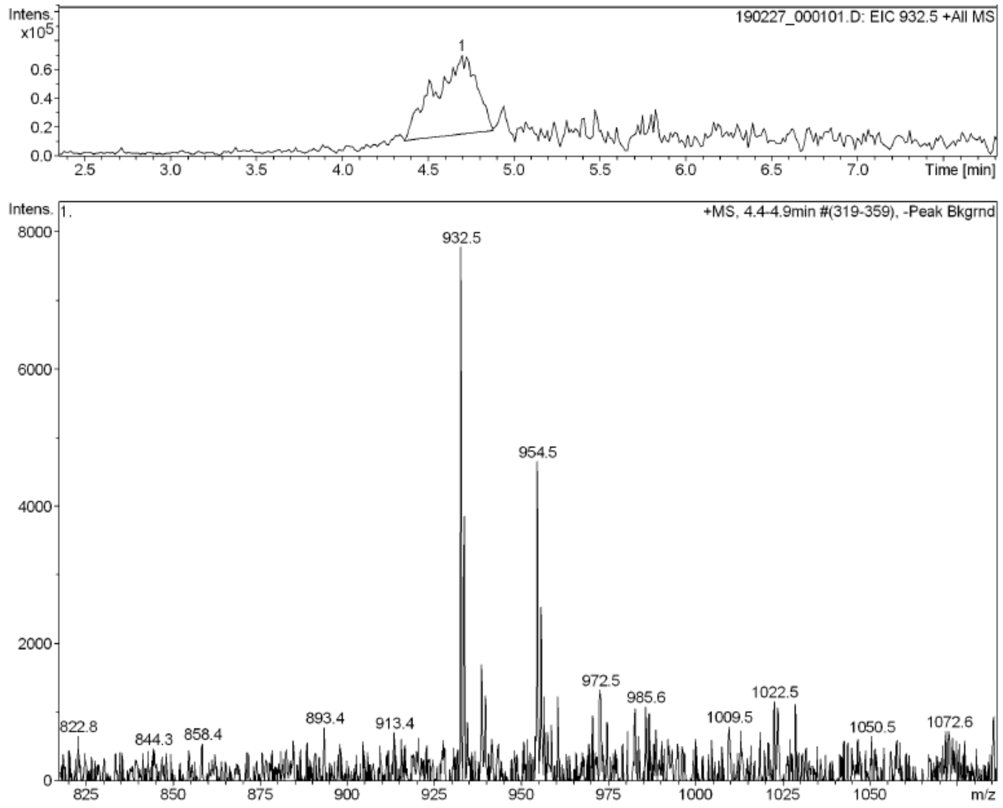

Intens. x10^5
190227_000101.D: EIC 932.5 +All MS
Time [min]
Intens.
+MS, 4.4-4.9min #(319-359), -Peak Bkgrnd
932.5
954.5
972.5
985.6
1022.5
1009.5
1050.5
1072.6
822.8
844.3
858.4
893.4
913.4
m/z

HRMS (ESI) *m/z*

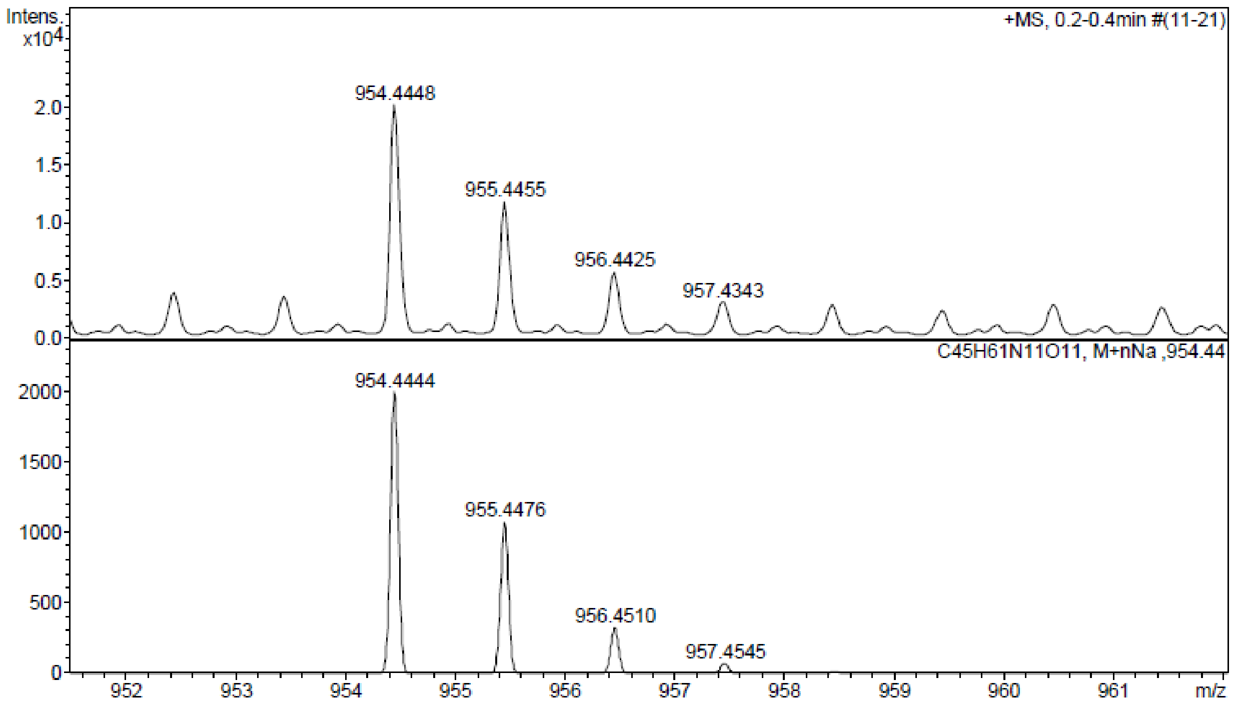
Intens.
x10^4
+MS, 0.2-0.4min #(11-21)
954.4448
955.4455
956.4425
957.4343
C45H61N11O11, M+nNa ,954.44
954.4444
955.4476
956.4510
957.4545
m/z

Biaryl bicyclic peptide 2

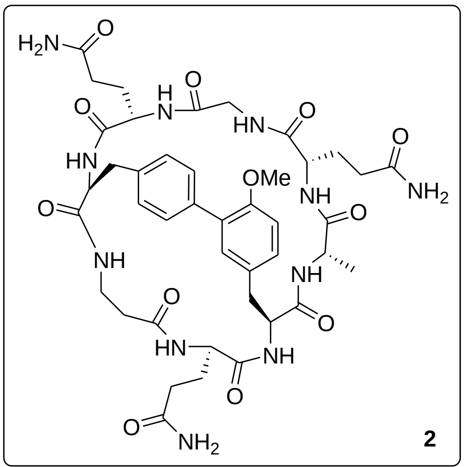

2

HPLC (λ = 220 nm)

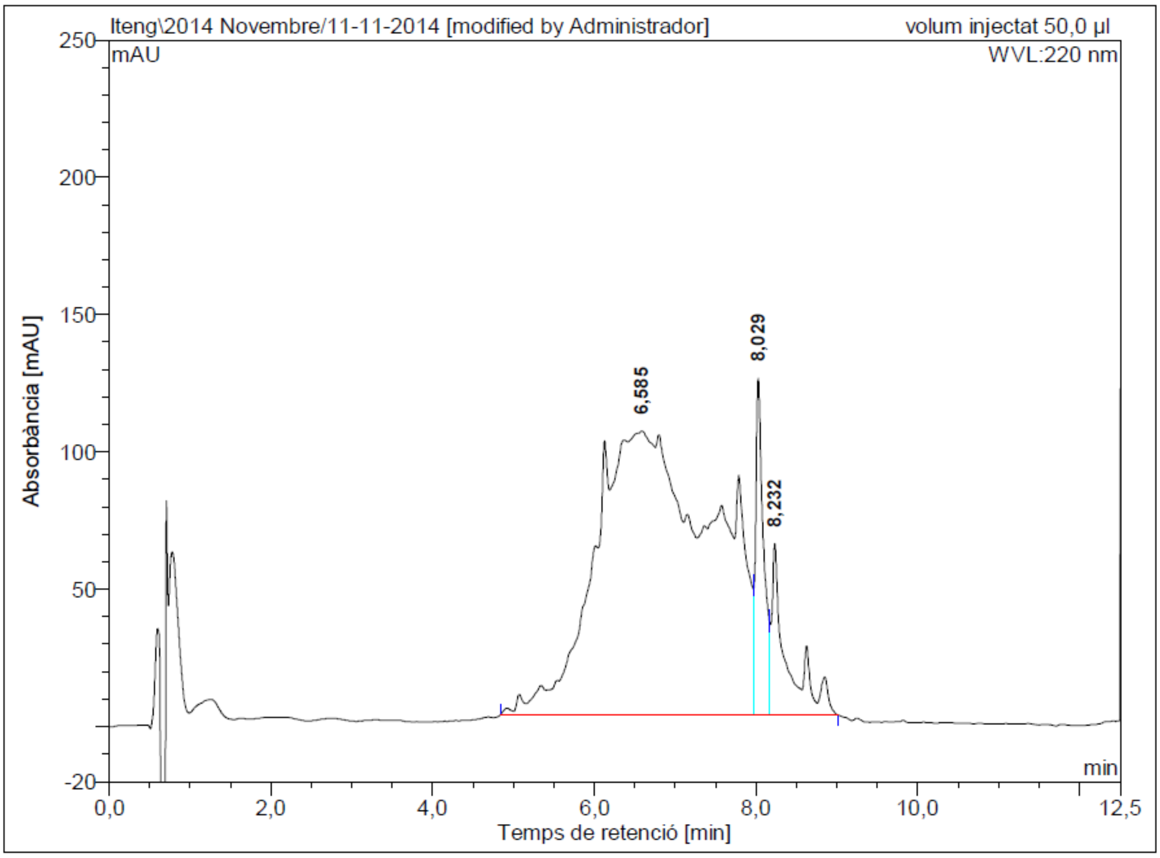

No.	Temps retenció min	alçada mAU	Area mAU*min	Area relativa %
1	6,58	103,199	176,618	86,53
2	8,03	122,355	13,697	6,71
3	8,23	62,188	13,786	6,75
Total:		287,743	204,101	100,00

ESI-MS *m/z*

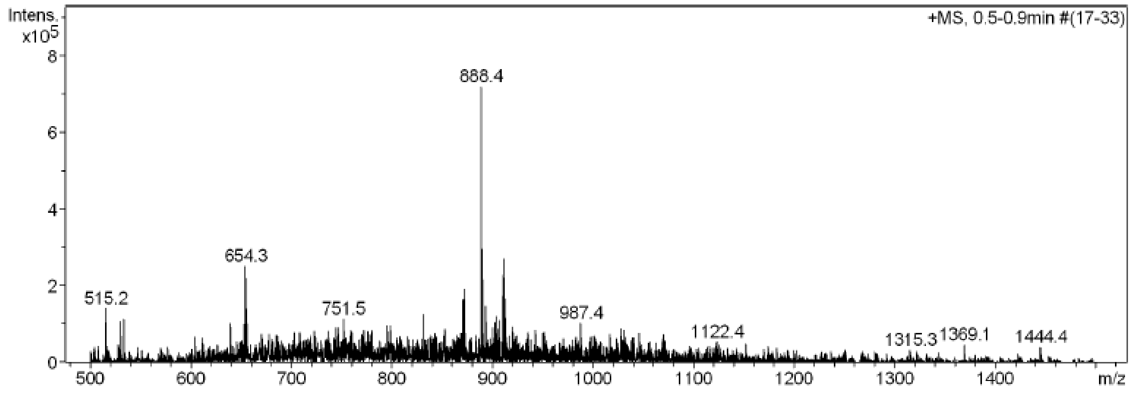

HRMS-ESI *m/z*

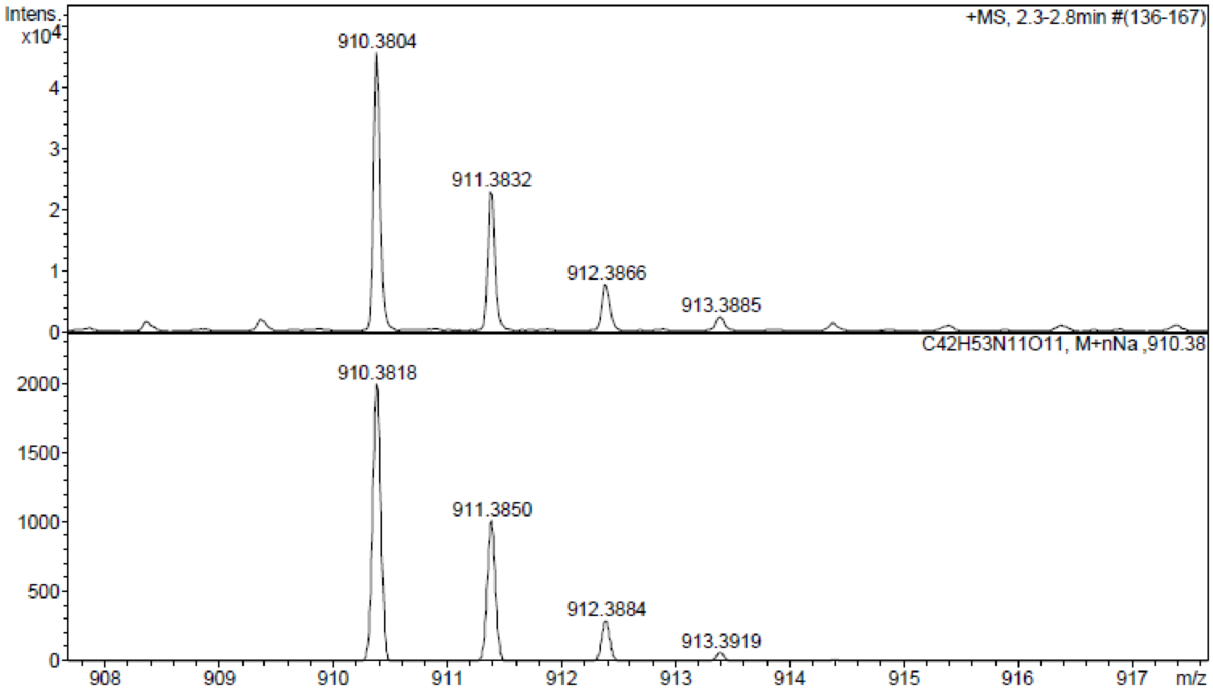

Biaryl bicyclic peptide 3

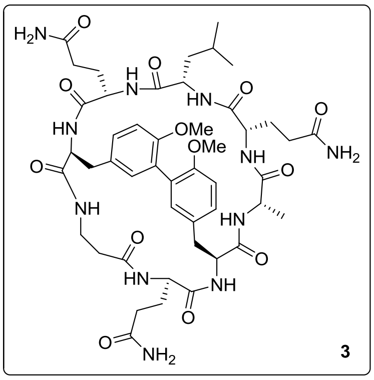

HPLC (λ = 220 nm)

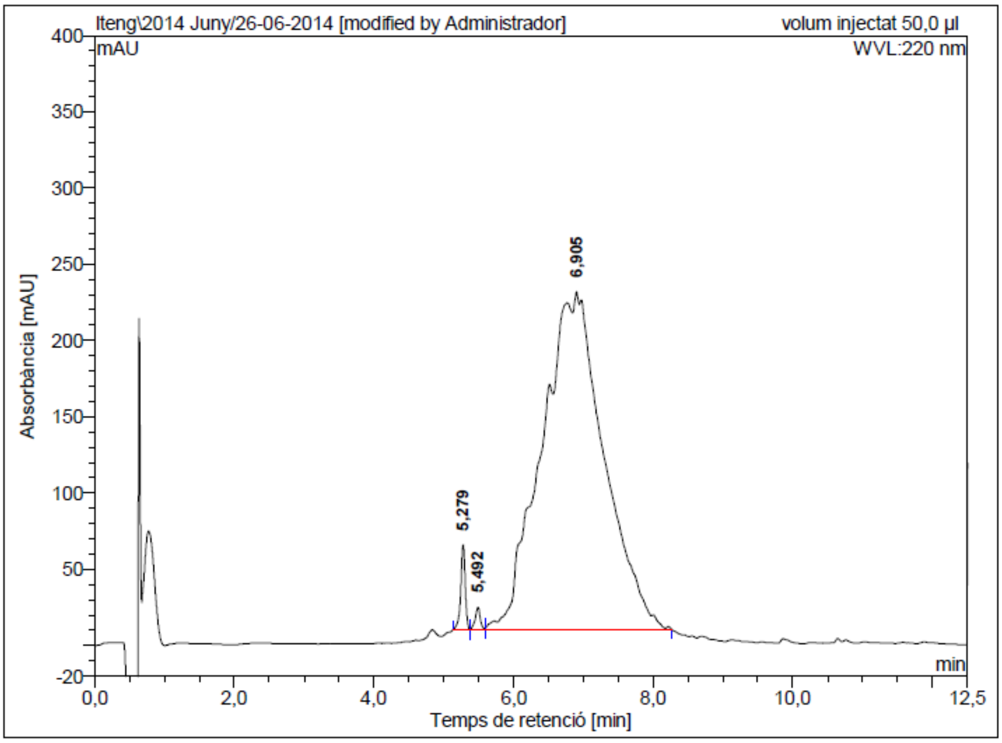

No.	Temps retenció min	alçada mAU	Area mAU*min	Area relativa %
1	5,28	55,926	4,215	1,79
2	5,49	15,134	1,317	0,56
3	6,90	221,751	230,347	97,65
Total:		292,810	235,880	100,00

ESI-MS *m/z*

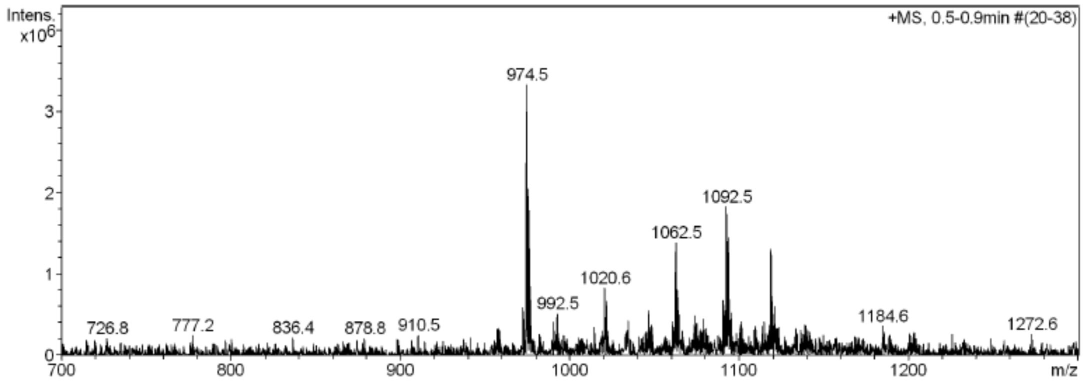

ESI-MS2 (992.5) *m/z*

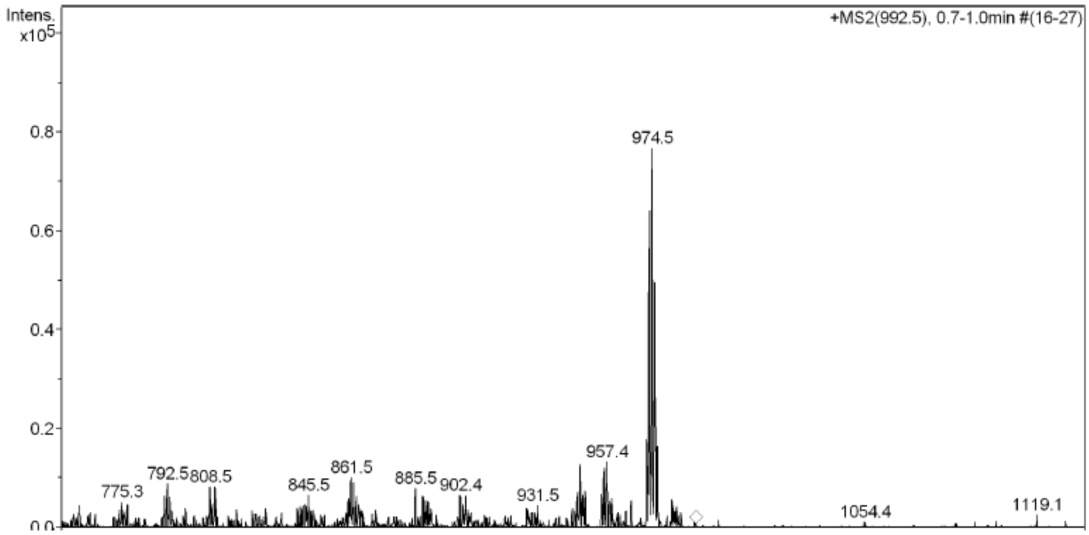

HPLC - MS (ESI) *m/z*

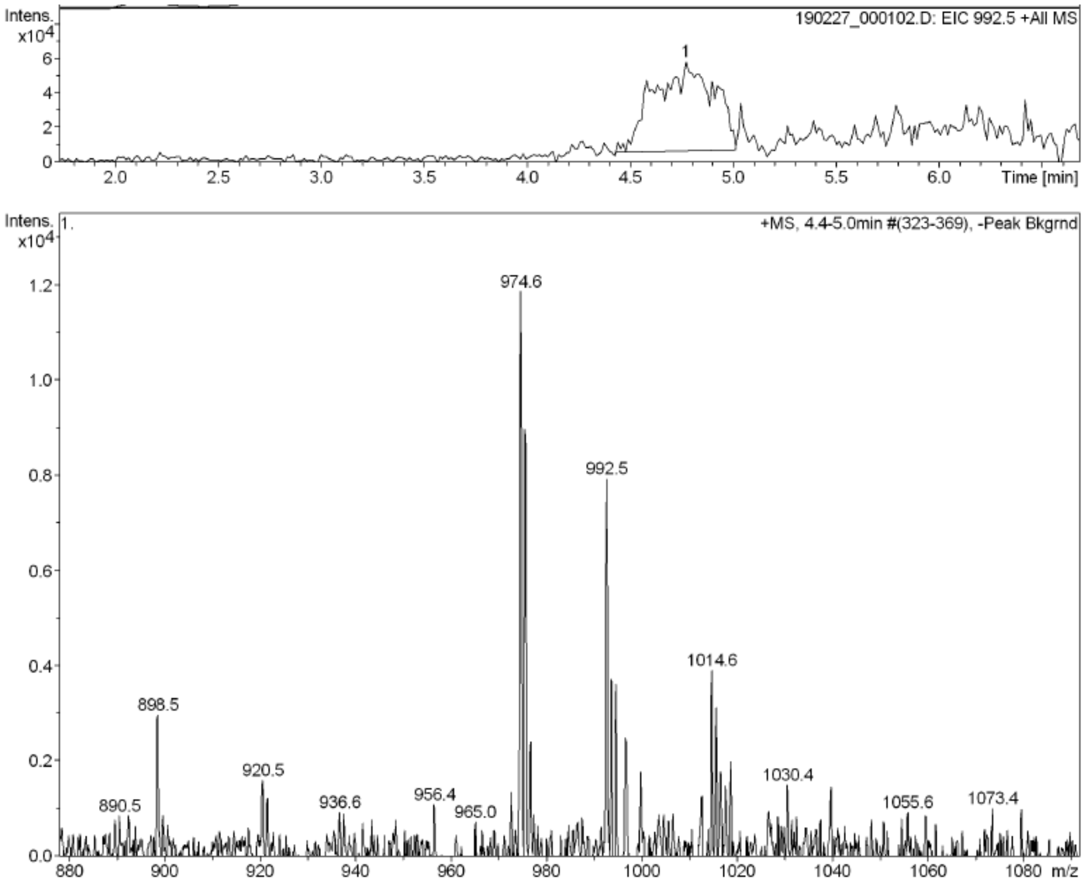

HRMS-ESI *m/z*

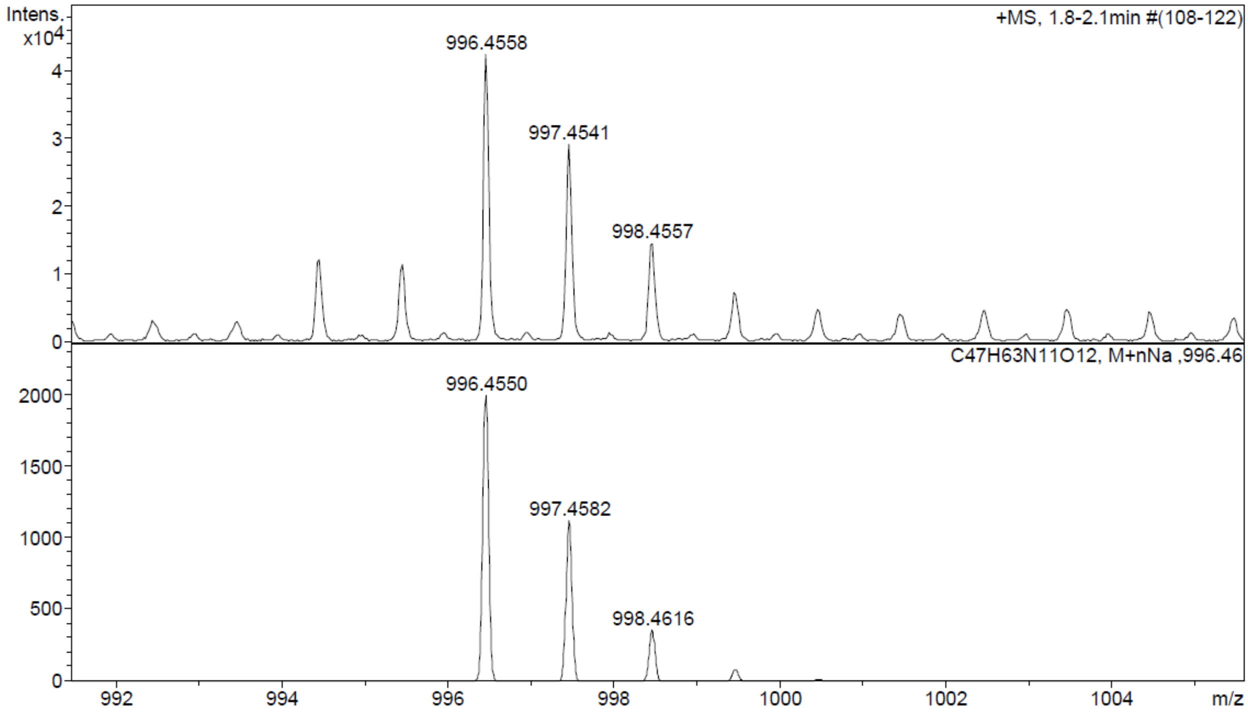

Supplement: File 1 — Experimental, synthesis, and characterization of all the compounds. [file Beilstein_J_Org_Chem-15-761-s001.pdf]
